# Common and Distinct Features in Serum Proteomic Profiles in Keratoconus, Post-Laser Vision Correction Ectasia, and Pellucid Marginal Degeneration

**DOI:** 10.1167/iovs.67.3.23

**Published:** 2026-03-11

**Authors:** Katarzyna Jaskiewicz-Rajewicz, Alicja Wysocka, Eliza Matuszewska-Mach, Natalia Rzetecka, Magdalena Maleszka-Kurpiel, Jakub Wozniak, Andrzej Michalski, Monika Udziela, Jacek P. Szaflik, Rafal Ploski, Malgorzata Rydzanicz, Jan Matysiak, Marzena Gajecka

**Affiliations:** 1 Institute of Human Genetics, Polish Academy of Sciences, Poznan, Poland; 2 Poznan University of Medical Sciences, Chair and Department of Inorganic and Analytical Chemistry, Poznan, Poland; 3 Optegra Eye Health Care Clinic in Poznan, Poznan, Poland; 4 Poznan University of Medical Sciences, Chair of Ophthalmology and Optometry, Poznan, Poland; 5 Poznan University of Medical Sciences, Chair and Department of Genetics and Pharmaceutical Microbiology, Poznan, Poland; 6 Initium BioData, Kościelna Wieś, Poland; 7 Department of Ophthalmology, Medical University of Warsaw, Warsaw, Poland; 8 SPKSO Ophthalmic University Hospital, Warsaw, Poland; 9 Department of Medical Genetics, Medical University of Warsaw, Warsaw, Poland

**Keywords:** proteome, serum, keratoconus (KTCN), post-laser vision correction (PLVC) ectasia, pellucid marginal degeneration (PMD)

## Abstract

**Purpose:**

Both intrinsic and extrinsic factors are implicated in keratoconus (KTCN), pellucid marginal degeneration (PMD), and post-laser vision correction (PLVC) ectasia etiology. Serum proteomic profiling was performed to molecularly differentiate the ectasia and to assess systemic components in their pathophysiology.

**Methods:**

Serum samples from 93 patients with KTCN, 10 patients with PLVC, 4 patients with PMD, and 44 non-ectatic controls were profiled using matrix-assisted laser desorption ionization-time of flight tandem mass spectrometry (MALDI-TOF/TOF MS/MS). Clinical and environmental variables (e.g. allergy/atopy/asthma and eye rubbing intensity), together with ophthalmologic parameters (K1, K2, Kmax, TCT, and anterior/posterior elevation), were assessed in the principal component analysis (PCA), Weighted Gene Co-expression Network Analysis (WGCNA), linear modeling, and other assessments. Serum m/z features were correlated with RNA-seq data from corneal epithelium (CE) samples of 31 individuals.

**Results:**

As findings of the PCA, associations were observed for atopy and/or asthma (estimate = −5.52, *P* = 0.03), PMD diagnosis (estimate = 8.3, *P* = 0.04), and age (estimate = −0.14, *P* = 0.01), with observable trends for KTCN status and intense eye rubbing. Correlations between clinical variables and individual m/z features indicated that posterior elevation, reflecting KTCN severity, was associated with an ATP5B protein-assigned peak (m/z 1921.9655, ρ = −0.31, adjusted *P* = 0.016). In the integration analyses, correlations between serum peaks and corresponding CE gene expression, including APOH- (ρ = 0.43, *P* = 0.017) and ALB-assigned peaks (ρ = 0.35–0.42, *P* < 0.05) were identified. Pairwise eigengene comparisons showed negative correlation between KTCN and non-ectatic controls (Pearson *r* = −0.89, *P* = 0.007).

**Conclusions:**

The local ocular pathology is reflected in serum proteomic profiles, together with accompanying systemic inflammation and intense eye-rubbing behavior. Common proteomic features of different types of corneal ectasia may reflect overlapping pathophysiological mechanisms.

Corneal ectatic disorders represent a heterogeneous group of conditions characterized by progressive corneal thinning, biomechanical weakening, and irregular surface geometry that all contribute to refractive error and loss of visual acuity. Keratoconus (KTCN), the most common, typically affects adolescents or young adults and often follows a bilateral but asymmetric course that may progress to advanced stages requiring keratoplasty.^1^^,^^2^ The reported prevalence of KTCN varies widely across populations and depends strongly on ethnicity and diagnostic criteria. In the comprehensive meta-analysis, the global prevalence was estimated at approximately 1.38 per 1000 population; however, individual population-based studies have reported values ranging from very low prevalence in some Western populations (approximately 0.1–0.2 per 1000) to several tens per 1000 in certain Middle Eastern and Asian populations.^3^^,^^4^ Because of this variability, it has been suggested that KTCN encompasses a spectrum of phenotypically similar conditions, rather than a single uniform disease entity.^3^ In contrast, post-laser vision correction ectasia (PLVC), a serious complication observed in 0.033% to 0.66% of corneal refractive surgeries, is diagnosed at a later patient age, months to years after the primary refractive procedure.^5^ In the case of pellucid marginal degeneration (PMD), another type of corneal ectasia, which manifests with inferior peripheral thinning and a characteristic topographic pattern, studies have reported an average age of presentation between 34 and 48 years old.^6^^,^^7^

Although the clinical phenotypic similarities and differences among KTCN, PMD, and PLVC are well-documented,^8^ the underlying molecular mechanisms and systemic manifestations that accompany the structural changes in the cornea, especially for PMD and PLVC, remain incompletely delineated. A deeper understanding of the molecular pathology is essential for accurate risk stratification, the identification of reliable biomarkers, and the development of targeted interventions to halt or prevent disease progression.

Accumulating evidence from populations with different genetic ancestries and environmental backgrounds implicate both intrinsic and extrinsic causative factors in the pathogenesis of KTCN.^9^^,^^10^ Genetic factors, including alterations in extracellular matrix (ECM) composition, affect corneal biomechanics and contribute to its weakening.^11^^,^^12^ Still, environmental and behavioral factors, especially the repetitive mechanical microtrauma from the eye rubbing, are strongly associated with both disease onset and progression. Chronic eye rubbing can produce focal stromal deformation, upregulate matrix metalloproteinases, and pro-inflammatory mediators, and sustain abnormal wound-healing responses.^13^ Simultaneously, a history of allergy or atopy frequently co-occurs with eye rubbing, and with difficult-to-measure risk behaviors, such as nocturnal eye rubbing or compression,^3^^,^^4^^,^^14^^–^^16^ complicating/obscuring causal inference but strengthening the scientific rationale for verifying a link in disease biology.

In contrast, the pathophysiology of PLVC remains largely unknown, with only clinical risk factors identified, such as younger age (<30 years), a family history of KTCN, topographic or tomographic corneal abnormalities resembling forme fruste KTCN, thinner preoperative corneas (<500 µm), thinner residual stromal beds (<300 µm), excessive stromal ablation (>100 µm), a high percentage of tissue altered (>40%), and high myopia.^17^^–^^19^ To date, no genetic risk factors have been reported. Molecular data/findings regarding PLVC are limited, but elevated levels of cytokines and chemokines in the tear film have been reported,^20^ as well as transcriptomic and proteomic similarities between the corneal epithelium (CE) in PLVC and KTCN.^5^

Even more clinically challenging is the differentiation of KTCN with inferior localization and PMD, which is characterized by inferior corneal thinning and a “crab-claw” pattern on anterior curvature topography. Despite the phenotypic similarities, a distinction between these two corneal disorders is essential due to differences in prognosis and clinical management. For example, therapeutic approaches, such as corneal cross-linking, intrastromal corneal ring segment implantation, or keratoplasty, appropriate for inferior KTCN, may yield substantially different outcomes in PMD.^21^ With respect to the genetic background of PMD, no evidence of inheritance has been reported to date.^22^ However, the co-occurrence of PMD with systemic disorders such as Sjögren's syndrome,^23^ scleroderma,^24^ and ichthyosis,^25^ has been described, further supporting a possible association between PMD and environmental or mechanical factors, including eye rubbing. As PMD is a rare condition,^22^ these observations should be further validated in larger cohort studies.

Previous applications of matrix-assisted laser desorption/ionization time-of-flight mass spectrometry (MALDI-TOF MS) to clinical proteomics have demonstrated its ability to detect distinctive and/or specific profiles. Proteomic profiling in ocular diseases has predominantly focused on tears. In studies utilizing liquid-chromatography tandem mass spectrometry (LC-MS/MS) and MALDI approaches, some alterations in tear proteins and peptides in KTCN, including abnormalities in ECM proteins, inflammatory mediators, and proteins related to oxidative stress and cellular metabolism, have been reported.^26^^–^^31^ In contrast, serum proteomic profiling in corneal ectasias remains largely underexplored, despite its significant potential as a minimally invasive and easily standardizable approach for identifying systemic biomarkers that may reflect disease onset or progression.

In this study, serum tandem MALDI-TOF/TOF MS/MS profiling was conducted to address the following scientific questions: (i) in the aspect of diagnostic discrimination, whether serum peptide profiles differ or are common among patients with KTCN, PLVC, and PMD, in comparison with non-ectatic controls; (ii) concerning behavioral and environmental associations, whether clinical exposures such as intense eye rubbing or allergy/atopy are accompanied by distinct and reproducible serum m/z profiles; and (iii) toward clinical and molecular relationship, whether specific peptide/protein fragments peaks in serum demonstrate measurable correlations with corneal epithelial gene expression profiles and constitute indicators of disease severity (including the features of increased posterior elevation and corneal thinning). Therefore, the objective of this exploratory study was to compare and differentiate serum m/z peaks in the assessed three ectasias and elucidate their relationships with both the ophthalmologic phenotypes and molecular alterations within the CE, thereby providing further insight into systemic components of corneal ectatic disease pathophysiology.

## Materials and Methods

### Patients and Clinical Evaluation

The study protocol was approved by the Bioethics Committee at Poznan University of Medical Sciences, Poznan, Poland (decisions no. 453/14, 755/19, and 180/24). Informed consent was obtained from all participants, according to the Declaration of Helsinki. Study participants were recruited at the Optegra Eye Health Care Clinic in Poznan, Poland, and the Department of Ophthalmology, Medical University of Warsaw, Poland, between October 2019 and July 2024. All participants self-reported European ancestry. Formal genetic ancestry testing was not performed. Each patient with KTCN, PMD, and PLVC, and non-ectatic control individuals, underwent a complete ophthalmological examination, including corneal tomography and epithelial thickness mapping, as previously described.^5^ KTCN was diagnosed based on corneal tomography with rotating Scheimpflug camera WaveLight Oculyzer II (Alcon, TX, USA) or Pentacam (Oculus Optikgeraete GmbH, Wetzlar, Germany). The inclusion and exclusion criteria for patients with KTCN, patients with PLVC, and non-ectatic control individuals (individuals with mild myopia and no clinical signs of corneal ectasia) were consistent with the previously established criteria.^5^^,^^13^ PMD inclusion criteria embraced a topographic “crab claw” pattern where the thinnest corneal area is located immediately below the area of greater corneal curvature, with topographic, aberrometric, and tomographic parameters similar to KTCN, taking into account a greater mean distance from the corneal center to the minimum thickness point.^32^^,^^33^ To discriminate the KTCN and PMD, according to the Global Consensus on Keratoconus and Ectatic Diseases,^34^ a combination of approaches, including full slit-lamp examination, corneal thickness map, anterior curvature map, and anterior tomographic elevation map, was utilized.

Each study participant completed a detailed survey, which, as in our previous studies,^13^^,^^35^ included questions about demographics, behaviors, environment, and socioeconomic aspects. In the case of the eye rubbing aspects, we used the multiple-question method (including photographs of the representative eye rubbing patterns)^35^ to minimize bias in recalling the behaviors. Eye rubbing was classified as intense based on the criteria of rubbing force (knuckles/base of hand/whole palm/fist versus fingers) and the area of the eye rubbed (larger area of the eye: upper/lower eyelids versus inner/outer eye corners).

### Sample Processing and MALDI-TOF/TOF MS/MS

Blood was collected into serum tubes, processed by double centrifugation, aliquoted, and stored at −80°C until analysis. Prior to MS, serum aliquots were subjected to the in-solution tryptic overnight digestion, and then concentrated, desalted, and purified using C18 ZipTip microcolumns; eluates were mixed with α-cyano-4-hydroxycinnamic acid matrix and spotted in triplicate onto AnchorChip (Bruker Daltonics) plates. MALDI-TOF spectra were acquired in the reflectron-positive mode on a Bruker UltrafleXtreme instrument across m/z 700 to 3500 using 2000 laser shots per spectrum. External calibration was applied, and mass accuracy was monitored (average mass deviation = ≤1 ppm). For peak identification, selected samples underwent MALDI-TOF/TOF MS/MS following in-solution tryptic digestion and database searches against SwissProt.

### Data Preprocessing

Spectra were exported to tabular form and normalized according to Total Ion Current (TIC). M/z peaks missing in >30% of samples were removed. For the retained peaks, missing intensities were imputed as one-half of the minimum intensity observed for that peak across all samples. Before the statistical analyses, zero-variance features were removed.

### Integration of Proteomic and RNA-Seq Outputs

For data integration purposes, we incorporated previously generated bulk RNA-seq data from the central topographic region of the CE, corresponding to the apex of the keratoconic cone.^5^ That assessment included 31 individuals (16 patients with KTCN, 7 patients with PLVC, and 8 non-ectatic control individuals) whose serum samples were also analyzed in the present study, enabling direct multi-omic correlation between epithelial gene expression and serum peptide profiles.

### Statistical Analyses

For statistical analyses, ophthalmological data from one eye per individual were used. For the patients with corneal ectasia, the eyes with more advanced disease were selected. For example, in KTCN, although the condition is bilateral, it is highly asymmetric, with one eye typically more affected than the other. Similarly, ectasia secondary to PLVC often occurs in only one eye.^36^^,^^37^ Descriptive statistics for clinical variables, including means, standard deviations, and medians, were calculated. Missing clinical data were not imputed. As a result, the sample size varied slightly between the following analyses, depending on the availability of clinical parameters, as indicated in Results. Normality of distributions was assessed using Shapiro–Wilk tests. For nonparametric comparisons across more than two groups, Kruskal–Wallis tests were performed, followed by Dunn's post hoc comparisons. Nominal and categorical variables were analyzed using chi-square tests in contingency tables. For multiple comparisons of clinical parameters, *P* values were adjusted using the Holm–Bonferroni correction. These analyses were conducted in JASP software.^38^

All computations, embracing proteomic and clinical data, were performed in R environment (RStudio version “2021.9.0.351”)^39^ with the use of packages: tidyverse,^40^ rstatix,^41^ broom,^42^ pheatmap,^43^ ggplot2,^44^ and ggrepel.^45^ Principal Component Analysis (PCA) was conducted with data scaling (z-score standardization of mean-centered proteomic data). For visualization, PCA scatter plots were generated for multiple grouping variables, including diagnosis, eye rubbing, intense eye rubbing, atopy and/or asthma, sex, and smoking. Ellipses representing 95% confidence intervals (calculated using the t-distribution) were added for groups containing three or more samples. To assess associations between PCA components and clinical covariates, linear regression models were fitted with PC1 and PC2 as dependent variables. Only complete cases for the dependent variables and covariates were included. For each model, regression coefficients, 95% confidence intervals, and standard errors were calculated.

Per-feature statistical testing was performed for the following subgroups comparisons: KTCN versus non-ectatic controls, PLVC versus non-ectatic controls, PMD versus non-ectatic controls, KTCN versus PLVC, KTCN versus PMD, and PLVC versus PMD, as well as for the binary clinical covariates: severe KTCN (defined as at least one eye with Topographic Keratoconus Classification ≥ 3), sex, eye rubbing, intense eye rubbing, allergy, atopy and/or asthma, smoking cigarettes, and presence of dust in the working environment. For each comparison, the Mann–Whitney *U* (Wilcoxon rank-sum) test statistics and rank-biserial effect sizes were computed, alongside unadjusted *P* values, Benjamini–Hochberg false discovery rate (FDR)–adjusted q-values, group medians, and log_2_ median ratios (log_2_FC) for all features. Given the characteristics of MALDI-MS data, features were classified as biologically meaningful when they met both criteria: |log_2_FC| > 0.3785 (corresponding to ≥1.3-fold increases or ≤1/1.3-fold decreases) and *P* value < 0.05 (uncorrected *P* values were used to identify features of statistical significance and not to lose biologically significant changes, as correction for multiple testing was considered too conservative at this stage given the limited subgroups’ sizes). To assess the robustness of per-feature Mann–Whitney *U* test results, a nonparametric bootstrap procedure (1000 resamples) was applied.^46^ Group-wise resampling with replacement was performed while preserving original sample sizes, and the proportion of bootstrap iterations yielding *P* < 0.05 was used as a measure of result stability.

Spearman’s rank correlation coefficients were computed for each continuous clinical variable and each m/z peak intensity. In addition, for m/z peaks assigned to specific proteins, their intensities were correlated with matched corneal epithelial gene-expression levels (transcripts per million [TPM]) using the Spearman method. Uncertainty of Spearman correlations results was assessed using the nonparametric bootstrap 95% confidence intervals (2000 resamples).

Weighted Gene Co-expression Network Analysis (WGCNA) was performed in R software using the WGCNA package.^47^ Prior to network construction, all m/z peak intensities were Z-score scaled across samples. Sample relationships were assessed by hierarchical clustering using Euclidean distance and average linkage, and visualized as a sample dendrogram to screen for outliers. A signed co-expression network was constructed using a soft-thresholding power of 6, selected according to the scale-free topology criterion. Modules, clusters of m/z peaks (protein or peptide fragments) that exhibit highly correlated intensity patterns across samples and may be involved in shared biological processes or clinical traits, were detected using dynamic tree cutting with a minimum module size of 10. Next, modules were assigned arbitrary but unique color labels (e.g. turquoise, blue, and yellow) to facilitate visualization and discussion. For each module, module eigengenes (first principal component) were computed and subsequently averaged within each diagnostic subgroup. Module stability was evaluated using the median and interquartile range (IQR) of module eigengenes across diagnostic subgroups and tested with the Kruskal–Wallis rank sum test followed by Dunn's post hoc test. Module similarity between subgroups was assessed using Pearson and Spearman correlation coefficients.

### Data Accessibility

Additional experimental data are provided in the Supplementary Materials. Unprocessed data are available in the Mendeley Data Repository (https://data.mendeley.com/datasets/yzsktrxh6y/1; DOI:10.17632/yzsktrxh6y.1).

## Results

### Cohort Characteristics and Descriptive Data

The study group comprised 93 patients with KTCN (19 female patients and 74 male patients), 10 patients with PLVC (3 female patients and 7 male patients), 4 patients with PMD (4 male patients), and 44 non-ectatic control individuals (24 female patients and 20 male patients), recruited according to the approved bioethics protocols. Clinical numerical variables, including age, thinnest corneal thickness (TCT), and posterior elevation, are summarized in Table 1. Patients with PLVC and PMD were older than the KTCN subgroup (adjusted *P* < 0.05, Dunn’s post hoc test). The corneas of KTCN and PLVC patients were thinner (lower TCT values) compared with those of patients with PMD and non-ectatic control individuals (adjusted *P* < 0.05 and adjusted *P* < 0.001, respectively; Dunn’s post hoc test). The values of flat keratometry (K1) and steep keratometry (K2) were higher in the patients with KTCN in comparison to non-ectatic controls and patients with PLVC (adjusted *P* < 0.001, Dunn's post hoc test), whereas the values of maximal corneal curvature (Kmax) differed only in the comparison of patients with KTCN and non-ectatic controls (adjusted *P* < 0.001, Dunn's post hoc test). Both the anterior and posterior elevations were the parameters discriminating patients with corneal ectasia, KTCN, PLVC, and PMD, from non-ectatic controls (adjusted *P* < 0.001, adjusted *P* < 0.05, and adjusted *P* < 0.001, respectively; Dunn's post hoc test). The highest posterior elevation values were observed in patients with PMD (adjusted *P* < 0.05, Dunn’s test).

**Table 1.** Characteristics of the Examined Non-Ectatic Control Individuals and Patients With KTCN, PLVC, and PMD Derived on Numerical Clinical Data

	Non-Ectatic Control		KTCN		PLVC		PMD
	Females *n* = 24	Males *n* = 20	Females *n* = 19	Males *n* = 74	Females *n* = 3	Males *n* = 7	Males *n* = 4
Age							
Mean *±* SD	33.5 ± 9.9	30.4 ± 8.9	31.8 *±* 15.4	31.3 *±* 12.5	41.7 *±* 10.1	38.1 *±* 8.7	49.3 *±* 11.9
Median	30	30	29	28	43	39	50
TCT, µm							
Mean *±* SD	540.0 ± 34.6	533.8 ± 35.2	430.9 *±* 45.5	429.3 *±* 65.0	469.3 *±* 64.0	405.7 *±* 57.3	513.5 *±* 29.8
Median	546	531	442	442	456	379	521
K1, D							
Mean *±* SD	43.7 ± 1.3	42.5 ± 1.4	44.7 ± 4.4	49.7 ± 8.2	42.3 *±* 4.1	41.8 *±* 4.0	42.4 *±* 3.8
Median	43.9	42.5	46.4	46.8	43.1	41.1	42.5
K2, D							
Mean *±* SD	44.7 ± 1.5	43.4 ± 1.6	51.5 ± 5.0	53.3 ± 9.1	43.9 *±* 4.5	44.4 *±* 6.2	48.0 *±* 2.4
Median	44.5	43.1	50.9	51.1	43.9	42.8	47.3
Kmax, D							
Mean *±* SD	45.1 ± 1.6	43.7 ± 1.7	58.3 ± 6.9	61.5 ± 11.2	47.2 *±* 5.6	51.0 *±* 7.2	51.8 *±* 4.5
Median	45.0	43.4	58.9	58.0	45.6	48.0	50.7
Anterior elevation, µm							
Mean *±* SD	3.0 ± 2.0	3.0 ± 2.0	32.8 ± 14.6	38.1 ± 21.6	10.0 *±* 3.6	28.3 *±* 23.9	58.8 *±* 40.2
Median	3.2	3.5	34.0	33.0	11.0	18.0	44.0
Posterior elevation, µm							
Mean *±* SD	5.1 ± 5.4	7.4 ± 5.8	73.2 *±* 27.8	76.9 *±* 38.1	29.7 *±* 8.1	47.7 *±* 29.8	97.5 *±* 30.0
Median	4	6	78	70	31	36	87.5

K1, flat keratometry; K2, steep keratometry; Kmax, maximal corneal curvature; SD, standard deviation; TCT, thinnest corneal thickness.

TCT values were missing for one non-ectatic control and 3 patients with KTCN; K1 values were missing for 6 patients with KTCN, K2 values were missing for 6 patients with KTCN, Kmax values were missing for 7 patients with KTCN, anterior elevation values were missing for 10 patients with KTCN, whereas posterior elevation values were not available for 11 patients with KTCN.

For statistical analyses, ophthalmological data from one eye per individual was used. For the patients with corneal ectasia, the eyes with more advanced disease were selected.

Data for clinical nominal variables (including sex, allergy, atopy and/or asthma, smoking, eye rubbing, intense eye rubbing, and dust in the working environment), stratified by diagnosis, are presented in Table 2. The descriptive analysis demonstrates the expected sex imbalance in the KTCN and PMD subgroups, namely the predominant male representation (*P* < 0.001 and *P* < 0.05, respectively, Chi-square test). Moreover, the (i) eye rubbing (*P* < 0.01, Chi-square test), (ii) intense eye rubbing (*P* < 0.005, Chi-square test), and (iii) exposure to dust in the working environment (defined as an exposure to filings of metals/ building materials/construction materials or excessive dust) were more frequently reported by patients with KTCN than non-ectatic controls (*P* < 0.05, Chi-square test). The patients with PLVC did not differ from non-ectatic controls in frequencies of eye rubbing, intense eye rubbing, or dust exposure. No statistically significant differences were observed among study subgroups in the proportion of patients with diagnosed allergy or atopy and/or asthma. Individual clinical datasets are presented in Supplementary Tables S1 and S2, whereas the results of statistical comparisons are detailed in Supplementary Table S3.

**Table 2.** Characteristics of the Examined Non-Ectatic Control Individuals and Patients With KTCN, PLVC, and PMD Derived on Nominal Clinical Data

	Non-Ectatic Control, *n* (%)	KTCN, *n* (%)	PLVC, *n* (%)	PMD, *n* (%)
Sex				
Female	24 (54.55)	19 (20.43)	3 (30.00)	0 (0.00)
Male	20 (45.45)	74 (79.57)	7 (70.00)	4 (100.00)
Severe KTCN				
Yes	0 (0.00)	54 (58.06)	0 (0.00)	0 (0.00)
No	0 (0.00)	38 (40.86)	0 (0.00)	0 (0.00)
N/A	44 (100.00)	1 (1.08)	10 (100.00)	4 (100.00)
Allergy				
Yes	12 (27.27)	23 (24.73)	2 (20.00)	0 (0.00)
No	32 (72.73)	68 (73.12)	8 (80.00)	4 (100.00)
N/D	0 (0.00)	2 (2.15)	0 (0.00)	0 (0.00)
Atopy and/or asthma^*^				
Yes	4 (9.09)	7 (7.53)	1 (10.00)	0 (0.00)
No	40 (90.91)	84 (90.32)	9 (90.00)	4 (100.00)
N/D	0 (0.00)	2 (2.15)	0 (0.00)	0 (0.00)
Smoking				
Yes	8 (18.18)	21 (22.58)	3 (30.00)	1 (25.00)
No	36 (81.82)	66 (70.97)	7 (70.00)	3 (75.00)
N/D	0 (0.00)	6 (6.45)	0 (0.00)	0 (0.00)
Eye rubbing				
Yes	31 (70.45)	82 (88.17)	7 (70.00)	4 (100.00)
No	13 (29.55)	10 (10.75)	3 (30.00)	0 (0.00)
N/D	0 (0.00)	1 (1.08)	0 (0.00)	0 (0.00)
Intense eye rubbing				
Yes	7 (15.91)	32 (34.41)	2 (20.00)	2 (50.00)
No	35 (79.55)	44 (47.31)	5 (50.00)	2 (50.00)
N/D	2 (4.55)	17 (18.28)	3 (30.00)	0 (0.00)
Dust in the working environment				
Yes	5 (11.36)	27 (29.03)	1 (10.00)	0 (0.00)
No	39 (88.64)	63 (67.74)	9 (90.00)	4 (100.00)
N/D	0 (0.00)	3 (3.23)	0 (0.00)	0 (0.00)

N/A, not applicable; N/D, no data available.

* Clinical categories “atopy” and “asthma” were merged into a single variable for statistical analyses.

### Global Structure of Serum Spectra: PCA and Covariate Effects

To address study questions, MALDI-TOF/TOF MS/MS profiling of 151 serum samples was performed, and, in total, 135 different m/z peaks (protein/peptide fragments) were detected. After TIC normalization and imputation of missing values, 133 m/z peaks were retained for downstream statistical analyses. Following integration with clinical data, the dimensionality reduction of generated metadata via PCA was performed to characterize the global m/z peaks profile and to assess whether principal axes relate to the clinical covariates of interest (age, sex, allergy, and intense eye rubbing). A complete list of detected and identified m/z peaks is provided in Supplementary Table S4, whereas descriptive statistics (means, medians, and standard deviations) of m/z peak intensities stratified by diagnosis are presented in Supplementary Table S5.

**Figure 1.**
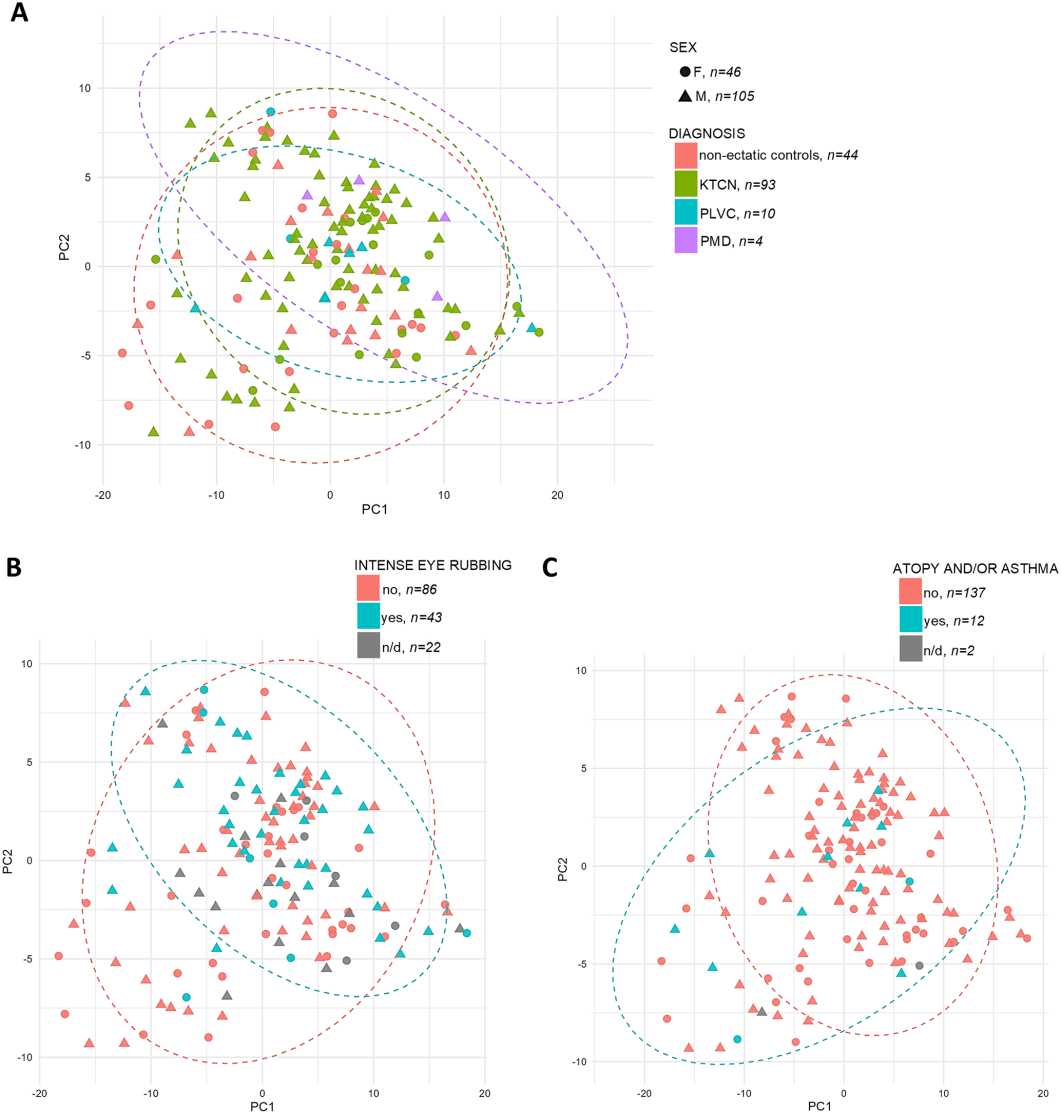
Principal component analysis (PCA) plot of serum proteomic data illustrating sample clustering patterns. (**A**) Stratification by diagnosis. Patient subgroups are color-coded: non-ectatic controls are marked in *pink*, keratoconus (KTCN) in *green*, post-laser vision correction (post-LVC) ectasia in *blue*, and violet pellucid marginal degeneration (PMD) in *violet*. Ellipses representing 95% confidence intervals (calculated using the t-distribution) were generated based on diagnosis status. (**B**) Stratification by intense eye-rubbing behavior. Subgroups are color-coded: *blue* for individuals who reported intensive eye rubbing (“yes”) and *pink* for those who did not (“no”). Ellipses representing 95% confidence intervals were generated based on eye-rubbing categories. (**C**) Stratification by atopy and/or asthma status. Subgroups are color-coded: *blue* for individuals who reported atopy or asthma (“yes”) and *pink* for those who did not (“no”). Ellipses representing 95% confidence intervals were generated based on atopy and/or asthma category. For all plots, patient sex is indicated by point shape: *circles* denote female individuals and *triangles* denote male individuals.

In the PCA, the first two principal components (PC1 and PC2) captured a meaningful portion of inter-sample variance (40.32% and 13.93% of variance, respectively) and were therefore used as low-dimensional descriptors. PCA plots with samples color-coded by diagnosis and shaped by sex are presented in Figure 1. To quantify the PCA plot observations on the differences in samples separation, different linear models specifying PC1 and PC2 as outcome variables, and age, sex, diagnosis, allergy, intense eye rubbing, posterior elevation, TCT, atopy and/or asthma, smoking, and dust in the working environment as predictors, were evaluated (Supplementary Table S6). In the fitted PC1 model (Table 3A), a statistically significant and strong negative association was found for declared atopy and/or asthma. Age also contributed to PC1 scores, pointing to the increasing age relating to lower serum peptide principal component values. In contrast, PMD status was linked with a positive shift in PC1 after an adjustment for clinical and behavioral covariates. Both a KTCN status and a history of intense eye rubbing showed positive trends, suggesting their potential contributions to PC1 variation. Similarly, the fitted PC2 model identified the KTCN status, alongside male sex, as a contributor to variance (Table 3B), reinforcing the hypothesis that local ocular disease is reflected in the serum proteomic signal.

**Table 3.** Results of Linear Modeling of PC1 (a) and PC2 (b) Against Covariates of Age, Sex, Diagnosis, Intense Eye Rubbing, and Atopy and/or Asthma (*n* = 128)

	Estimate	Std. Error	Statistic	*P* Value	Conf. Low	Conf. High	Std. Beta
**A**							
(Intercept)	2.8997	2.1652	1.3392	0.1830	−1.3872	7.1866	NA
Age	−0.1361	0.0524	−2.5994	**0.0105**	−0.2399	−0.0324	−0.2299
Male sex	−0.7004	1.4923	−0.4694	0.6397	−3.6550	2.2542	−0.0438
Diagnosis of KTCN	1.8890	1.4980	1.2610	0.2098	−1.0770	4.8551	0.1256
Diagnosis of PLVC	−0.1747	2.9659	−0.0589	0.9531	−6.0470	5.6977	−0.0054
Diagnosis of PMD	8.3288	3.9513	2.1079	**0.0371**	0.5055	16.1521	0.1957
Intense eye rubbing	2.3635	1.4062	1.6807	0.0954	−0.4208	5.1478	0.1507
Atopy and/or asthma	−5.5151	2.5173	−2.1909	**0.0304**	−10.4991	−0.5311	−0.1904
**B**							
(Intercept)	−1.2027	1.3098	−0.9183	0.3603	−3.7961	1.3906	NA
Age	−0.0208	0.0317	−0.6557	0.5133	−0.0835	0.0420	−0.0592
Male sex	1.4273	0.9027	1.5811	0.1165	−0.3601	3.2146	0.1508
Diagnosis of KTCN	1.2065	0.9062	1.3313	0.1856	−0.5878	3.0007	0.1354
Diagnosis of PLVC	1.9788	1.7942	1.1029	0.2723	−1.5736	5.5312	0.1025
Diagnosis of PMD	2.6801	2.3903	1.1212	0.2644	−2.0525	7.4127	0.1063
Intense eye rubbing	1.1109	0.8507	1.3058	0.1941	−0.5735	2.7952	0.1196
Atopy and/or asthma	−1.7272	1.5228	−1.1343	0.2590	−4.7422	1.2878	−0.1006

The *P* values in bold represent statistical significance.

### Per-Feature Testing: Evidence Implicating Diagnosis, Intense Eye Rubbing, and Atopy and/or Asthma

Per-feature testing was performed to verify the associations among disease status, selected clinical variables, and the serum proteomic profiles.

**Figure 2.**
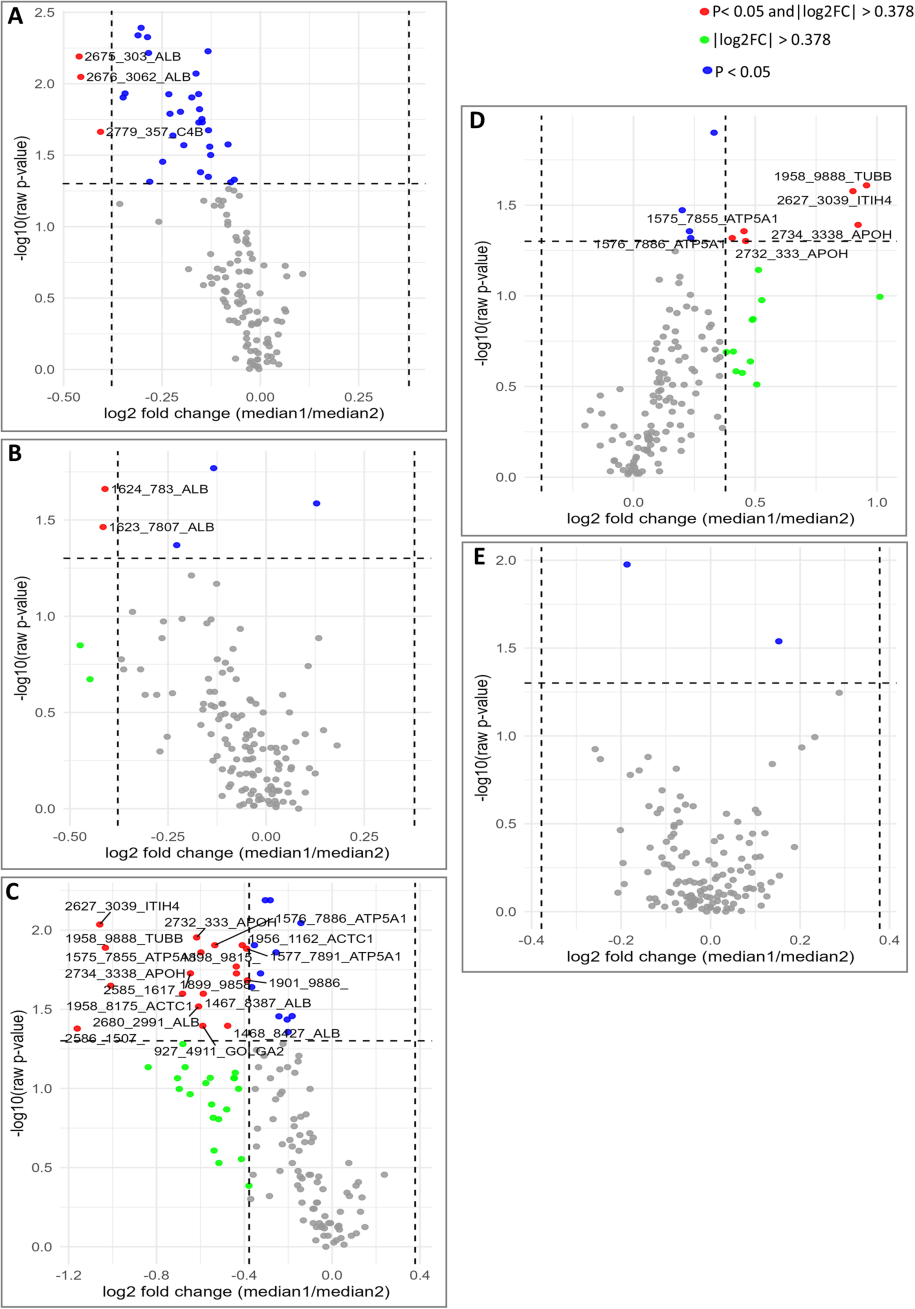
Volcano plots of differential m/z peaks of serum proteomic data. (**A**) Comparison between patients with keratoconus (KTCN, *n* = 93) and non-ectatic control individuals (*n* = 44). (**B**) Comparison between patients with post-laser vision correction (post-LVC, *n* = 10) ectasia and non-ectatic control individuals (*n* = 44). (**C**) Comparison between patients with pellucid marginal degeneration (PMD, *n* = 4) and non-ectatic control individuals (*n* = 44). (**D**) Comparison between patients with KTCN (*n* = 93) and patients with PMD (*n* = 4). (**E**) Comparison between patients with KTCN (*n* = 93) and patients with post-LVC ectasia (*n* = 10). For each comparison, univariate analyses were performed using Mann–Whitney *U* (Wilcoxon rank-sum) tests, and log_2_ fold-change (log_2_FC) values were calculated based on subgroup medians. The *green dots* represent specific m/z peaks fitting only the criterion of |log_2_FC| > 0.3785 (corresponding to ≥1.3-fold increases or ≤1/1.3-fold decreases), *blue dots* denote specific m/z peaks fitting only the criterion of *P* value < 0.05, whereas *red dots* indicate specific m/z peaks fitting both criteria. See Supplementary Table S7 for more details.

When comparing the diagnosed disease subgroups, in the KTCN versus non-ectatic controls comparison, the most depleted were two fragments of the ALB protein (2675.303 and 2676.3062) and one fragment of C4B (2779.357), as visualized in Figure 2A (detailed data in Supplementary Table S7). In the PLVC versus non-ectatic controls comparison, two other fragments of ALB were found to be downregulated (1623.7807 and 1624.783; Fig. 2B). However, compared with non-ectatic controls, the most distinct serum profiles were observed in patients with PMD, showing 18 differentiating m/z peaks (Fig. 2C). These peaks included fragments assigned to ATP5A1 (1575.7855, 1576.7886, and 1577.7891), ALB (1467.8387, 1468.8427, and 2680.2991), ACTC1 (1956.0012 and 1958.0082), APOH (2732.333 and 2734.3338), ITIH4 (2627.3038), TUBB (1958.9888), and GOLGA2 (927.4911). Next, in the comparison between KTCN and PMD, fragments of ATP5A1 (1576.7886 and 1575.7855), APOH (2732.333 and 2734.3338), ITIH4 (2627.3039), and TUBB (1958.9888) were identified as enriched (Fig. 2D). No statistically significant differences were detected between KTCN and PLVC (Fig. 2E).

**Figure 3.**
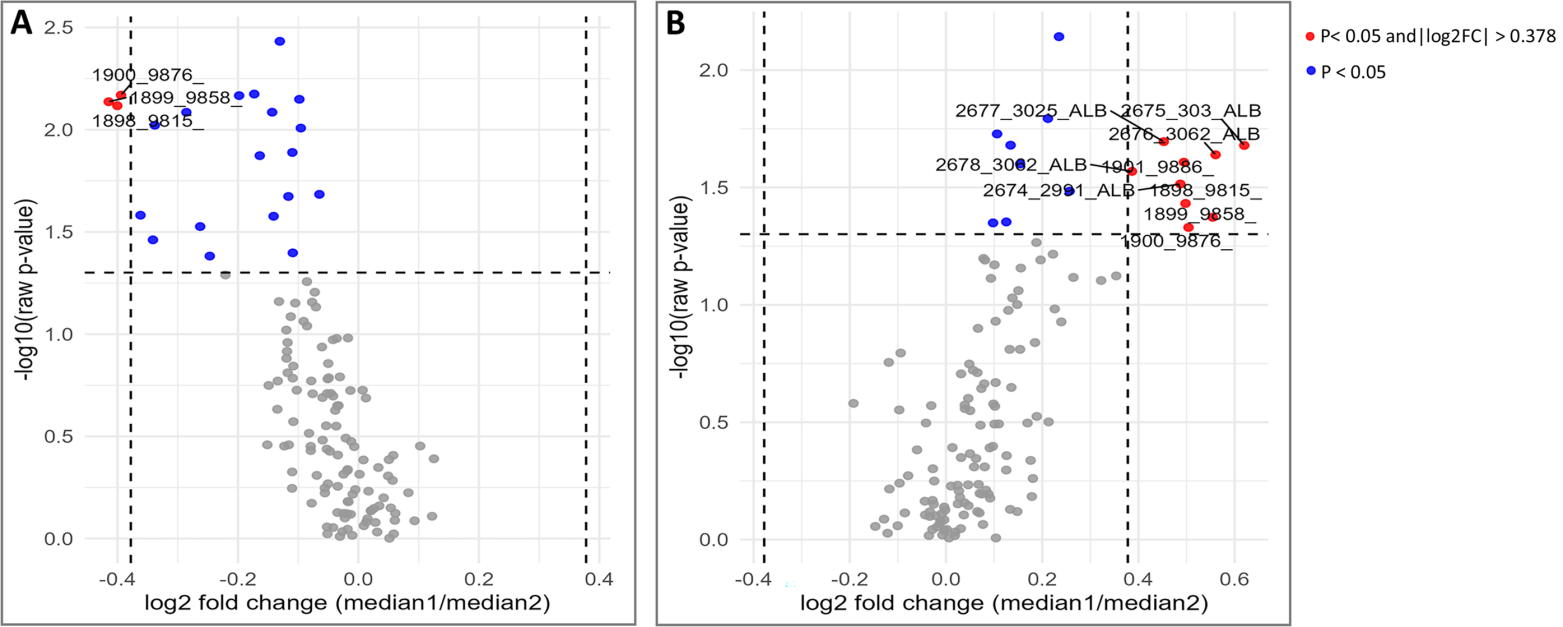
Volcano plots of differential m/z peaks of serum proteomic data. (**A**) Stratification by intense eye-rubbing behavior (“yes,” *n* = 43 and “no,” *n* = 86). (**B**) Stratification by atopy and/or asthma status (“yes,” *n* = 12 and “no,” *n* = 137). For each comparison, univariate analyses were performed using Mann–Whitney *U* (Wilcoxon rank-sum) tests, and log_2_ fold-change (log_2_FC) values were calculated based on subgroup medians. The *red dots* represent specific m/z peaks fitting the criteria of |log_2_FC| > 0.3785 (corresponding to ≥1.3-fold increases or ≤1/1.3-fold decreases) and *P* value < 0.05, whereas the *blue dots* denote specific m/z peaks fitting only the criterion of *P* value < 0.05. See Supplementary Table S7 for more details.

To investigate the potential effect of intense eye rubbing (based on the patients’ answers in questionnaires) across all study subgroups, Mann–Whitney *U* test was applied to each m/z feature (Fig. 3A, see Supplementary Table S7). The strongest rubbing-associated negative signals corresponded to three unidentified m/z peaks (1899.9858, 1898.8152, and 1900.9876), and to several ALB-assigned peaks (e.g. 2675.303, 2674.2991, and 2676.3062).

In the analysis for atopy and/or asthma, the intensities of five m/z peaks assigned to ALB (2678.3062, 2677.3025, 2674.2991, 2676.3062, and 2675.303), and four unidentified m/z peaks (1901.9886, 1898.8152, 1900.9876, and 1899.9858) were identified as upregulated in the serum of patients with diagnosed atopy and/or asthma (Fig. 3B, see Supplementary Table S7).

Further comparisons involving sex, disease severity, allergy status, smoking, and occupational dust exposure are presented in Supplementary Figure S1. No statistically significant differences were observed for these variables.

### Correlations of m/z Peaks Intensities With Clinical Features

Correlations between continuous clinical data (including TCT and posterior elevation) with each m/z feature were computed to address whether the intensity of individual serum peaks correlated with patients’ tomography outcomes. The negative correlation between posterior elevation values and a peak assigned to ATP5B (m/z 1921.9655; Spearman ρ = −0.31, *P* < 0.0001, adjusted *P* = 0.016) and negative correlation between anterior elevation values and a peak assigned to ATP5B (m/z 1921.9655; Spearman ρ = −0.30, *P* = 0.0003, adjusted *P* = 0.033; Supplementary Fig. S2, Supplementary Table S8) were the strongest identified clinical relations. No statistically significant correlations among TCT, K1, K2, Kmax, and particular serum m/z peaks were detected after multiple testing adjustment (see Supplementary Fig. S2; Supplementary Table S8).

### Correlations of m/z Peaks Intensities With Epithelial Gene Expression Data

Proteomic data integration with corneal epithelial transcriptomics data, available for a subset of 31 samples, was performed via correlation of annotated m/z peaks with matched gene expression values, thereby assessing a hypothesis about the link between circulating peptides/proteins and the locally occurring disease. Several positive correlations of a moderate magnitude were identified; including a relation of APOH-assigned serum peaks (e.g., m/z 2732.333) with epithelial *APOH* expression (Spearman ρ = 0.43, *P* = 0.017, adjusted *P* > 0.5), and associations of multiple ALB-assigned peaks with *ALB* transcript levels in epithelium (ρ = 0.35–0.42, *P* < 0.05, adjusted *P* > 0.5), as visualized in Supplementary Figure S3 (detailed data are presented in Supplementary Table S9).

### Shared Features in Serum Proteomic Profiles Across Corneal Ectasia Subgroups

**Figure 4.**
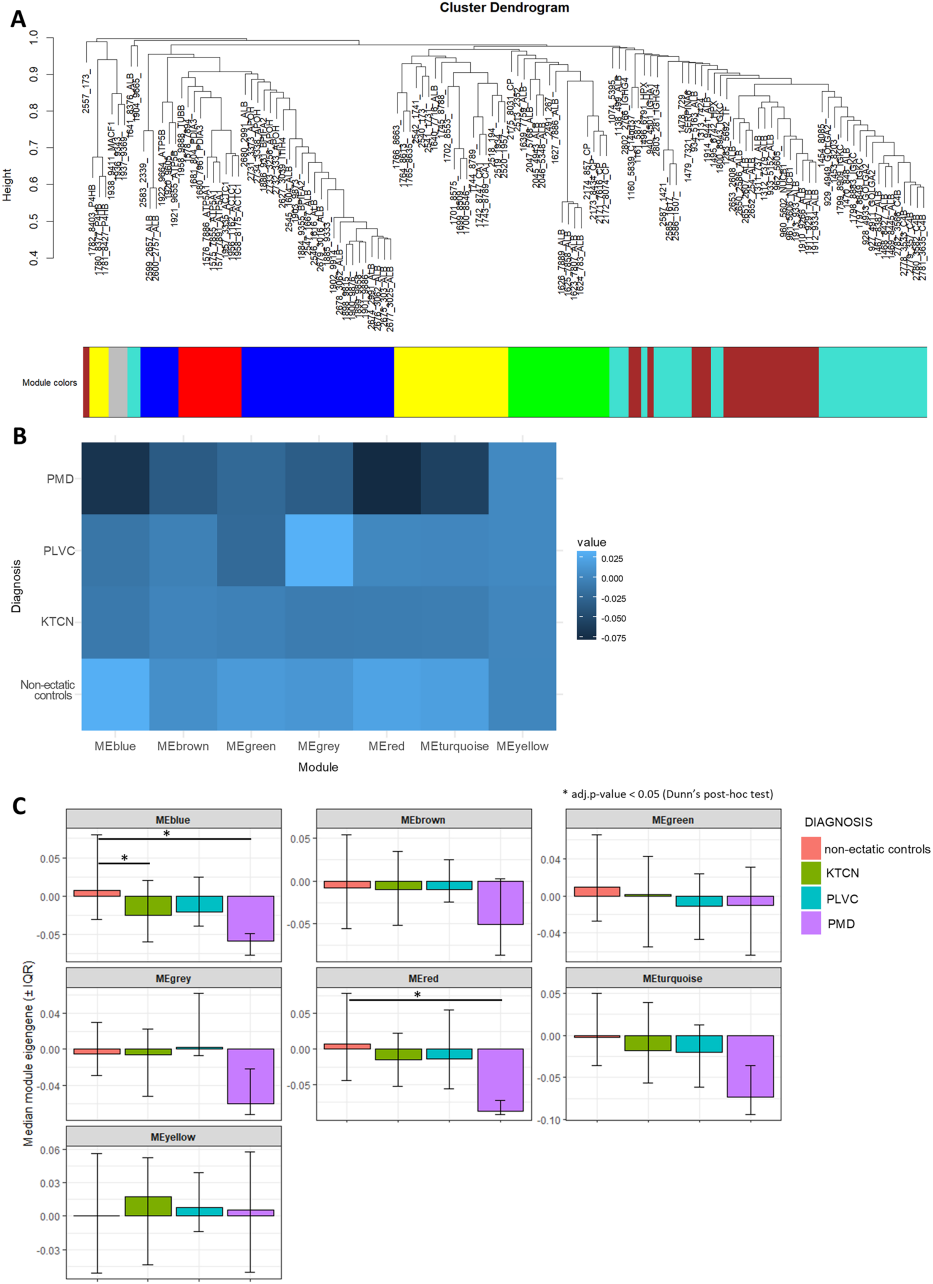
Visual representation of the results of WGCNA analysis in the study subgroups. (**A**) Hierarchical clustering of m/z peaks detected in serum samples. The dendrogram illustrates the similarity in the intensity patterns of m/z peaks across all the samples. Colored below the dendrogram indicates module assignment determined by WGCNA, with each color representing a distinct module (cluster) of co-expressed/co-present m/z peaks. (**B**) Heatmap showing the mean eigengene values for each module within each diagnostic subgroup. Rows correspond to diagnostic subgroups and the columns to modules. Each cell shows the average value of the module eigengene (i.e. the first principal component summarizing the expression of all m/z peaks within that module) calculated across all samples belonging to that subgroup, reflecting the dominant pattern of each module within each subgroup. (**C**) Bar plots of the module eigengenes per diagnostic subgroup showing the median, with the error bars extending from the first quartile (Q1) to the third quartile (Q3), representing the interquartile range (IQR).

To identify serum proteomic features shared across study subgroups, WGCNA was performed on m/z peak intensities. The dendrogram illustrating relationships between individual m/z peaks, with branch colors indicating their module assignment, is presented in Figure 4A. Seven modules, each representing a group of co-expressed/co-present m/z peaks, were recognized (Supplementary Table S10; Fig. 4B). Among these, the yellow module, which embraced 21 m/z peaks, including protein fragments annotated to CA1 and P4HB proteins (m/z peaks of 1742.8747, 1743.8789, 1780.8377, 1781.8427, and 1782.8403), was characterized by the highest stability, defined as the absence of statistically significant differences across diagnostic subgroups (*P* = 0.9673, Kruskal-Wallis rank sum test; *P* = 1.0000 for all post hoc comparisons in Dunn's test; see Figs. 4B, 4C).

Finally, pairwise comparisons of module eigengenes indicated a positive correlation between KTCN and PMD (Pearson *r* = 0.7762, Pearson *P* = 0.0402). No statistically significant correlation was observed between KTCN and PLVC (Pearson *r* = −0.1624, Pearson *P* = 0.7280). Whereas KTCN and non-ectatic controls showed statistically significant negative correlation (Pearson *r* = −0.8934, *P* = 0.0067; see Supplementary Table S10).

## Discussion

Serum proteomics provides the opportunity to assess the systemic biochemical states, including inflammatory elements, that might accompany or reflect locally occurring pathology, including that in the eye. In the context of corneal ectasias, systemic markers might arise either from cornea-derived peptides released into the circulation or from parallel systemic responses (for example, low-grade inflammation or altered protein turnover) that influence the local advancement of the disease. Our study addresses the hypothesis that systemic/circulating peptides/proteins, as those profiled by MALDI-TOF/TOF MS/MS, capture elements of both the pathophysiology of corneal ectasias and the key risk factors, such as allergy or intense eye rubbing.

Our comprehensive study demonstrated that atopy and/or asthma, age, and a PMD disease status contributed to the variability observed in serum proteomic profiles. Additionally, trends associated with the KTCN status and a history of intense eye rubbing were noticeable in the PCA assessment and were subsequently confirmed by the following univariate analyses (Mann-Whitney *U* tests), which were designed to examine the effects of a single isolated variable.

Regarding asthma and atopy in patients with KTCN, these conditions have consistently been reported as more prevalent in KTCN than in the general population.^48^^–^^50^ In some studies, even broader systemic associations with hypersensitivity-related pathways in individuals with KTCN have been suggested.^51^ In contrast, the role of IgE-mediated hypersensitivity in PMD remains poorly defined, mostly due to the lack of sufficiently large and well-characterized PMD cohorts. Interestingly, despite the broadly documented epidemiologic co-occurrence of allergy and KTCN,^52^^,^^53^ our findings did not confirm a direct effect of allergy itself on serum protein expression. Instead, the data underscored the impact of intense eye rubbing. These observations align with our previous transcriptomic findings, in which eye rubbing, but not allergy-driven, emerged as a key clinical covariate influencing the transcriptomic profiles of the KTCN cornea.^35^ Therefore, together these results suggest that with systemic hypersensitivity (e.g., atopy or atopic/allergic asthma),^12^^,^^54^ the external mechanical factor, the intense eye rubbing, which co-occurs with or results from increased ocular discomfort, might be linked to systemic proteomic alterations.

Numerous clinical and epidemiological studies have implicated eye rubbing, particularly a frequent or vigorous rubbing, as a significant risk or exacerbating factor in the development and progression of KTCN.^55^^,^^56^ Mechanistically, repeated mechanical trauma is hypothesized to induce keratocyte damage, release of inflammatory mediators, IOP fluctuations, and subsequent biomechanical weakening of the corneal stroma.^13^^,^^57^^–^^59^ Some authors have even posited that without rubbing, the disease may not fully manifest, underscoring the causal importance of this behavior in genetically predisposed individuals.^60^ In PMD, the role of eye rubbing is less well defined. Although the etiology of PMD remains unclear, some sources recommend avoiding frequent eye rubbing in affected individuals, as mechanical stress may contribute to progression. However, a recent update on PMD suggests that many patients present without a history of atopy or habitual eye rubbing, indicating that rubbing may not be a universal factor in PMD.^22^ Regarding PLVC, clinical management guidelines after refractive surgery recommend patient education to avoid rubbing. A case report even describes unilateral post-LASIK ectasia in a patient with no topographic abnormalities preoperatively but a history of intense rubbing in the affected eye, reinforcing the possible causal role of this behavior. Taken together, the literature supports that intense eye rubbing contributes to biomechanical destabilization across a spectrum of ectatic corneal disorders. Here, the intense eye rubbing was associated with a reproducible serum proteomic fingerprint, comprising the multiple unidentified peptides/protein fragments (peaks in the approximately 1898–1900 m/z region) and several albumin-linked fragments. The agreement of rubbing-signature within both unsupervised (PCA) variation and supervised per-feature comparisons strengthens the conclusion that the observed serum pattern is unlikely to be a random finding, although the interpretation remains limited by potential confounding factors and reverse causation.

Moreover, correlations between ophthalmic measurements/parameters (values of posterior and anterior elevation) of study individuals and particular m/z peaks, fragments of albumin (ALB), ATP synthase beta subunit (ATP5B), and actin isoforms (ACTC1), were recognized. The ALB signals could reflect altered vascular permeability or proteolytic processing,^61^ whereas ATP5B and ACTC1 implicate mitochondrial^62^ and cytoskeletal processes,^63^ respectively, and both have been linked to corneal remodeling and KTCN pathogenesis in prior studies.

ATP5B, a subunit of mitochondrial ATP synthase, has been implicated in oxidative stress regulation and ECM turnover. Dysregulated mitochondrial activity, including decreased ATP synthase function and increased oxidative stress, has been reported in KTCN studies. Keratocyte mitochondrial dysfunction is associated with enhanced production of reactive oxygen species (ROS), apoptosis, impaired collagen cross-linking, and degradation of stromal ECM components, all may cause weakening of the biomechanical integrity of the corneal stroma.^64^^–^^68^ The identified statistically significant inverse correlations of weak magnitude between serum ATP5B levels and both anterior and posterior corneal elevation parameters of individuals included in the study, may therefore reflect a systemic or locally driven mitochondrial dysregulation that is directly/indirectly linked to corneal biomechanical vulnerability. Although the observed effect sizes were modest (Spearman *r* ≈ −0.3), the consistency across both elevation metrics suggests a potentially biologically relevant observation.

In our study, no meaningful associations of m/z peak intensities with keratometric indices (K1, K2, and Kmax) or TCT were observed. Importantly, elevation-based parameters are considered early and sensitive indicators of corneal biomechanical weakening in KTCN, as they describe deviations of the corneal surface from an ideal reference sphere and often detect changes before alterations in keratometry or pachymetry are evident. In contrast, K1, K2, and Kmax, which reflect corneal curvature and focusing power along different meridians, are comparatively less sensitive.^69^^,^^70^ The lack of correlation with Kmax, K1, or K2 further supports the notion that ATP5B is not associated with refractive curvature changes per se, but rather with structural destabilization manifested as anterior and posterior protrusion of the corneal surface.

The correlation between serum proteomic profiles and CE gene expression revealed concordant trends for several proteins, including APOH and ALB. These outcomes suggest that changes in CE may co-occur with alterations in circulating protein levels. However, they do not necessarily indicate a direct correspondence between local gene expression and serum protein abundance. Such correlations may also arise from shared upstream regulatory mechanisms that simultaneously affect both the cornea and serum. In a recent study, we observed a strong positive correlation between the log_2_FC values of DEGs from bulk RNA-seq and the DEGs from CE clusters in spatial transcriptomic data (Pearson *r* = 0.73, *P* < 0.001). Notably, inclusion of stromal and endothelial clusters in the analysis attenuated these correlations, suggesting cell type-specific findings. Therefore, we conclude that CE transcriptional profiles can reliably reflect the overall condition of the cornea (Wysocka, Jaskiewicz, Gajecka, 2025, unpublished data, paper in review, IOVS-25-45298). Still, the unexpected correlations between CE gene expression and corresponding serum proteins, as demonstrated in a limited subset of samples (*n* = 31), require further investigation.

The WGCNA analysis of serum m/z peak intensities allowed the identification of co-expressed/co-present modules shared across the four study subgroups, highlighting common proteomic features. Further pairwise comparisons of module eigengenes enabled the observation of an opposing pattern in detected modules (negative correlation) between KTCN and non-ectactic controls, as well as concordant module-level trends (positive correlation) between KTCN and PMD. In contrast, no consistent module-level patterns were observed for KTCN and PLVC (lack of correlation). These findings imply that certain proteomic features are shared between different types of corneal ectasia, potentially reflecting overlapping pathophysiological mechanisms.

Demographic factors such as age, sex, and genetic ancestry are known contributors to variability in serum proteome. Large-scale population proteomics studies have shown that age- and sex-related effects account for a substantial proportion of inter-individual variation in circulating/systemic protein levels. In particular, aging is associated with cumulative biological and inflammatory processes that influence plasma proteins, whereas sex-related differences have been reported for proteins involved in immune function, metabolism, and hormonal regulation.^71^^–^^73^ In addition, ancestry-related biological variability has been reported, including differences in cardiometabolic markers and insulin resistance.^74^^,^^75^ Consistent with these observations, our linear regression analyses demonstrated a contribution of age to PC1 variance, and a trend toward sex-related contribution to PC2. The unequal demographic distributions across study subgroups (older patients with PLVC and PMD than patients with KTCN and non-ectatic controls, and the male predominance in KTCN and PMD) likely influenced these patterns and underscored the importance of including demographic variables in multivariable analyses. In addition, all participants were recruited from an ethnically homogeneous population of European ancestry, which reduces confounding factors but limits broader generalizability. Given evidence that ancestry-related biological variability influences KTCN prevalence and progression,^76^ future studies in more diverse populations will be beneficial to assess the robustness of our findings.

Study limitations should be taken into account during results interpretation. Well-characterized myopic individuals constituted the non-ectatic controls, as the focus was on corneal structural and biomechanical features characteristic of ectasia, rather than on refractive error itself. Due to the limited sample sizes of the PMD and PLVC cohorts, analyses involving these groups are susceptible to outlier effects. Consequently, findings related to PMD and PLVC should be interpreted as exploratory. To assess robustness, sensitivity analyses excluding PMD and PLVC samples were performed and showed no substantial changes in the main outcomes obtained in the KTCN and non-ectatic control subgroups. Although MALDI-TOF/TOF MS/MS is well suited for untargeted pilot studies because of its sensitivity and minimal sample preparation requirements, the measurements are intrinsically semi-quantitative: ion intensities are influenced by matrix crystallization, spot heterogeneity, and ionization efficiency. To mitigate these limitations, rigorous preprocessing was performed, including the removal of sparse peaks, conservative imputation for missing values, and a TIC normalization strategy. In addition, after data curation, we opted to perform downstream analyses using approaches less commonly applied to MALDI-derived proteomic data, such as WGCNA and PCA. This strategy enabled a more comprehensive and biologically informed interpretation of the data, prioritizing the detection of differentiating or shared patterns and trends rather than relying solely on single-covariate tests, which would have been suboptimal given the multifactorial background of corneal diseases.

## Conclusions

Our findings support the hypothesis that local ocular pathology in corneal ectasia is reflected in serum proteomic profiles, alongside features of systemic inflammation and intense eye-rubbing behavior. Serum MALDI-TOF MS profiling captured the proteomic signatures associated with KTCN disease severity, and particularly the posterior elevation, as well as behavioral factors such as intense eye rubbing. Notably, pairwise eigengene comparisons revealed concordant module-level trends between KTCN and PMD, suggesting, besides ophthalmological features, overlapping molecular features between these different types of ectasia.

## Supplementary Material

Supplement 1
