# Supplementary material for: Common and Distinct Features in Serum Proteomic Profiles in Keratoconus, Post-Laser Vision Correction Ectasia, and Pellucid Marginal Degeneration
Source: Invest Ophthalmol Vis Sci. 2026 Mar 11;67(3):23. doi: 10.1167/iovs.67.3.23 (PMC12988697; doi:10.1167/iovs.67.3.23)
Supplement: Supplement 1 [file iovs-67-3-23_s001.pdf]

# Common and distinct features in serum proteomic profiles in keratoconus, post-laser vision correction ectasia, and pellucid marginal degeneration

Katarzyna Jaskiewicz-Rajewicz[1*], Alicja Wysocka[1], Eliza Matuszewska-Mach[2], Natalia Rzetecka[2], Magdalena Maleszka-Kurpiel[3,4], Jakub Wozniak[5,6], Andrzej Michalski[4], Monika Udziela[7,8], Jacek P. Szaflik[7,8], Rafal Ploski[9], Malgorzata Rydzanicz[9], Jan Matysiak[2], Marzena Gajecka[1,5*]

[1] Institute of Human Genetics, Polish Academy of Sciences, Poznan, Poland

[2] Poznan University of Medical Sciences, Chair and Department of Inorganic and Analytical Chemistry, Poznan, Poland

[3] Optegra Eye Health Care Clinic in Poznan, Poland

[4] Poznan University of Medical Sciences, Chair of Ophthalmology and Optometry, Poznan, Poland

[5] Poznan University of Medical Sciences, Chair and Department of Genetics and Pharmaceutical Microbiology, Poznan, Poland

[6] Initium BioData, Kościelna Wieś, Poland

[7] Department of Ophthalmology, Medical University of Warsaw, Warsaw, Poland

[8] SPKSO University Ophthalmic Hospital in Warsaw, Warsaw, Poland

[9] Department of Medical Genetics, Medical University of Warsaw, Warsaw, Poland

*Co-corresponding Authors:

Marzena Gajecka, Ph.D., Prof., Institute of Human Genetics Polish Academy of Sciences, Strzeszynska 32, Poznan, Poland, gamar@man.poznan.pl;

Katarzyna Jaskiewicz-Rajewicz, Ph.D., Institute of Human Genetics Polish Academy of Sciences, Strzeszynska 32, Poznan, Poland, katarzyna.jaskiewicz@igcz.poznan.pl

## SUPPLEMENTARY TABLES

**Supplementary Table S1. Numerical clinical data of the examined non-ectatic control individuals and patients with KTCN, PLVC, and PMD**

| ID | Diagnosis | Age [years] | TCT [µm] | K1 [D] | K2 [D] | Kmax [D] | Anterior Elevation [µm] | Posterior Elevation [µm] |
|---|---|---|---|---|---|---|---|---|
| 106 OPT/KTCN | KTCN | 21 | 461 | 43.6 | 47.5 | 50.1 | +12 | +28 |
| 107 OPT/KTCN | KTCN | 36 | 391 | 48.6 | 52.6 | 57.6 | +33 | +70 |
| 109 OPT/KTCN | KTCN | 24 | 470 | 44.8 | 48.1 | 53.7 | +30 | +73 |
| 10 OPT/C | non-ectatic control | 22 | 551 | 43.89 | 44.91 | 45.52 | +2 | +7 |
| 10 OPT/M | non-ectatic control | 42 | 527 | 45.5 | 46.3 | 46.8 | +1 | +5 |
| 10 WUM/KTCN | KTCN | 80 | 344 | *N/D* | *N/D* | 55 | *N/D* | *N/D* |
| 110 OPT/KTCN | KTCN | 34 | 409 | 49.6 | 56.9 | 62.2 | +38 | +85 |
| 113 OPT/KTCN | KTCN | 25 | 421 | 43 | 47.2 | 55.5 | +28 | +52 |
| 114 OPT/KTCN | PMD | 34 | 474 | 46.7 | 51.3 | 58.1 | +46 | +82 |
| 115 OPT/KTCN | KTCN | 20 | 507 | 47.2 | 49.7 | 55.3 | +27 | +61 |
| 116 OPT/KTCN | KTCN | 21 | 438 | 63.5 | 70.1 | 86.5 | +85 | +185 |
| 11 OPT/C | non-ectatic control | 31 | 601 | 42.57 | 43.09 | 43.09 | +2 | +4 |
| 11 OPT/M | non-ectatic control | 23 | 592 | 43 | 44.6 | 45 | +3 | +10 |
| 11 OPT/KTCN | KTCN | 25 | 367 | 42.4 | 44.7 | 49.7 | +24 | +61 |
| 11 WUM/KTCN | KTCN | 51 | 241 | 57 | 63.2 | 65.5 | +85 | +141 |
| 121 OPT/KTCN | KTCN | 38 | 352 | 64.9 | 70.6 | 79.7 | +70 | +136 |
| 123 OPT/KTCN | KTCN | 29 | 443 | 47.7 | 53.1 | 61.2 | +46 | +85 |
| 126 OPT/KTCN | KTCN | 18 | 357 | 58.8 | 64.2 | 77.3 | +73 | +166 |
| 129 OPT/KTCN | KTCN | 23 | 430 | 55.7 | 61.2 | 71.2 | +60 | +137 |
| 12 OPT/C | non-ectatic control | 21 | 538 | 43.65 | 44.24 | 44.71 | +3 | +9 |
| 12 OPT/KTCN | KTCN | 30 | 452 | 44.8 | 47.7 | 52.4 | +11 | +31 |
| 137 OPT/KTCN | KTCN | 34 | 461 | 47 | 53.9 | 61.7 | +29 | +64 |
| 138 OPT/KTCN | KTCN | 28 | 463 | 42.2 | 46.8 | 54.7 | +30 | +44 |
| 13 OPT/C | non-ectatic control | 27 | 538 | 44.28 | 44.34 | 45.61 | +3 | +4 |
| 13 WUM/KTCN | KTCN | 87 | 404 | 58.9 | 63.7 | 66 | +45 | +111 |

| | | | | | | | | |
|---|---|---|---|---|---|---|---|---|
| 143 OPT/KTCN | KTCN | 26 | 470 | 43.6 | 47.9 | 53.8 | +22 | +46 |
| 147 OPT/KTCN | KTCN | 28 | 452 | 49.3 | 51.3 | 57.8 | +30 | +64 |
| 14 WUM/KTCN | KTCN | 39 | *N/D* | 58.9 | 61.1 | 74.8 | +60 | *N/D* |
| 150 OPT/KTCN | KTCN | 34 | 472 | 46.6 | 54.6 | 65.9 | +37 | +79 |
| 152 OPT/KTCN | KTCN | 30 | 412 | 55.7 | 60.2 | 69.5 | +46 | +95 |
| 153 OPT/KTCN | KTCN | 47 | 397 | 44.4 | 48.2 | 53.3 | +41 | +86 |
| 155 OPT/KTCN | KTCN | 32 | 391 | 48.1 | 51.6 | 59 | +42 | +82 |
| 15 OPT/C | non-ectatic control | 24 | 552 | 41.43 | 41.94 | 42.28 | +3 | +9 |
| 15 OPT/KTCN | KTCN | 22 | 519 | 40.4 | 43.4 | 54.5 | +24 | +63 |
| 161 OPT/KTCN | KTCN | 22 | 449 | 53.6 | 53.9 | 70.3 | +53 | +94 |
| 162 OPT/KTCN | KTCN | 23 | 459 | 43.7 | 45.4 | 51.9 | +22 | +47 |
| 163 OPT/KTCN | KTCN | 27 | 435 | 46.4 | 50.4 | 59.5 | +33 | +78 |
| 166 OPT/KTCN | KTCN | 23 | 411 | 53.6 | 59.8 | 71.4 | +43 | +81 |
| 168 OPT/KTCN | KTCN | 27 | 484 | 41.8 | 43 | 49.2 | +41 | +101 |
| 16 OPT/KTCN | KTCN | 28 | 530 | 41.9 | 44.1 | 50.9 | +20 | +56 |
| 16 WUM/KTCN | KTCN | 43 | *N/D* | *N/D* | *N/D* | *N/D* | *N/D* | *N/D* |
| 170 OPT/KTCN | KTCN | 29 | 523 | 44.1 | 48 | 52.1 | +25 | +65 |
| 175 OPT/KTCN | PMD | 63 | 535 | 37.6 | 47.8 | 49.6 | +118 | +141 |
| 177 OPT/KTCN | KTCN | 22 | 438 | 48.4 | 53.9 | 63.7 | +38 | +75 |
| 178 OPT/KTCN | PLVC | 45 | 451 | 41.9 | 44.9 | 47 | +14 | +36 |
| 179 OPT/KTCN | KTCN | 31 | 499 | 43 | 46.7 | 50.5 | +20 | +48 |
| 17 OPT/C | non-ectatic control | 25 | 570 | 43.54 | 44.43 | 44.81 | +2 | +4 |
| 17 OPT/KTCN | PLVC | 45 | 361 | 50.3 | 57.8 | 65.4 | +48 | +103 |
| 17 WUM/KTCN | KTCN | 21 | 283 | 70.5 | 66.35 | *N/D* | +40 | +119 |
| 185 OPT/KTCN | KTCN | 31 | 460 | 45.6 | 47.4 | 50.5 | +18 | +47 |
| 186 OPT/KTCN | KTCN | 21 | 512 | 43.4 | 44.6 | 46.6 | +7 | +21 |
| 189 OPT/KTCN | KTCN | 26 | 440 | 55.3 | 66.8 | 79.4 | +61 | +87 |
| 18 OPT/C | non-ectatic control | 28 | 541 | 44.48 | 44.63 | 44.98 | +1 | +2 |
| 193 OPT/KTCN | KTCN | 30 | 439 | 56.9 | 61.7 | 75 | +47 | +104 |
| 195 OPT/KTCN | PLVC | 31 | 413 | 37.8 | 39.5 | 42.6 | +11 | +31 |

| | | | | | | | | |
|---|---|---|---|---|---|---|---|---|
| 196 OPT/KTCN | KTCN | 30 | 431 | 46.4 | 49.1 | 68 | +54 | +97 |
| 197 OPT/KTCN | KTCN | 19 | 488 | 42.4 | 45.5 | 53.2 | +30 | +61 |
| 19 OPT/C | non-ectatic control | 30 | *N/D* | 42.76 | 42.93 | 43.17 | +3 | +6 |
| 19 OPT/KTCN | KTCN | 20 | 396 | 48.9 | 51.4 | 67 | +33 | +49 |
| 1F/2 | non-ectatic control | 48 | 466 | 46.7 | 47.5 | 47.9 | +2 | +18 |
| 1 OPT/M | non-ectatic control | 33 | 501 | 45.4 | 46.5 | 47 | +2 | -1 |
| 1 OPT/KTCN | KTCN | 24 | 443 | 43.9 | 47.4 | 55.2 | +35 | +75 |
| 1 WUM/KTCN | KTCN | 75 | 295 | *N/D* | *N/D* | *N/D* | *N/D* | *N/D* |
| 200 OPT/KTCN | PLVC | 51 | 456 | 43.1 | 43.9 | 45.6 | +6 | +21 |
| 20 OPT/KTCN | KTCN | 29 | 519 | 45.5 | 52 | 59.2 | +17 | +44 |
| 21 OPT/C | non-ectatic control | 54 | 533 | 43.24 | 44.32 | 44.52 | +4 | +5 |
| 21 OPT/KTCN | KTCN | 27 | 450 | 43.6 | 44 | 47.4 | +7 | +14 |
| 22 OPT/KTCN | KTCN | 25 | 480 | 43.1 | 47.2 | 52.5 | +19 | +42 |
| 23 OPT/C | non-ectatic control | 36 | 536 | 42.55 | 43.19 | 43.41 | +1 | +3 |
| 24 OPT/KTCN | KTCN | 39 | 419 | 50.2 | 56.3 | 63.7 | +34 | +55 |
| 25 OPT/C | non-ectatic control | 37 | 530 | 41.83 | 41.94 | 42.18 | +2 | +4 |
| 25 OPT/KTCN | KTCN | 44 | 398 | 54.9 | 58.5 | 69.3 | +45 | +89 |
| 26 OPT/C | non-ectatic control | 35 | 555 | 43.77 | 44.4 | 44.8 | +2 | 0 |
| 26 OPT/KTCN | PLVC | 43 | 539 | 45.9 | 48.4 | 53.4 | +13 | +37 |
| 27 OPT/C | non-ectatic control | 29 | 566 | 43.1 | 43.6 | 44 | +4 | 0 |
| 27 OPT/KTCN | PLVC | 46 | 501 | 38.5 | 39.2 | 46 | +22 | +34 |
| 28 OPT/C | non-ectatic control | 29 | 519 | 39.8 | 40.4 | 40 | +5 | 0 |
| 28 OPT/KTCN | KTCN | 20 | 461 | 43.8 | 50.7 | 59.9 | +29 | +52 |
| 29 OPT/C | non-ectatic control | 26 | 513 | 43.5 | 44.4 | 44 | +6 | 0 |
| 29 OPT/KTCN | KTCN | 34 | 344 | 54.2 | 57.4 | 69.3 | +37 | +88 |
| 2F/2 | KTCN | 51 | 440 | 44.3 | 48.3 | 53.4 | +24 | +42 |
| 2 OPT/M | non-ectatic control | 36 | 496 | 45.9 | 47.5 | 48.4 | +5 | +4 |
| 2 OPT/KTCN | KTCN | 25 | 361 | 51.8 | 53 | 65.9 | +34 | +107 |
| 2 WUM/KTCN | KTCN | 31 | 303 | *N/D* | *N/D* | *N/D* | *N/D* | *N/D* |
| 30 OPT/C | non-ectatic control | 29 | 556 | 43.5 | 44.1 | 44 | +5 | 0 |

| | | | | | | | | |
|---|---|---|---|---|---|---|---|---|
| 31 OPT/C | non-ectatic control | 29 | 562 | 41.9 | 42.3 | 42 | +10 | 0 |
| 31 OPT/KTCN | PLVC | 32 | 353 | 39.9 | 40.6 | 48 | +12 | +16 |
| 32 OPT/C | non-ectatic control | 32 | 549 | 42.3 | 42.5 | 43 | +7 | 0 |
| 32 OPT/KTCN | KTCN | 42 | 407 | 47.7 | 51.1 | 53.3 | +32 | +65 |
| 33 OPT/C | non-ectatic control | 48 | 589 | 44.5 | 45.5 | 45 | +1 | 0 |
| 33 OPT/KTCN | KTCN | 32 | 497 | 46.8 | 48.5 | 57.5 | +27 | +80 |
| 36 OPT/KTCN | KTCN | 32 | 373 | 62.4 | 67.5 | 97.6 | +131 | +196 |
| 37 OPT/C | non-ectatic control | 31 | 459 | 42.7 | 46 | 46.7 | +3 | +2 |
| 38 OPT/C | non-ectatic control | 44 | 570 | 44.8 | 45.4 | 45.8 | +5 | +14 |
| 39 OPT/C | non-ectatic control | 54 | 566 | 43.6 | 43.7 | 44.2 | +3 | +14 |
| 3F/1 | KTCN | 42 | 441 | 43 | 47.6 | 55 | +58 | +109 |
| 3F/7 | KTCN | 63 | 448 | 42.4 | 44 | 48.8 | +13 | +44 |
| 3 OPT/M | non-ectatic control | 29 | 533 | 44.1 | 45.2 | 45.6 | +3 | +1 |
| 3 OPT/KTCN | KTCN | 28 | 449 | 43.5 | 44.7 | 52.1 | +24 | +52 |
| 3 WUM/KTCN | KTCN | 46 | 393 | *N/D* | *N/D* | *N/D* | *N/D* | *N/D* |
| 40 OPT/C | non-ectatic control | 37 | 570 | 41.6 | 42 | 42.4 | +3 | +12 |
| 41 OPT/C | non-ectatic control | 27 | 516 | 44.2 | 45.2 | 45.4 | +2 | +15 |
| 42 OPT/C | non-ectatic control | 26 | 489 | 42 | 42.8 | 43.1 | +7 | +6 |
| 43 OPT/KTCN | KTCN | 27 | 373 | 45.6 | 47.2 | 52.3 | +19 | +49 |
| 44 OPT/C | non-ectatic control | 24 | 540 | 42.3 | 43.6 | 44 | +3 | +10 |
| 44 OPT/KTCN | KTCN | 25 | 440 | 52.9 | 53.5 | 70 | +41 | +91 |
| 45 OPT/C | non-ectatic control | 29 | 568 | 42.5 | 42.9 | 43.1 | +2 | +7 |
| 48 OPT/C | non-ectatic control | 22 | 567 | 40.1 | 40.9 | 41.3 | +5 | +15 |
| 4F/6 | non-ectatic control | 38 | 564 | 45 | 46.7 | 47 | +3 | +18 |
| 4F/7 | non-ectatic control | 62 | 547 | 42.5 | 44.3 | 45.1 | +6 | +16 |
| 4 OPT/M | non-ectatic control | 30 | 515 | 44.4 | 45.3 | 45.7 | +1 | +4 |
| 4 OPT/KTCN | KTCN | 23 | 498 | 47.4 | 49.2 | 62.3 | +35 | +72 |
| 4 WUM/KTCN | KTCN | 19 | 369 | 66.6 | 69.6 | 73.9 | +77 | +166 |
| 50 OPT/C | non-ectatic control | 31 | 510 | 43.9 | 44.8 | 45.2 | +7 | +16 |
| 51 OPT/C | non-ectatic control | 25 | 596 | 43.8 | 45.6 | 46 | +4 | +10 |

| | | | | | | | | |
|---|---|---|---|---|---|---|---|---|
| 51 OPT/KTCN | PLVC | 22 | 359 | 41.1 | 42.8 | 49.8 | +18 | +28 |
| 57 OPT/KTCN | KTCN | 20 | 467 | 46.4 | 51.8 | 60.2 | +34 | +65 |
| 5F/1 | KTCN | 21 | *N/D* | *N/D* | *N/D* | *N/D* | *N/D* | *N/D* |
| 5 OPT/M | non-ectatic control | 24 | 531 | 43.3 | 44.2 | 44.4 | +2 | +2 |
| 5 OPT/KTCN | KTCN | 27 | 366 | 45.4 | 51.7 | 61.6 | +19 | +46 |
| 5 WUM/KTCN | KTCN | 27 | 288 | 64.1 | 67.9 | 78.9 | *N/D* | *N/D* |
| 62 OPT/KTCN | PLVC | 38 | 379 | 39.2 | 41.8 | 45.4 | +10 | +46 |
| 63 OPT/KTCN | KTCN | 26 | 464 | 45.4 | 46.3 | 55.8 | +35 | +65 |
| 64 OPT/KTCN | KTCN | 23 | 364 | 50.9 | 52.8 | 61.3 | +48 | +83 |
| 66 OPT/KTCN | KTCN | 20 | 467 | 43.8 | 48.1 | 54.5 | +29 | +31 |
| 69 OPT/KTCN | KTCN | 33 | 457 | 52.2 | 55.5 | 65.7 | +48 | +77 |
| 6 OPT/M | non-ectatic control | 30 | 476 | 41.5 | 43.1 | 43.4 | +1 | +1 |
| 6 OPT/KTCN | KTCN | 28 | 474 | 43.4 | 44.6 | 46.6 | +4 | +24 |
| 6 WUM/KTCN | KTCN | 56 | 284 | 70.9 | 84.8 | *N/D* | *N/D* | *N/D* |
| 70 OPT/KTCN | KTCN | 28 | 517 | 42.9 | 46 | 49.2 | +18 | +71 |
| 71 OPT/KTCN | PMD | 50 | 507 | 41.9 | 46 | 47.9 | +29 | +74 |
| 76 OPT/KTCN | KTCN | 27 | 441 | 50.3 | 52.8 | 60.5 | +35 | +76 |
| 79 OPT/KTCN | KTCN | 18 | 454 | 51.9 | 58.4 | 70.8 | +59 | +106 |
| 7 OPT/M | non-ectatic control | 23 | 504 | 39.6 | 40.9 | 41 | +3 | +6 |
| 7 OPT/KTCN | KTCN | 21 | 438 | 50.9 | 54.6 | 73.6 | +63 | +115 |
| 7 WUM/KTCN | KTCN | 62 | 520 | 73.7 | 77.7 | 92.6 | *N/D* | *N/D* |
| 80 OPT/KTCN | KTCN | 26 | 471 | 41.7 | 44.9 | 52.2 | +32 | +71 |
| 81 OPT/KTCN | KTCN | 29 | 325 | 64.5 | 65.1 | 76 | +72 | +135 |
| 83 OPT/KTCN | KTCN | 27 | 495 | 43.3 | 46.8 | 56.7 | +51 | +101 |
| 84 OPT/KTCN | KTCN | 45 | 450 | 43.7 | 47.6 | 52.2 | +16 | +47 |
| 89 OPT/KTCN | KTCN | 35 | 487 | 44.8 | 46.9 | 54.2 | +33 | +70 |
| 8 OPT/M | non-ectatic control | 21 | 518 | 43.2 | 44 | 44.3 | +1 | +4 |
| 8 OPT/KTCN | KTCN | 18 | 407 | 43.6 | 46 | 48.6 | +18 | +37 |
| 91 OPT/KTCN | KTCN | 30 | 456 | 43.2 | 46.8 | 55.7 | +28 | +59 |
| 95 OPT/KTCN | PLVC | 39 | 436 | 41.6 | 43.9 | 55.7 | +74 | +71 |

| | | | | | | | | |
|---|---|---|---|---|---|---|---|---|
| 96 OPT/KTCN | KTCN | 31 | 488 | 45.2 | 46.4 | 55.8 | +25 | +46 |
| 98 OPT/KTCN | KTCN | 31 | 448 | 47 | 51.8 | 58.4 | +31 | +65 |
| 99 OPT/KTCN | PMD | 50 | 538 | 43.1 | 46.7 | 51.7 | +42 | +93 |
| 9 OPT/C | non-ectatic control | 31 | 482 | 43.57 | 44.72 | 44.91 | +3 | +5 |
| 9 OPT/KTCN | KTCN | 18 | 473 | 41 | 41.6 | 52.1 | +24 | +46 |
| 9 WUM/KTCN | KTCN | 34 | 364 | 50 | 53.8 | 61.9 | *N/D* | *N/D* |

Abbreviations in the Table: *N/D* – no data; KTCN – keratoconus; PLVC – post-laser vision correction ectasia; PMD – pellucid marginal degeneration; TCT – thinnest corneal thickness; K1 – flat keratometry, K2 – steep keratometry, Kmax – maximal corneal curvature.

For statistical analyses, ophthalmological data from one eye per individual were used. For the patients with corneal ectasia, the eyes with more advanced disease were selected.

**Supplementary Table S2. Nominal clinical data of the examined non-ectatic control individuals and patients with KTCN, PLVC, and PMD**

| ID | Sex | Diagnosis | Severe KTCN | Allergy | Atopy | Asthma | Atopy and/or asthma | Smoking | Eye rubbing | Intense eye rubbing | Dust in the working environment |
|---|---|---|---|---|---|---|---|---|---|---|---|
| 106 OPT/KTCN | F | KTCN | no | yes | no | no | no | no | yes | no | yes |
| 107 OPT/KTCN | M | KTCN | yes | no | no | no | no | no | yes | yes | yes |
| 109 OPT/KTCN | M | KTCN | no | yes | no | no | no | yes | yes | yes | no |
| 10 OPT/C | F | Non-ectatic control | *N/A* | no | no | no | no | no | no | no | no |
| 10 OPT/M | F | Non-ectatic control | *N/A* | no | no | no | no | no | no | no | no |
| 10 WUM/KTCN | M | KTCN | yes | no | no | no | no | no | no | no | no |
| 110 OPT/KTCN | F | KTCN | no | no | no | no | no | no | yes | no | no |
| 113 OPT/KTCN | M | KTCN | no | yes | no | no | no | No | yes | no | no |
| 114 OPT/KTCN | M | PMD | *N/A* | no | no | no | no | no | yes | no | no |
| 115 OPT/KTCN | M | KTCN | no | no | no | no | no | yes | yes | no | no |
| 116 OPT/KTCN | M | KTCN | yes | no | no | no | no | no | yes | yes | no |
| 11 OPT/C | M | Non-ectatic control | *N/A* | no | no | no | no | no | yes | no | no |
| 11 OPT/M | F | Non-ectatic control | *N/A* | no | no | no | no | no | no | no | no |
| 11 OPT/KTCN | M | KTCN | no | no | no | no | no | no | yes | no | yes |
| 11 WUM/KTCN | M | KTCN | yes | no | no | yes | yes | no | no | no | no |
| 121 OPT/KTCN | M | KTCN | yes | no | no | no | no | yes | yes | no | yes |
| 123 OPT/KTCN | F | KTCN | yes | no | no | no | no | no | yes | no | no |
| 126 OPT/KTCN | M | KTCN | yes | no | no | no | no | no | yes | no | no |
| 129 OPT/KTCN | M | KTCN | yes | no | no | no | no | no | yes | no | yes |
| 12 OPT/C | F | Non-ectatic control | *N/A* | no | no | no | no | no | yes | no | no |
| 12 OPT/KTCN | F | KTCN | no | no | no | no | no | no | no | no | no |
| 137 OPT/KTCN | M | KTCN | no | no | no | no | no | no | yes | no | yes |
| 138 OPT/KTCN | M | KTCN | no | yes | no | no | no | no | yes | yes | no |

| | | | | | | | | | | | |
|---|---|---|---|---|---|---|---|---|---|---|---|
| 13 OPT/C | F | Non-ectatic control | *N/A* | no | no | no | no | no | yes | no | no |
| 13 WUM/KTCN | F | KTCN | yes | yes | no | no | no | yes | yes | yes | yes |
| 143 OPT/KTCN | M | KTCN | no | yes | no | no | no | no | yes | yes | no |
| 147 OPT/KTCN | F | KTCN | no | no | no | no | no | *N/D* | yes | yes | no |
| 14 WUM/KTCN | M | KTCN | yes | no | no | no | no | yes | yes | no | no |
| 150 OPT/KTCN | M | KTCN | yes | no | no | no | no | no | yes | no | yes |
| 152 OPT/KTCN | M | KTCN | yes | no | no | no | no | yes | yes | yes | no |
| 153 OPT/KTCN | F | KTCN | yes | no | no | no | no | *N/D* | yes | no | no |
| 155 OPT/KTCN | M | KTCN | yes | no | no | no | no | yes | yes | yes | no |
| 15 OPT/C | F | Non-ectatic control | *N/A* | no | no | no | no | yes | yes | no | no |
| 15 OPT/KTCN | M | KTCN | yes | no | no | no | no | no | yes | *N/D* | yes |
| 161 OPT/KTCN | M | KTCN | yes | yes | no | no | no | no | yes | yes | no |
| 162 OPT/KTCN | M | KTCN | no | yes | no | no | no | yes | yes | no | no |
| 163 OPT/KTCN | F | KTCN | yes | no | no | no | no | *N/D* | yes | no | no |
| 166 OPT/KTCN | M | KTCN | yes | no | no | no | no | *N/D* | yes | yes | no |
| 168 OPT/KTCN | M | KTCN | no | no | no | no | no | no | yes | no | yes |
| 16 OPT/KTCN | M | KTCN | no | no | no | no | no | no | yes | *N/D* | yes |
| 16 WUM/KTCN | M | KTCN | yes | *N/D* | *N/D* | *N/D* | *N/D* | no | yes | no | *N/D* |
| 170 OPT/KTCN | M | KTCN | no | yes | yes | yes | yes | yes | yes | yes | no |
| 175 OPT/KTCN | M | PMD | *N/A* | no | no | no | no | no | yes | yes | no |
| 177 OPT/KTCN | M | KTCN | Yes | no | no | no | no | no | yes | no | yes |
| 178 OPT/KTCN | M | PLVC | *N/A* | no | no | no | no | yes | yes | yes | no |
| 179 OPT/KTCN | M | KTCN | No | no | no | no | no | no | yes | yes | yes |
| 17 OPT/C | F | Non-ectatic control | *N/A* | no | no | no | no | no | yes | no | no |
| 17 OPT/KTCN | M | PLVC | *N/A* | no | no | no | no | yes | yes | *N/D* | no |
| 17 WUM/KTCN | M | KTCN | yes | no | no | no | no | no | yes | *N/D* | no |
| 185 OPT/KTCN | F | KTCN | no | no | no | no | no | no | yes | no | no |
| 186 OPT/KTCN | M | KTCN | no | no | no | no | no | yes | yes | yes | no |

| | | | | | | | | | | | |
|---|---|---|---|---|---|---|---|---|---|---|---|
| 189 OPT/KTCN | M | KTCN | yes | no | no | no | no | *N/D* | yes | yes | yes |
| 18 OPT/C | F | Non-ectatic control | *N/A* | yes | yes | no | yes | no | yes | no | no |
| 193 OPT/KTCN | M | KTCN | Yes | yes | no | no | no | no | yes | yes | yes |
| 195 OPT/KTCN | F | PLVC | *N/A* | yes | no | no | no | no | no | no | no |
| 196 OPT/KTCN | M | KTCN | yes | no | no | no | no | no | yes | yes | yes |
| 197 OPT/KTCN | M | KTCN | yes | no | no | no | no | no | no | no | no |
| 19 OPT/C | M | Non-ectatic control | *N/A* | yes | no | yes | yes | no | yes | yes | yes |
| 19 OPT/KTCN | M | KTCN | yes | no | no | no | no | no | yes | *N/D* | no |
| 1F/2 | F | Non-ectatic control | *N/A* | no | no | no | no | no | no | no | no |
| 1 OPT/M | F | Non-ectatic control | *N/A* | no | no | no | no | no | no | no | no |
| 1 OPT/KTCN | M | KTCN | yes | no | no | no | no | no | yes | *N/D* | yes |
| 1 WUM/KTCN | M | KTCN | yes | no | no | no | no | no | no | no | no |
| 200 OPT/KTCN | F | PLVC | *N/A* | no | no | no | no | no | yes | yes | no |
| 20 OPT/KTCN | M | KTCN | no | yes | no | yes | yes | no | yes | *N/D* | no |
| 21 OPT/C | F | Non-ectatic control | *N/A* | no | no | no | no | no | yes | no | no |
| 21 OPT/KTCN | M | KTCN | No | no | no | no | no | no | yes | *N/D* | no |
| 22 OPT/KTCN | M | KTCN | No | yes | no | no | no | no | yes | no | no |
| 23 OPT/C | M | Non-ectatic control | *N/A* | no | no | no | no | no | yes | yes | no |
| 24 OPT/KTCN | M | KTCN | No | yes | no | yes | yes | no | yes | *N/D* | no |
| 25 OPT/C | M | Non-ectatic control | *N/A* | yes | no | no | no | no | no | no | no |
| 25 OPT/KTCN | M | KTCN | Yes | no | no | no | no | yes | yes | *N/D* | no |
| 26 OPT/C | F | Non-ectatic control | *N/A* | no | no | no | no | no | yes | no | no |
| 26 OPT/KTCN | F | PLVC | *N/A* | yes | no | yes | yes | no | yes | *N/D* | no |
| 27 OPT/C | F | Non-ectatic control | *N/A* | no | no | no | no | no | yes | no | no |
| 27 OPT/KTCN | M | PLVC | *N/A* | no | no | no | no | no | no | no | yes |

| | | | | | | | | | | | |
|---|---|---|---|---|---|---|---|---|---|---|---|
| 28 OPT/C | M | Non-ectatic control | *N/A* | yes | no | yes | yes | no | no | no | no |
| 28 OPT/KTCN | M | KTCN | Yes | no | no | no | no | yes | yes | *N/D* | no |
| 29 OPT/C | F | Non-ectatic control | *N/A* | no | no | no | no | no | yes | no | no |
| 29 OPT/KTCN | F | KTCN | Yes | yes | no | no | no | no | yes | *N/D* | no |
| 2F/2 | M | KTCN | No | no | no | no | no | no | yes | no | no |
| 2 OPT/M | F | Non-ectatic control | *N/A* | no | no | no | no | yes | yes | *N/D* | no |
| 2 OPT/KTCN | F | KTCN | no | no | no | no | no | no | yes | *N/D* | no |
| 2 WUM/KTCN | M | KTCN | yes | no | no | no | no | yes | yes | no | no |
| 30 OPT/C | F | Non-ectatic control | *N/A* | yes | no | no | no | no | yes | no | no |
| 31 OPT/C | F | Non-ectatic control | *N/A* | no | no | no | no | no | yes | no | no |
| 31 OPT/KTCN | M | PLVC | *N/A* | no | no | no | no | no | yes | *N/D* | no |
| 32 OPT/C | M | Non-ectatic control | *N/A* | yes | no | no | no | no | yes | no | no |
| 32 OPT/KTCN | M | KTCN | yes | yes | no | no | no | no | yes | no | no |
| 33 OPT/C | F | Non-ectatic control | *N/A* | yes | no | no | no | no | yes | no | no |
| 33 OPT/KTCN | M | KTCN | yes | yes | no | no | no | no | yes | *N/D* | no |
| 36 OPT/KTCN | M | KTCN | yes | yes | yes | no | yes | no | yes | yes | yes |
| 37 OPT/C | F | Non-ectatic control | *N/A* | no | no | no | no | no | yes | yes | no |
| 38 OPT/C | F | Non-ectatic control | *N/A* | no | no | no | no | yes | yes | no | no |
| 39 OPT/C | F | Non-ectatic control | *N/A* | no | no | no | no | no | yes | yes | no |
| 3F/1 | F | KTCN | yes | no | no | no | no | no | yes | no | no |
| 3F/7 | M | KTCN | no | no | no | no | no | no | yes | yes | no |
| 3 OPT/M | F | Non-ectatic control | *N/A* | no | no | no | no | no | no | no | no |
| 3 OPT/KTCN | M | KTCN | no | yes | no | no | no | no | yes | yes | no |
| 3 WUM/KTCN | M | KTCN | yes | no | no | no | no | yes | yes | no | no |

| | | | | | | | | | | | |
|---|---|---|---|---|---|---|---|---|---|---|---|
| 40 OPT/C | F | Non-ectatic control | *N/A* | no | no | no | no | no | yes | no | no |
| 41 OPT/C | M | Non-ectatic control | *N/A* | yes | no | no | no | no | yes | no | no |
| 42 OPT/C | M | Non-ectatic control | *N/A* | no | no | no | no | yes | yes | yes | no |
| 43 OPT/KTCN | M | KTCN | no | no | no | no | no | yes | yes | no | yes |
| 44 OPT/C | M | Non-ectatic control | *N/A* | yes | no | no | no | no | yes | yes | no |
| 44 OPT/KTCN | M | KTCN | yes | no | no | no | no | no | yes | no | yes |
| 45 OPT/C | M | Non-ectatic control | *N/A* | no | no | no | no | no | no | no | no |
| 48 OPT/C | M | Non-ectatic control | *N/A* | no | no | no | no | yes | yes | no | no |
| 4F/6 | M | Non-ectatic control | *N/A* | no | no | no | no | yes | no | no | no |
| 4F/7 | M | Non-ectatic control | *N/A* | no | no | no | no | yes | no | no | no |
| 4 OPT/M | F | Non-ectatic control | *N/A* | no | no | no | no | no | no | no | no |
| 4 OPT/KTCN | M | KTCN | yes | no | no | no | no | yes | no | no | no |
| 4 WUM/KTCN | M | KTCN | yes | no | no | no | no | no | yes | no | no |
| 50 OPT/C | M | Non-ectatic control | *N/A* | no | no | no | no | no | no | no | no |
| 51 OPT/C | M | Non-ectatic control | *N/A* | yes | no | yes | yes | no | yes | yes | no |
| 51 OPT/KTCN | M | PLVC | *N/A* | no | no | no | no | no | no | no | no |
| 57 OPT/KTCN | F | KTCN | Yes | yes | no | no | no | no | yes | yes | no |
| 5 OPT/M | M | Non-ectatic control | *N/A* | no | no | no | no | no | yes | *N/D* | yes |
| 5F/1 | F | KTCN | *N/A* | *N/D* | *N/D* | *N/D* | *N/D* | *N/D* | *N/D* | *N/D* | *N/D* |
| 5 OPT/KTCN | M | KTCN | no | yes | no | no | no | no | no | no | no |
| 5 WUM/KTCN | M | KTCN | yes | no | no | no | no | no | no | no | no |
| 62 OPT/KTCN | M | PLVC | *N/A* | no | no | no | no | yes | yes | no | no |
| 63 OPT/KTCN | M | KTCN | no | no | no | no | no | no | yes | yes | yes |

| | | | | | | | | | | | |
|---|---|---|---|---|---|---|---|---|---|---|---|
| 64 OPT/KTCN | M | KTCN | yes | no | no | no | no | no | yes | yes | no |
| 66 OPT/KTCN | M | KTCN | no | no | no | no | no | yes | yes | yes | no |
| 69 OPT/KTCN | M | KTCN | yes | no | no | no | no | no | yes | yes | no |
| 6 OPT/M | M | Non-ectatic control | *N/A* | yes | no | no | no | no | yes | no | yes |
| 6 OPT/KTCN | M | KTCN | no | no | no | no | no | yes | yes | *N/D* | yes |
| 6 WUM/KTCN | M | KTCN | yes | no | no | no | no | yes | yes | *N/D* | *N/D* |
| 70 OPT/KTCN | F | KTCN | No | no | no | no | no | no | yes | no | no |
| 71 OPT/KTCN | M | PMD | *N/A* | no | no | no | no | yes | yes | yes | no |
| 76 OPT/KTCN | M | KTCN | yes | no | no | no | no | no | yes | yes | no |
| 79 OPT/KTCN | F | KTCN | yes | no | no | no | no | no | no | no | no |
| 7 OPT/M | M | Non-ectatic control | *N/A* | no | no | no | no | yes | yes | no | yes |
| 7 OPT/KTCN | M | KTCN | yes | no | no | no | no | yes | yes | no | yes |
| 7 WUM/KTCN | M | KTCN | yes | no | no | no | no | no | yes | yes | no |
| 80 OPT/KTCN | M | KTCN | yes | no | no | no | no | no | no | no | no |
| 81 OPT/KTCN | M | KTCN | yes | no | no | no | no | no | yes | no | yes |
| 83 OPT/KTCN | M | KTCN | yes | yes | no | no | no | no | yes | no | yes |
| 84 OPT/KTCN | M | KTCN | no | no | no | no | no | yes | yes | yes | yes |
| 89 OPT/KTCN | M | KTCN | no | no | no | no | no | no | yes | yes | no |
| 8 OPT/M | M | Non-ectatic control | *N/A* | no | no | no | no | no | yes | no | yes |
| 8 OPT/KTCN | F | KTCN | no | no | no | no | no | no | yes | *N/D* | no |
| 91 OPT/KTCN | M | KTCN | yes | yes | yes | yes | yes | no | yes | no | yes |
| 95 OPT/KTCN | M | PLVC | *N/A* | no | no | no | no | no | yes | no | no |
| 96 OPT/KTCN | F | KTCN | no | no | no | no | no | no | yes | yes | no |
| 98 OPT/KTCN | M | KTCN | no | yes | no | yes | yes | no | yes | yes | no |
| 99 OPT/KTCN | M | PMD | *N/A* | no | no | no | no | no | yes | no | no |
| 9 OPT/C | M | Non-ectatic control | *N/A* | yes | no | no | no | no | yes | no | no |
| 9 OPT/KTCN | M | KTCN | no | no | no | no | no | no | yes | yes | no |
| 9 WUM/KTCN | F | KTCN | yes | no | no | no | no | no | yes | no | no |

Abbreviations in the Table: *N/A* – not applicable; *N/D* – no data; KTCN – keratoconus; PLVC – post-laser vision correction ectasia; PMD – pellucid marginal degeneration; M – male; F – female;

Given the small subgroup sizes and the underlying biological overlap between these conditions, the clinical categories “atopy” and “asthma” were merged into a single variable for statistical analyses. This decision was supported by the sample structure: in our cohort, only one out of nine individuals with asthma did not have an accompanying atopic or allergic background. Because atopy and asthma share common immunopathological pathways and often co-occur, their consolidation improved statistical power while maintaining biological interpretability.

**Supplementary Table S3. The results of statistical comparisons of quantitative clinical data**

Results of the Kruskal–Wallis tests and Dunn's post-hoc tests, including test statistics, p-values, and p-values corrected using the Bonferroni method (pbonf) and the Holm–Bonferroni method (pholm), are presented for the following clinical variables: age, thinnest corneal thickness (TCT), flat keratometry (K1), steep keratometry (K2), maximal corneal curvature (Kmax), anterior elevation, and posterior elevation.

A) **Age** (KTCN *n*=93, non-ectatic control *n*=44, PMD *n*=4, PLVC *n*=10)

Kruskal-Wallis test

| Factor | Statistic | Df | p |
|---|---|---|---|
| Diagnosis | 15.6883 | 3 | 0.0013 |

Dunn's Post Hoc Comparisons

| Comparison | Z | Wi | Wj | rrb | p | pbonf | pholm |
|---|---|---|---|---|---|---|---|
| KTCN - non-ectatic control | -1.2670 | 68.5860 | 78.7159 | 0.1454 | 0.2051 | 1.0000 | 0.4103 |
| KTCN - PMD | -2.8421 | 68.5860 | 132.0000 | 0.7742 | 0.0045 | 0.0269 | 0.0232 |
| KTCN - PLVC | -2.8893 | 68.5860 | 110.6000 | 0.5333 | 0.0039 | 0.0232 | 0.0232 |
| non-ectatic control - PMD | -2.3351 | 78.7159 | 132.0000 | 0.7841 | 0.0195 | 0.1172 | 0.0781 |
| non-ectatic control - PLVC | -2.0829 | 78.7159 | 110.6000 | 0.4955 | 0.0373 | 0.2235 | 0.1118 |
| PMD - PLVC | 0.8279 | 132.0000 | 110.6000 | 0.5500 | 0.4078 | 1.0000 | 0.4103 |

Note. Rank-biserial correlation based on individual Mann-Whitney tests.

B) **TCT** (KTCN *n*=90, non-ectatic control *n*=43, PMD *n*=4, PLVC *n*=10)

Kruskal-Wallis test

| Factor | Statistic | Df | p |
|---|---|---|---|
| Diagnosis | 78.5166 | 3 | < 0.001 |

Dunn's Post Hoc Comparisons

| Comparison | Z | Wi | Wj | rrb | p | pbonf | pholm |
|---|---|---|---|---|---|---|---|
| KTCN - non-ectatic control | -8.5143 | 53.1556 | 120.3605 | 0.9152 | < 0.001 | < 0.001 | < 0.001 |
| KTCN - PMD | -2.4978 | 53.1556 | 107.5000 | 0.8611 | 0.0125 | 0.0750 | 0.0500 |
| KTCN - PLVC | 0.3034 | 53.1556 | 48.8500 | 0.1111 | 0.7616 | 1.0000 | 1.0000 |
| non-ectatic control - PMD | 0.5778 | 120.3605 | 107.5000 | 0.4186 | 0.5634 | 1.0000 | 1.0000 |
| non-ectatic control - PLVC | 4.7839 | 120.3605 | 48.8500 | 0.8674 | < 0.001 | < 0.001 | < 0.001 |
| PMD - PLVC | 2.3284 | 107.5000 | 48.8500 | 0.7500 | 0.0199 | 0.1194 | 0.0597 |

Note. Rank-biserial correlation based on individual Mann-Whitney tests.

C) **K1** (KTCN *n*=87, non-ectatic control *n*=44, PMD *n*=4, PLVC *n*=10)

Kruskal-Wallis test

| Factor | Statistic | df | p |
|---|---|---|---|
| Diagnosis | 41.0738 | 3 | < 0.001 |

Dunn's Post Hoc Comparisons

| Comparison | z | Wi | Wj | rrb | p | pbonf | pholm |
|---|---|---|---|---|---|---|---|
| KTCN - non-ectatic control | 5.2734 | 90.8908 | 49.9205 | 0.5909 | < 0.001 | < 0.001 | < 0.001 |
| KTCN - PMD | 2.3173 | 90.8908 | 41.1250 | 0.6437 | 0.0205 | 0.1229 | 0.0820 |
| KTCN - PLVC | 4.2244 | 90.8908 | 31.6500 | 0.7207 | < 0.001 | < 0.001 | < 0.001 |
| non-ectatic control - PMD | 0.4010 | 49.9205 | 41.1250 | 0.2102 | 0.6884 | 1.0000 | 1.0000 |
| non-ectatic control - PLVC | 1.2418 | 49.9205 | 31.6500 | 0.4409 | 0.2143 | 1.0000 | 0.6429 |
| PMD - PLVC | 0.3813 | 41.1250 | 31.6500 | 0.1500 | 0.7029 | 1.0000 | 1.0000 |

Note. Rank-biserial correlation based on individual Mann-Whitney tests.

**D) K2** (KTCN *n*=87, non-ectatic control *n*=44, PMD *n*=4, PLVC *n*=10)

Kruskal-Wallis test

| Factor | Statistic | df | p |
|---|---|---|---|
| Diagnosis | 67.7567 | 3 | < 0.001 |

Dunn's Post Hoc Comparisons

| Comparison | z | Wi | Wj | rrb | p | pbonf | pholm |
|---|---|---|---|---|---|---|---|
| KTCN - non-ectatic control | 7.6767 | 95.7356 | 36.0909 | 0.8352 | < 0.001 | < 0.001 | < 0.001 |
| KTCN - PMD | 0.7560 | 95.7356 | 79.5000 | 0.3851 | 0.4497 | 1.0000 | 0.8994 |
| KTCN - PLVC | 4.3308 | 95.7356 | 35.0000 | 0.7184 | < 0.001 | < 0.001 | < 0.001 |
| non-ectatic control - PMD | -1.9791 | 36.0909 | 79.5000 | 0.9091 | 0.0478 | 0.2868 | 0.1912 |
| non-ectatic control - PLVC | 0.0741 | 36.0909 | 35.0000 | 0.2477 | 0.9409 | 1.0000 | 0.9409 |
| PMD - PLVC | 1.7909 | 79.5000 | 35.0000 | 0.6500 | 0.0733 | 0.4398 | 0.2199 |

Note. Rank-biserial correlation based on individual Mann-Whitney tests.

**E) Kmax** (KTCN *n*=86, non-ectatic control *n*=44, PMD *n*=4, PLVC *n*=10)

Kruskal-Wallis test

| Factor | Statistic | df | p |
|---|---|---|---|
| Diagnosis | 94.5779 | 3 | < 0.001 |

Dunn's Post Hoc Comparisons

| Comparison | z | Wi | Wj | rrb | p | pbonf | pholm |
|---|---|---|---|---|---|---|---|
| KTCN - non-ectatic control | 9.6465 | 99.0349 | 24.4545 | 0.9926 | < 0.001 | < 0.001 | < 0.001 |
| KTCN - PMD | 1.4019 | 99.0349 | 69.1250 | 0.6221 | 0.1609 | 0.9657 | 0.3219 |

| | | | | | | | |
|---|---|---|---|---|---|---|---|
| KTCN - PLVC | 3.0126 | 99.0349 | 57.0500 | 0.6907 | 0.0026 | 0.0155 | 0.0129 |
| non-ectatic control - PMD | -2.0507 | 24.4545 | 69.1250 | 0.9830 | 0.0403 | 0.2418 | 0.1209 |
| non-ectatic control - PLVC | -2.2306 | 24.4545 | 57.0500 | 0.6795 | 0.0257 | 0.1542 | 0.1028 |
| PMD - PLVC | 0.4893 | 69.1250 | 57.0500 | 0.3500 | 0.6246 | 1.0000 | 0.6246 |

Note. Rank-biserial correlation based on individual Mann-Whitney tests.

F) **Anterior Elevation** (KTCN *n*=83, non-ectatic control *n*=44, PMD *n*=4, PLVC *n*=10

Kruskal-Wallis test

| Factor | Statistic | df | p |
|---|---|---|---|
| Diagnosis | 92.9571 | 3 | < 0.001 |

Dunn's Post Hoc Comparisons

| Comparison | z | Wi | Wj | rrb | p | pbonf | pholm |
|---|---|---|---|---|---|---|---|
| KTCN - non-ectatic control | 9.4069 | 94.6205 | 23.0227 | 0.9904 | < 0.001 | < 0.001 | < 0.001 |
| KTCN - PMD | -0.9036 | 94.6205 | 113.5000 | 0.4096 | 0.3662 | 1.0000 | 0.3662 |
| KTCN - PLVC | 1.8716 | 94.6205 | 69.0500 | 0.5301 | 0.0613 | 0.3675 | 0.1838 |
| non-ectatic control - PMD | -4.2448 | 23.0227 | 113.5000 | 1.0000 | < 0.001 | < 0.001 | < 0.001 |
| non-ectatic control - PLVC | -3.2191 | 23.0227 | 69.0500 | 0.9750 | 0.0013 | 0.0077 | 0.0051 |
| PMD - PLVC | 1.8409 | 113.5000 | 69.0500 | 0.7000 | 0.0656 | 0.3938 | 0.1838 |

Note. Rank-biserial correlation based on individual Mann-Whitney tests.

G) **Posterior Elevation** (KTCN *n*=82, non-ectatic control *n*=44, PMD *n*=4, PLVC *n*=10

Kruskal-Wallis test

| Factor | Statistic | df | p |
|---|---|---|---|
| Diagnosis | 95.2446 | 3 | < 0.001 |

Dunn's Post Hoc Comparisons

| Comparison | z | Wi | Wj | rrb | p | pbonf | pholm |
|---|---|---|---|---|---|---|---|
| KTCN - non-ectatic control | 9.4921 | 94.6524 | 22.7273 | 0.9961 | < 0.001 | < 0.001 | < 0.001 |
| KTCN - PMD | -0.9921 | 94.6524 | 115.2500 | 0.4512 | 0.3212 | 1.0000 | 0.3212 |
| KTCN - PLVC | 2.2017 | 94.6524 | 64.7500 | 0.6280 | 0.0277 | 0.1661 | 0.0831 |
| non-ectatic control - PMD | -4.3694 | 22.7273 | 115.2500 | 1.0000 | < 0.001 | < 0.001 | < 0.001 |
| non-ectatic control - PLVC | -2.9583 | 22.7273 | 64.7500 | 0.9864 | 0.0031 | 0.0186 | 0.0124 |
| PMD - PLVC | 2.1052 | 115.2500 | 64.7500 | 0.8500 | 0.0353 | 0.2116 | 0.0831 |

Note. Rank-biserial correlation based on individual Mann-Whitney tests.

**Supplementary Table S4. The list of m/z peaks detected in serum samples, together with fragment sequences, any identified modifications, and the corresponding gene names**

| m/z peak | sequence | Modification | Gene name |
|---|---|---|---|
| 927.4911 | R.NSPGVPTGAK.K | x | GOLGA2 |
| 928.4933 | R.NSPGVPTGAK.K | x | GOLGA2 |
| 933.5152 | R.NSPGVPTGAK.K | x | GOLGA2 |
| 940.4501 | K.LCTVATLR.E | Carbamidomethyl: 2 | ALB |
| 961.5606 | K.LCTVATLR.E | Carbamidomethyl: 2 | ALB |
| 1074.5395 | K.SAVQGPPER.D | x | IGHA1 |
| 1160.5839 | K.NVDTNQDR.L | x | NUCB1 |
| 1283.5692 | K.NVDTNQDR.L | x | NUCB1 |
| 1312.7379 | K.CCTESLVNR.R | Carbamidomethyl: 1, 2 | ALB |
| 1453.8203 | K.LGDNEETQVR.T | x | C14orf37 |
| 1467.8387 | K.EGYYGYTGAFR.C | x | TF |
| 1469.8445 | R.HPDYSVVLLLR.L | x | ALB |
| 1478.7290 | R.HPDYSVVLLLR.L | x | ALB |
| 1495.6742 | x | x | x |
| 1575.7855 | x | x | x |
| 1577.7891 | R.RHPDYSVVLLLR.L | x | ALB |
| 1623.7807 | R.RHPDYSVVLLLR.L | x | ALB |
| 1625.7858 | R.RHPDYSVVLLLR.L | x | ALB |
| 1627.7886 | R.RHPDYSVVLLLR.L | x | ALB |
| 1640.7716 | K.GTWTQPFDLASTR.E | x | SERPINA6 |
| 1680.7961 | R.YYCFQGNQFLR.F | Carbamidomethyl: 3 | HPX |
| 1699.8509 | R.YYCFQGNQFLR.F | Carbamidomethyl: 3 | HPX |
| 1701.8575 | R.ILGADTSVDLEETGR.V | x | ATP5A1 |
| 1742.8747 | R.ILGADTSVDLEETGR.V | x | ATP5A1 |
| 1744.8789 | R.ILGADTSVDLEETGR.V | x | ATP5A1 |
| 1764.8613 | K.DVFLGMFLYEYAR.R | x | ALB |

| | | | |
|---|---|---|---|
| 1766.8663 | K.DVFLGMFLYEYAR.R | x | ALB |
| 1781.8427 | K.DVFLGMFLYEYAR.R | x | ALB |
| 1797.8849 | K.DVFLGMFLYEYAR.R | x | ALB |
| 1799.8909 | K.DVFLGMFLYEYAR.R | x | ALB |
| 1884.9355 | K.DVFLGMFLYEYAR.R | Oxidation: 6 | ALB |
| 1886.9310 | K.DVFLGMFLYEYAR.R | Oxidation: 6 | ALB |
| 1899.9858 | K.DVFLGMFLYEYAR.R | Oxidation: 6 | ALB |
| 1901.9886 | K.MDATANDVPSPYEVR.G | Oxidation: 1 | PDIA3 |
| 1903.9873 | K.MDATANDVPSPYEVR.G | Oxidation: 1 | PDIA3 |
| 1910.9266 | x | x | x |
| 1912.9334 | x | x | x |
| 1914.9441 | x | x | x |
| 1921.9655 | x | x | x |
| 1936.9343 | K.LYPIANGNNQSPVDIK.T | x | CA1 |
| 1938.9411 | K.LYPIANGNNQSPVDIK.T | x | CA1 |
| 1957.3335 | x | x | x |
| 1958.9888 | x | x | x |
| 2046.5348 | x | x | x |
| 2171.7616 | K.VDATEESDLAQQYGVR.G | x | P4HB |
| 2173.8434 | K.VDATEESDLAQQYGVR.G | x | P4HB |
| 2175.8031 | K.VDATEESDLAQQYGVR.G | x | P4HB |
| 2513.2352 | K.SGTASVVCLLNNFYPR.E | Carbamidomethyl: 8 | IGKC |
| 2519.1934 | K.SGTASVVCLLNNFYPR.E | Carbamidomethyl: 8 | IGKC |
| 2540.1730 | K.SGTASVVCLLNNFYPR.E | Carbamidomethyl: 8 | IGKC |
| 2542.1741 | K.SGTASVVCLLNNFYPR.E | Carbamidomethyl: 8 | IGKC |
| 2546.1616 | K.STVSSLLQKEICPLIR.I | Carbamidomethyl: 12; Acetyl: 1 | BPIFA2 |
| 2557.1730 | K.STVSSLLQKEICPLIR.I | Carbamidomethyl: 12; Acetyl: 1 | BPIFA2 |
| 2585.1617 | x | x | x |
| 2587.1421 | x | x | x |
| 2600.2757 | x | x | x |

| | | | |
|---|---|---|---|
| 2650.2586 | x | x | x |
| 2652.2540 | x | x | x |
| 2674.2991 | x | x | x |
| 2676.3062 | R.RPCFSALEVDETYVPK.E | Carbamidomethyl: 3 | ALB |
| 2678.3062 | R.RPCFSALEVDETYVPK.E | Carbamidomethyl: 3 | ALB |
| 2680.2991 | R.RPCFSALEVDETYVPK.E | Carbamidomethyl: 3 | ALB |
| 2732.3330 | R.RPCFSALEVDETYVPK.E | Carbamidomethyl: 3 | ALB |
| 2734.3338 | R.RPCFSALEVDETYVPK.E | Carbamidomethyl: 3 | ALB |
| 2779.3570 | R.DQEGQDVLLFIDNIFR.F | x | ATP5B |
| 2781.3635 | R.DQEGQDVLLFIDNIFR.F | x | ATP5B |
| 2802.2766 | K.LPQGYHPNDVEEEWGK.L | Acetyl: 1 | MACF1 |
| 927.4911 | R.VAPEEHPTLLTEAPLNPK.A | x | ACTC1 |
| 928.4933 | R.VAPEEHPTLLTEAPLNPK.A | x | ACTC1 |
| 933.5152 | R.VAPEEHPTLLTEAPLNPK.A | x | ACTC1 |
| 940.4501 | K.GHYTEGAELVDSVLDVVR.K | x | TUBB |
| 961.5606 | K.QNCELFEQLGEYK.F | Carbamidomethyl: 3 | ALB |
| 1074.5395 | K.QNCELFEQLGEYK.F | Carbamidomethyl: 3 | ALB |
| 1160.5839 | K.QNCELFEQLGEYK.F | Carbamidomethyl: 3 | ALB |
| 1283.5692 | K.LISVDTEHSNIYLQNGPDR.I | x | CP |
| 1312.7379 | K.LISVDTEHSNIYLQNGPDR.I | x | CP |
| 1453.8203 | K.LISVDTEHSNIYLQNGPDR.I | x | CP |
| 1467.8387 | K.LISVDTEHSNIYLQNGPDR.I | x | CP |
| 1469.8445 | K.LISVDTEHSNIYLQNGPDR.I | x | CP |
| 1478.7290 | x | x | x |
| 1495.6742 | x | x | x |
| 1575.7855 | x | x | x |
| 1577.7891 | x | x | x |
| 1623.7807 | x | x | x |
| 1625.7858 | x | x | x |
| 1627.7886 | x | x | x |

| | | | |
|---|---|---|---|
| 1640.7716 | x | x | x |
| 1680.7961 | K.EFNAETFTFHADICTLSEKER.Q | Carbamidomethyl: 14 | ALB |
| 1699.8509 | K.EFNAETFTFHADICTLSEKER.Q | Carbamidomethyl: 14 | ALB |
| 1701.8575 | K.EFNAETFTFHADICTLSEKER.Q | Carbamidomethyl: 14 | ALB |
| 1742.8747 | x | x | x |
| 1744.8789 | x | x | x |
| 1764.8613 | x | x | x |
| 1766.8663 | x | x | x |
| 1781.8427 | K.QNCELFEQLGEYKFQNALLVR.Y | Carbamidomethyl: 3 | ALB |
| 1797.8849 | K.QNCELFEQLGEYKFQNALLVR.Y | Carbamidomethyl: 3 | ALB |
| 1799.8909 | P.GVLSSRQLGLPGPPDVPDHAAYHPF.R | x | ITIH4 |
| 1884.9355 | R.LVRPEVDVMCTAFHDNEETFLK.K | Carbamidomethyl: 10 | ALB |
| 1886.9310 | R.LVRPEVDVMCTAFHDNEETFLK.K | Carbamidomethyl: 10 | ALB |
| 1899.9858 | R.LVRPEVDVMCTAFHDNEETFLK.K | Carbamidomethyl: 10 | ALB |
| 1901.9886 | R.LVRPEVDVMCTAFHDNEETFLK.K | Carbamidomethyl: 10 | ALB |
| 1903.9873 | K.RMPCAEDYLSVVLNQLCVLHEK.T | Carbamidomethyl: 4, 17 | ALB |
| 1910.9266 | K.RMPCAEDYLSVVLNQLCVLHEK.T | Carbamidomethyl: 4, 17 | ALB |
| 1912.9334 | K.RMPCAEDYLSVVLNQLCVLHEK.T | Carbamidomethyl: 4, 17 | ALB |
| 1914.9441 | K.RMPCAEDYLSVVLNQLCVLHEK.T | Carbamidomethyl: 4, 17 | ALB |
| 1921.9655 | K.RMPCAEDYLSVVLNQLCVLHEK.T | Carbamidomethyl: 4, 17 | ALB |
| 1936.9343 | K.RMPCAEDYLSVVLNQLCVLHEK.T | Carbamidomethyl: 4, 17 | ALB |
| 1938.9411 | K.RMPCAEDYLSVVLNQLCVLHEK.T | Carbamidomethyl: 4, 17 | ALB |
| 1957.3335 | K.CPFPSRPDNGFVNYPAKPTLYYK.D | Carbamidomethyl: 1 | APOH |
| 1958.9888 | K.CPFPSRPDNGFVNYPAKPTLYYK.D | Carbamidomethyl: 1 | APOH |
| 2046.5348 | K.CPFPSRPDNGFVNYPAKPTLYYK.D | Carbamidomethyl: 1 | APOH |
| 2171.7616 | K.CPFPSRPDNGFVNYPAKPTLYYK.D | Carbamidomethyl: 1 | APOH |
| 2173.8434 | R.STQDTVIALDALSAYWIASHTTEER.G | X | C4B |
| 2175.8031 | R.STQDTVIALDALSAYWIASHTTEER.G | X | C4B |
| 2513.2352 | R.STQDTVIALDALSAYWIASHTTEER.G | X | C4B |
| 2519.1934 | R.STQDTVIALDALSAYWIASHTTEER.G | X | C4B |

| | | | |
|---|---|---|---|
| 2540.1730 | R.STQDTVIALDALSAYWIASHTTEER.G | X | C4B |
| 2542.1741 | R.WQEGNVFSCSVMHEALHNHYTQK.S | Carbamidomethyl: 9 | IGHG4 |
| 2546.1616 | R.WQEGNVFSCSVMHEALHNHYTQK.S | Carbamidomethyl: 9 | IGHG4 |

**Supplementary Table S5. Descriptive statistics (including means, medians, standard deviations, and minimum and maximum values) of m/z peak intensities stratified by diagnosis (KTCN *n*=93, non-ectatic control *n*=44, PMD *n*=4, PLVC *n*=10)**

| m/z peak | Diagnosis | Median | Mean | SD | Minimum | Maximum |
|---|---|---|---|---|---|---|
| 927.4911 | KTCN | 0.0027 | 0.0029 | 0.0011 | 0.0012 | 0.0058 |
| 927.4911 | non-ectatic control | 0.0032 | 0.0032 | 0.0010 | 0.0014 | 0.0050 |
| 927.4911 | PMD | 0.0021 | 0.0022 | 0.0009 | 0.0013 | 0.0033 |
| 927.4911 | PLVC | 0.0025 | 0.0029 | 0.0018 | 0.0014 | 0.0073 |
| 928.4933 | KTCN | 0.0018 | 0.0021 | 0.0008 | 0.0005 | 0.0054 |
| 928.4933 | non-ectatic control | 0.0022 | 0.0022 | 0.0006 | 0.0010 | 0.0034 |
| 928.4933 | PMD | 0.0016 | 0.0016 | 0.0005 | 0.0012 | 0.0022 |
| 928.4933 | PLVC | 0.0020 | 0.0022 | 0.0011 | 0.0011 | 0.0050 |
| 929.4949 | KTCN | 0.0013 | 0.0013 | 0.0005 | 0.0004 | 0.0032 |
| 929.4949 | non-ectatic control | 0.0014 | 0.0014 | 0.0003 | 0.0004 | 0.0020 |
| 929.4949 | PMD | 0.0010 | 0.0011 | 0.0004 | 0.0009 | 0.0017 |
| 929.4949 | PLVC | 0.0012 | 0.0013 | 0.0005 | 0.0004 | 0.0025 |
| 933.5152 | KTCN | 0.0014 | 0.0014 | 0.0004 | 0.0004 | 0.0024 |
| 933.5152 | non-ectatic control | 0.0014 | 0.0014 | 0.0003 | 0.0009 | 0.0024 |
| 933.5152 | PMD | 0.0013 | 0.0014 | 0.0004 | 0.0011 | 0.0020 |
| 933.5152 | PLVC | 0.0013 | 0.0015 | 0.0004 | 0.0011 | 0.0023 |
| 934.5163 | KTCN | 0.0012 | 0.0012 | 0.0003 | 0.0004 | 0.0019 |
| 934.5163 | non-ectatic control | 0.0012 | 0.0011 | 0.0003 | 0.0004 | 0.0017 |
| 934.5163 | PMD | 0.0012 | 0.0010 | 0.0004 | 0.0004 | 0.0013 |
| 934.5163 | PLVC | 0.0013 | 0.0013 | 0.0002 | 0.0010 | 0.0017 |
| 940.4501 | KTCN | 0.0011 | 0.0011 | 0.0003 | 0.0004 | 0.0020 |
| 940.4501 | non-ectatic control | 0.0011 | 0.0011 | 0.0004 | 0.0004 | 0.0018 |
| 940.4501 | PMD | 0.0011 | 0.0010 | 0.0004 | 0.0004 | 0.0014 |
| 940.4501 | PLVC | 0.0012 | 0.0012 | 0.0004 | 0.0004 | 0.0017 |
| 960.5602 | KTCN | 0.0023 | 0.0025 | 0.0009 | 0.0005 | 0.0053 |
| 960.5602 | non-ectatic control | 0.0025 | 0.0027 | 0.0009 | 0.0012 | 0.0048 |

| | | | | | | |
|---|---|---|---|---|---|---|
| 960.5602 | PMD | 0.0017 | 0.0020 | 0.0009 | 0.0012 | 0.0032 |
| 960.5602 | PLVC | 0.0023 | 0.0024 | 0.0011 | 0.0009 | 0.0043 |
| 961.5606 | KTCN | 0.0017 | 0.0019 | 0.0007 | 0.0005 | 0.0046 |
| 961.5606 | non-ectatic control | 0.0018 | 0.0020 | 0.0006 | 0.0012 | 0.0033 |
| 961.5606 | PMD | 0.0015 | 0.0016 | 0.0005 | 0.0012 | 0.0023 |
| 961.5606 | PLVC | 0.0018 | 0.0019 | 0.0007 | 0.0005 | 0.0029 |
| 962.5605 | KTCN | 0.0012 | 0.0012 | 0.0004 | 0.0004 | 0.0033 |
| 962.5605 | non-ectatic control | 0.0012 | 0.0013 | 0.0003 | 0.0004 | 0.0018 |
| 962.5605 | PMD | 0.0011 | 0.0010 | 0.0004 | 0.0004 | 0.0012 |
| 962.5605 | PLVC | 0.0012 | 0.0013 | 0.0004 | 0.0004 | 0.0019 |
| 1074.5395 | KTCN | 0.0011 | 0.0011 | 0.0002 | 0.0004 | 0.0017 |
| 1074.5395 | non-ectatic control | 0.0011 | 0.0011 | 0.0003 | 0.0004 | 0.0015 |
| 1074.5395 | PMD | 0.0011 | 0.0010 | 0.0004 | 0.0004 | 0.0013 |
| 1074.5395 | PLVC | 0.0011 | 0.0012 | 0.0006 | 0.0004 | 0.0028 |
| 1138.4990 | KTCN | 0.0011 | 0.0011 | 0.0004 | 0.0004 | 0.0028 |
| 1138.4990 | non-ectatic control | 0.0011 | 0.0012 | 0.0002 | 0.0004 | 0.0019 |
| 1138.4990 | PMD | 0.0011 | 0.0011 | 0.0002 | 0.0009 | 0.0014 |
| 1138.4990 | PLVC | 0.0012 | 0.0013 | 0.0004 | 0.0009 | 0.0024 |
| 1160.5839 | KTCN | 0.0015 | 0.0015 | 0.0004 | 0.0004 | 0.0026 |
| 1160.5839 | non-ectatic control | 0.0014 | 0.0015 | 0.0004 | 0.0004 | 0.0025 |
| 1160.5839 | PMD | 0.0013 | 0.0013 | 0.0002 | 0.0011 | 0.0015 |
| 1160.5839 | PLVC | 0.0014 | 0.0014 | 0.0004 | 0.0004 | 0.0021 |
| 1161.5873 | KTCN | 0.0013 | 0.0013 | 0.0004 | 0.0005 | 0.0024 |
| 1161.5873 | non-ectatic control | 0.0013 | 0.0013 | 0.0003 | 0.0005 | 0.0021 |
| 1161.5873 | PMD | 0.0013 | 0.0013 | 0.0001 | 0.0012 | 0.0015 |
| 1161.5873 | PLVC | 0.0013 | 0.0012 | 0.0003 | 0.0005 | 0.0016 |
| 1283.5692 | KTCN | 0.0011 | 0.0010 | 0.0004 | 0.0004 | 0.0018 |
| 1283.5692 | non-ectatic control | 0.0011 | 0.0011 | 0.0003 | 0.0004 | 0.0017 |
| 1283.5692 | PMD | 0.0009 | 0.0009 | 0.0004 | 0.0004 | 0.0014 |
| 1283.5692 | PLVC | 0.0011 | 0.0011 | 0.0004 | 0.0004 | 0.0016 |

| | | | | | | |
|---|---|---|---|---|---|---|
| 1311.7370 | KTCN | 0.0015 | 0.0016 | 0.0005 | 0.0004 | 0.0036 |
| 1311.7370 | non-ectatic control | 0.0016 | 0.0016 | 0.0005 | 0.0004 | 0.0027 |
| 1311.7370 | PMD | 0.0014 | 0.0014 | 0.0001 | 0.0013 | 0.0016 |
| 1311.7370 | PLVC | 0.0015 | 0.0015 | 0.0005 | 0.0010 | 0.0026 |
| 1312.7379 | KTCN | 0.0014 | 0.0015 | 0.0004 | 0.0004 | 0.0029 |
| 1312.7379 | non-ectatic control | 0.0014 | 0.0014 | 0.0004 | 0.0009 | 0.0024 |
| 1312.7379 | PMD | 0.0014 | 0.0014 | 0.0001 | 0.0012 | 0.0015 |
| 1312.7379 | PLVC | 0.0013 | 0.0013 | 0.0005 | 0.0004 | 0.0024 |
| 1313.7424 | KTCN | 0.0011 | 0.0011 | 0.0003 | 0.0004 | 0.0022 |
| 1313.7424 | non-ectatic control | 0.0011 | 0.0010 | 0.0003 | 0.0004 | 0.0015 |
| 1313.7424 | PMD | 0.0010 | 0.0010 | 9.0910×10-5 | 0.0009 | 0.0011 |
| 1313.7424 | PLVC | 0.0010 | 0.0011 | 0.0003 | 0.0004 | 0.0014 |
| 1453.8203 | KTCN | 0.0011 | 0.0011 | 0.0003 | 0.0004 | 0.0019 |
| 1453.8203 | non-ectatic control | 0.0012 | 0.0012 | 0.0003 | 0.0004 | 0.0021 |
| 1453.8203 | PMD | 0.0010 | 0.0010 | 0.0001 | 0.0009 | 0.0012 |
| 1453.8203 | PLVC | 0.0011 | 0.0010 | 0.0004 | 0.0004 | 0.0016 |
| 1454.8085 | KTCN | 0.0011 | 0.0010 | 0.0003 | 0.0004 | 0.0017 |
| 1454.8085 | non-ectatic control | 0.0011 | 0.0011 | 0.0002 | 0.0004 | 0.0018 |
| 1454.8085 | PMD | 0.0010 | 0.0010 | 7.0353×10-5 | 0.0009 | 0.0011 |
| 1454.8085 | PLVC | 0.0011 | 0.0011 | 0.0001 | 0.0009 | 0.0013 |
| 1467.8387 | KTCN | 0.0026 | 0.0029 | 0.0012 | 0.0011 | 0.0065 |
| 1467.8387 | non-ectatic control | 0.0031 | 0.0035 | 0.0016 | 0.0015 | 0.0073 |
| 1467.8387 | PMD | 0.0020 | 0.0021 | 0.0005 | 0.0017 | 0.0028 |
| 1467.8387 | PLVC | 0.0025 | 0.0028 | 0.0015 | 0.0010 | 0.0064 |
| 1468.8427 | KTCN | 0.0023 | 0.0025 | 0.0010 | 0.0010 | 0.0057 |
| 1468.8427 | non-ectatic control | 0.0026 | 0.0031 | 0.0014 | 0.0015 | 0.0066 |
| 1468.8427 | PMD | 0.0018 | 0.0019 | 0.0005 | 0.0013 | 0.0025 |
| 1468.8427 | PLVC | 0.0024 | 0.0025 | 0.0012 | 0.0014 | 0.0057 |
| 1469.8445 | KTCN | 0.0015 | 0.0017 | 0.0006 | 0.0004 | 0.0033 |
| 1469.8445 | non-ectatic control | 0.0017 | 0.0020 | 0.0007 | 0.0012 | 0.0039 |

| | | | | | | |
|---|---|---|---|---|---|---|
| 1469.8445 | PMD | 0.0014 | 0.0015 | 0.0002 | 0.0012 | 0.0017 |
| 1469.8445 | PLVC | 0.0016 | 0.0017 | 0.0007 | 0.0009 | 0.0035 |
| 1470.8480 | KTCN | 0.0011 | 0.0012 | 0.0003 | 0.0004 | 0.0023 |
| 1470.8480 | non-ectatic control | 0.0012 | 0.0013 | 0.0003 | 0.0009 | 0.0021 |
| 1470.8480 | PMD | 0.0010 | 0.0011 | 0.0001 | 0.0009 | 0.0012 |
| 1470.8480 | PLVC | 0.0011 | 0.0011 | 0.0005 | 0.0004 | 0.0021 |
| 1478.7290 | KTCN | 0.0011 | 0.0011 | 0.0003 | 0.0004 | 0.0019 |
| 1478.7290 | non-ectatic control | 0.0012 | 0.0011 | 0.0003 | 0.0004 | 0.0018 |
| 1478.7290 | PMD | 0.0009 | 0.0010 | 0.0002 | 0.0009 | 0.0013 |
| 1478.7290 | PLVC | 0.0011 | 0.0010 | 0.0002 | 0.0004 | 0.0012 |
| 1479.7321 | KTCN | 0.0011 | 0.0011 | 0.0003 | 0.0004 | 0.0018 |
| 1479.7321 | non-ectatic control | 0.0011 | 0.0011 | 0.0002 | 0.0004 | 0.0014 |
| 1479.7321 | PMD | 0.0010 | 0.0009 | 0.0003 | 0.0004 | 0.0011 |
| 1479.7321 | PLVC | 0.0011 | 0.0010 | 0.0003 | 0.0004 | 0.0014 |
| 1495.6742 | KTCN | 0.0011 | 0.0011 | 0.0003 | 0.0004 | 0.0018 |
| 1495.6742 | non-ectatic control | 0.0012 | 0.0012 | 0.0002 | 0.0004 | 0.0015 |
| 1495.6742 | PMD | 0.0011 | 0.0011 | 0.0001 | 0.0010 | 0.0013 |
| 1495.6742 | PLVC | 0.0011 | 0.0010 | 0.0003 | 0.0004 | 0.0013 |
| 1496.6791 | KTCN | 0.0011 | 0.0010 | 0.0003 | 0.0004 | 0.0019 |
| 1496.6791 | non-ectatic control | 0.0011 | 0.0011 | 0.0003 | 0.0004 | 0.0015 |
| 1496.6791 | PMD | 0.0007 | 0.0007 | 0.0004 | 0.0004 | 0.0012 |
| 1496.6791 | PLVC | 0.0011 | 0.0010 | 0.0003 | 0.0004 | 0.0013 |
| 1575.7855 | KTCN | 0.0016 | 0.0017 | 0.0008 | 0.0005 | 0.0051 |
| 1575.7855 | non-ectatic control | 0.0018 | 0.0020 | 0.0009 | 0.0009 | 0.0048 |
| 1575.7855 | PMD | 0.0011 | 0.0011 | 0.0002 | 0.0009 | 0.0015 |
| 1575.7855 | PLVC | 0.0014 | 0.0017 | 0.0009 | 0.0009 | 0.0034 |
| 1576.7886 | KTCN | 0.0015 | 0.0016 | 0.0008 | 0.0004 | 0.0050 |
| 1576.7886 | non-ectatic control | 0.0016 | 0.0019 | 0.0008 | 0.0009 | 0.0045 |
| 1576.7886 | PMD | 0.0011 | 0.0011 | 0.0002 | 0.0009 | 0.0013 |
| 1576.7886 | PLVC | 0.0015 | 0.0017 | 0.0007 | 0.0010 | 0.0028 |

| | | | | | | |
|---|---|---|---|---|---|---|
| 1577.7891 | KTCN | 0.0013 | 0.0013 | 0.0005 | 0.0004 | 0.0034 |
| 1577.7891 | non-ectatic control | 0.0013 | 0.0015 | 0.0006 | 0.0004 | 0.0031 |
| 1577.7891 | PMD | 0.0010 | 0.0009 | 0.0003 | 0.0004 | 0.0011 |
| 1577.7891 | PLVC | 0.0012 | 0.0013 | 0.0006 | 0.0004 | 0.0020 |
| 1578.7894 | KTCN | 0.0011 | 0.0010 | 0.0003 | 0.0004 | 0.0021 |
| 1578.7894 | non-ectatic control | 0.0011 | 0.0011 | 0.0003 | 0.0004 | 0.0017 |
| 1578.7894 | PMD | 0.0009 | 0.0008 | 0.0003 | 0.0004 | 0.0010 |
| 1578.7894 | PLVC | 0.0011 | 0.0010 | 0.0004 | 0.0004 | 0.0013 |
| 1623.7807 | KTCN | 0.0038 | 0.0039 | 0.0017 | 0.0009 | 0.0080 |
| 1623.7807 | non-ectatic control | 0.0044 | 0.0043 | 0.0019 | 0.0015 | 0.0094 |
| 1623.7807 | PMD | 0.0033 | 0.0035 | 0.0013 | 0.0022 | 0.0051 |
| 1623.7807 | PLVC | 0.0032 | 0.0030 | 0.0011 | 0.0015 | 0.0049 |
| 1624.7830 | KTCN | 0.0036 | 0.0038 | 0.0016 | 0.0010 | 0.0077 |
| 1624.7830 | non-ectatic control | 0.0040 | 0.0042 | 0.0018 | 0.0014 | 0.0089 |
| 1624.7830 | PMD | 0.0030 | 0.0033 | 0.0014 | 0.0019 | 0.0052 |
| 1624.7830 | PLVC | 0.0029 | 0.0028 | 0.0010 | 0.0014 | 0.0044 |
| 1625.7858 | KTCN | 0.0025 | 0.0026 | 0.0010 | 0.0010 | 0.0056 |
| 1625.7858 | non-ectatic control | 0.0027 | 0.0029 | 0.0012 | 0.0011 | 0.0060 |
| 1625.7858 | PMD | 0.0022 | 0.0024 | 0.0010 | 0.0016 | 0.0038 |
| 1625.7858 | PLVC | 0.0023 | 0.0021 | 0.0006 | 0.0012 | 0.0030 |
| 1626.7889 | KTCN | 0.0017 | 0.0017 | 0.0005 | 0.0005 | 0.0029 |
| 1626.7889 | non-ectatic control | 0.0017 | 0.0018 | 0.0006 | 0.0010 | 0.0036 |
| 1626.7889 | PMD | 0.0015 | 0.0015 | 0.0003 | 0.0012 | 0.0018 |
| 1626.7889 | PLVC | 0.0015 | 0.0014 | 0.0003 | 0.0010 | 0.0019 |
| 1627.7886 | KTCN | 0.0012 | 0.0011 | 0.0003 | 0.0004 | 0.0016 |
| 1627.7886 | non-ectatic control | 0.0012 | 0.0011 | 0.0003 | 0.0004 | 0.0017 |
| 1627.7886 | PMD | 0.0012 | 0.0012 | 0.0001 | 0.0010 | 0.0013 |
| 1627.7886 | PLVC | 0.0011 | 0.0011 | 0.0005 | 0.0004 | 0.0022 |
| 1639.7759 | KTCN | 0.0011 | 0.0011 | 0.0003 | 0.0004 | 0.0018 |
| 1639.7759 | non-ectatic control | 0.0011 | 0.0011 | 0.0003 | 0.0004 | 0.0016 |

| | | | | | | |
|---|---|---|---|---|---|---|
| 1639.7759 | PMD | 0.0011 | 0.0010 | 0.0004 | 0.0004 | 0.0013 |
| 1639.7759 | PLVC | 0.0010 | 0.0009 | 0.0003 | 0.0004 | 0.0013 |
| 1640.7716 | KTCN | 0.0011 | 0.0010 | 0.0003 | 0.0004 | 0.0016 |
| 1640.7716 | non-ectatic control | 0.0012 | 0.0011 | 0.0003 | 0.0004 | 0.0016 |
| 1640.7716 | PMD | 0.0010 | 0.0009 | 0.0004 | 0.0004 | 0.0013 |
| 1640.7716 | PLVC | 0.0010 | 0.0010 | 0.0003 | 0.0004 | 0.0015 |
| 1641.8376 | KTCN | 0.0011 | 0.0012 | 0.0003 | 0.0004 | 0.0032 |
| 1641.8376 | non-ectatic control | 0.0012 | 0.0012 | 0.0003 | 0.0009 | 0.0027 |
| 1641.8376 | PMD | 0.0010 | 0.0009 | 0.0003 | 0.0004 | 0.0012 |
| 1641.8376 | PLVC | 0.0012 | 0.0013 | 0.0006 | 0.0009 | 0.0029 |
| 1680.7961 | KTCN | 0.0010 | 0.0010 | 0.0004 | 0.0004 | 0.0022 |
| 1680.7961 | non-ectatic control | 0.0011 | 0.0010 | 0.0003 | 0.0004 | 0.0016 |
| 1680.7961 | PMD | 0.0009 | 0.0008 | 0.0002 | 0.0004 | 0.0010 |
| 1680.7961 | PLVC | 0.0010 | 0.0009 | 0.0004 | 0.0004 | 0.0014 |
| 1681.8040 | KTCN | 0.0010 | 0.0010 | 0.0003 | 0.0004 | 0.0019 |
| 1681.8040 | non-ectatic control | 0.0011 | 0.0010 | 0.0003 | 0.0004 | 0.0017 |
| 1681.8040 | PMD | 0.0007 | 0.0007 | 0.0004 | 0.0004 | 0.0012 |
| 1681.8040 | PLVC | 0.0010 | 0.0010 | 0.0003 | 0.0004 | 0.0014 |
| 1699.8509 | KTCN | 0.0017 | 0.0017 | 0.0005 | 0.0004 | 0.0029 |
| 1699.8509 | non-ectatic control | 0.0017 | 0.0017 | 0.0005 | 0.0010 | 0.0027 |
| 1699.8509 | PMD | 0.0016 | 0.0017 | 0.0005 | 0.0013 | 0.0023 |
| 1699.8509 | PLVC | 0.0015 | 0.0017 | 0.0006 | 0.0010 | 0.0029 |
| 1700.8546 | KTCN | 0.0017 | 0.0017 | 0.0005 | 0.0004 | 0.0027 |
| 1700.8546 | non-ectatic control | 0.0017 | 0.0017 | 0.0005 | 0.0004 | 0.0027 |
| 1700.8546 | PMD | 0.0017 | 0.0017 | 0.0005 | 0.0011 | 0.0024 |
| 1700.8546 | PLVC | 0.0016 | 0.0017 | 0.0005 | 0.0011 | 0.0028 |
| 1701.8575 | KTCN | 0.0013 | 0.0013 | 0.0003 | 0.0005 | 0.0021 |
| 1701.8575 | non-ectatic control | 0.0013 | 0.0013 | 0.0003 | 0.0005 | 0.0019 |
| 1701.8575 | PMD | 0.0012 | 0.0013 | 0.0002 | 0.0011 | 0.0016 |
| 1701.8575 | PLVC | 0.0011 | 0.0013 | 0.0004 | 0.0009 | 0.0021 |

| | | | | | | |
|---|---|---|---|---|---|---|
| 1702.8553 | KTCN | 0.0011 | 0.0010 | 0.0003 | 0.0004 | 0.0019 |
| 1702.8553 | non-ectatic control | 0.0010 | 0.0010 | 0.0003 | 0.0004 | 0.0014 |
| 1702.8553 | PMD | 0.0011 | 0.0011 | 0.0001 | 0.0010 | 0.0013 |
| 1702.8553 | PLVC | 0.0011 | 0.0010 | 0.0003 | 0.0004 | 0.0014 |
| 1742.8747 | KTCN | 0.0016 | 0.0016 | 0.0004 | 0.0005 | 0.0025 |
| 1742.8747 | non-ectatic control | 0.0016 | 0.0016 | 0.0004 | 0.0011 | 0.0026 |
| 1742.8747 | PMD | 0.0017 | 0.0017 | 0.0003 | 0.0014 | 0.0022 |
| 1742.8747 | PLVC | 0.0015 | 0.0015 | 0.0004 | 0.0009 | 0.0021 |
| 1743.8789 | KTCN | 0.0016 | 0.0016 | 0.0005 | 0.0004 | 0.0026 |
| 1743.8789 | non-ectatic control | 0.0016 | 0.0016 | 0.0004 | 0.0010 | 0.0027 |
| 1743.8789 | PMD | 0.0018 | 0.0018 | 0.0006 | 0.0011 | 0.0026 |
| 1743.8789 | PLVC | 0.0015 | 0.0016 | 0.0004 | 0.0011 | 0.0024 |
| 1744.8789 | KTCN | 0.0013 | 0.0013 | 0.0003 | 0.0005 | 0.0019 |
| 1744.8789 | non-ectatic control | 0.0013 | 0.0013 | 0.0002 | 0.0010 | 0.0021 |
| 1744.8789 | PMD | 0.0013 | 0.0014 | 0.0001 | 0.0013 | 0.0015 |
| 1744.8789 | PLVC | 0.0013 | 0.0013 | 0.0004 | 0.0005 | 0.0018 |
| 1745.8788 | KTCN | 0.0011 | 0.0010 | 0.0003 | 0.0004 | 0.0015 |
| 1745.8788 | non-ectatic control | 0.0010 | 0.0010 | 0.0003 | 0.0004 | 0.0014 |
| 1745.8788 | PMD | 0.0010 | 0.0010 | 0.0001 | 0.0009 | 0.0011 |
| 1745.8788 | PLVC | 0.0010 | 0.0010 | 0.0002 | 0.0004 | 0.0013 |
| 1764.8613 | KTCN | 0.0013 | 0.0013 | 0.0003 | 0.0004 | 0.0023 |
| 1764.8613 | non-ectatic control | 0.0013 | 0.0013 | 0.0003 | 0.0004 | 0.0024 |
| 1764.8613 | PMD | 0.0014 | 0.0014 | 0.0002 | 0.0012 | 0.0016 |
| 1764.8613 | PLVC | 0.0015 | 0.0015 | 0.0003 | 0.0009 | 0.0020 |
| 1765.8635 | KTCN | 0.0014 | 0.0014 | 0.0004 | 0.0004 | 0.0024 |
| 1765.8635 | non-ectatic control | 0.0013 | 0.0014 | 0.0003 | 0.0004 | 0.0025 |
| 1765.8635 | PMD | 0.0014 | 0.0014 | 0.0002 | 0.0010 | 0.0016 |
| 1765.8635 | PLVC | 0.0015 | 0.0014 | 0.0004 | 0.0004 | 0.0021 |
| 1766.8663 | KTCN | 0.0012 | 0.0012 | 0.0003 | 0.0004 | 0.0020 |
| 1766.8663 | non-ectatic control | 0.0012 | 0.0012 | 0.0002 | 0.0009 | 0.0019 |

| | | | | | | |
|---|---|---|---|---|---|---|
| 1766.8663 | PMD | 0.0011 | 0.0011 | 0.0003 | 0.0009 | 0.0014 |
| 1766.8663 | PLVC | 0.0012 | 0.0012 | 0.0003 | 0.0004 | 0.0016 |
| 1780.8377 | KTCN | 0.0011 | 0.0012 | 0.0004 | 0.0004 | 0.0029 |
| 1780.8377 | non-ectatic control | 0.0011 | 0.0012 | 0.0004 | 0.0004 | 0.0025 |
| 1780.8377 | PMD | 0.0011 | 0.0011 | 8.1375×10-5 | 0.0010 | 0.0012 |
| 1780.8377 | PLVC | 0.0012 | 0.0013 | 0.0004 | 0.0004 | 0.0020 |
| 1781.8427 | KTCN | 0.0011 | 0.0012 | 0.0005 | 0.0004 | 0.0028 |
| 1781.8427 | non-ectatic control | 0.0011 | 0.0013 | 0.0004 | 0.0004 | 0.0028 |
| 1781.8427 | PMD | 0.0011 | 0.0011 | 9.9079×10-5 | 0.0010 | 0.0012 |
| 1781.8427 | PLVC | 0.0012 | 0.0013 | 0.0005 | 0.0004 | 0.0020 |
| 1782.8403 | KTCN | 0.0011 | 0.0011 | 0.0004 | 0.0004 | 0.0022 |
| 1782.8403 | non-ectatic control | 0.0010 | 0.0011 | 0.0004 | 0.0004 | 0.0022 |
| 1782.8403 | PMD | 0.0010 | 0.0009 | 0.0003 | 0.0004 | 0.0011 |
| 1782.8403 | PLVC | 0.0012 | 0.0011 | 0.0003 | 0.0004 | 0.0016 |
| 1797.8849 | KTCN | 0.0016 | 0.0018 | 0.0006 | 0.0005 | 0.0036 |
| 1797.8849 | non-ectatic control | 0.0018 | 0.0019 | 0.0006 | 0.0010 | 0.0033 |
| 1797.8849 | PMD | 0.0014 | 0.0014 | 0.0002 | 0.0012 | 0.0017 |
| 1797.8849 | PLVC | 0.0018 | 0.0018 | 0.0006 | 0.0010 | 0.0029 |
| 1798.8883 | KTCN | 0.0017 | 0.0018 | 0.0006 | 0.0004 | 0.0036 |
| 1798.8883 | non-ectatic control | 0.0018 | 0.0019 | 0.0006 | 0.0010 | 0.0035 |
| 1798.8883 | PMD | 0.0014 | 0.0014 | 0.0003 | 0.0012 | 0.0018 |
| 1798.8883 | PLVC | 0.0016 | 0.0018 | 0.0006 | 0.0009 | 0.0029 |
| 1799.8909 | KTCN | 0.0014 | 0.0015 | 0.0004 | 0.0005 | 0.0029 |
| 1799.8909 | non-ectatic control | 0.0015 | 0.0016 | 0.0004 | 0.0009 | 0.0026 |
| 1799.8909 | PMD | 0.0012 | 0.0012 | 0.0002 | 0.0010 | 0.0015 |
| 1799.8909 | PLVC | 0.0015 | 0.0015 | 0.0004 | 0.0005 | 0.0022 |
| 1800.8943 | KTCN | 0.0011 | 0.0012 | 0.0003 | 0.0004 | 0.0019 |
| 1800.8943 | non-ectatic control | 0.0012 | 0.0012 | 0.0002 | 0.0004 | 0.0016 |
| 1800.8943 | PMD | 0.0010 | 0.0010 | 0.0005 | 0.0004 | 0.0016 |
| 1800.8943 | PLVC | 0.0011 | 0.0011 | 0.0003 | 0.0004 | 0.0014 |

| | | | | | | |
|---|---|---|---|---|---|---|
| 1884.9355 | KTCN | 0.0011 | 0.0011 | 0.0003 | 0.0004 | 0.0017 |
| 1884.9355 | non-ectatic control | 0.0011 | 0.0012 | 0.0003 | 0.0004 | 0.0017 |
| 1884.9355 | PMD | 0.0010 | 0.0010 | 0.0004 | 0.0004 | 0.0014 |
| 1884.9355 | PLVC | 0.0011 | 0.0011 | 0.0003 | 0.0004 | 0.0014 |
| 1885.9333 | KTCN | 0.0011 | 0.0011 | 0.0003 | 0.0004 | 0.0018 |
| 1885.9333 | non-ectatic control | 0.0012 | 0.0012 | 0.0002 | 0.0009 | 0.0018 |
| 1885.9333 | PMD | 0.0010 | 0.0010 | 0.0001 | 0.0009 | 0.0011 |
| 1885.9333 | PLVC | 0.0012 | 0.0012 | 0.0002 | 0.0009 | 0.0014 |
| 1886.9310 | KTCN | 0.0010 | 0.0010 | 0.0003 | 0.0004 | 0.0014 |
| 1886.9310 | non-ectatic control | 0.0011 | 0.0010 | 0.0002 | 0.0004 | 0.0014 |
| 1886.9310 | PMD | 0.0007 | 0.0007 | 0.0004 | 0.0004 | 0.0011 |
| 1886.9310 | PLVC | 0.0010 | 0.0010 | 0.0003 | 0.0004 | 0.0014 |
| 1898.9815 | KTCN | 0.0054 | 0.0064 | 0.0029 | 0.0021 | 0.0139 |
| 1898.9815 | non-ectatic control | 0.0067 | 0.0079 | 0.0031 | 0.0030 | 0.0152 |
| 1898.9815 | PMD | 0.0049 | 0.0047 | 0.0012 | 0.0032 | 0.0059 |
| 1898.9815 | PLVC | 0.0062 | 0.0063 | 0.0021 | 0.0031 | 0.0100 |
| 1899.9858 | KTCN | 0.0060 | 0.0070 | 0.0031 | 0.0023 | 0.0151 |
| 1899.9858 | non-ectatic control | 0.0073 | 0.0086 | 0.0034 | 0.0033 | 0.0165 |
| 1899.9858 | PMD | 0.0054 | 0.0052 | 0.0011 | 0.0038 | 0.0063 |
| 1899.9858 | PLVC | 0.0066 | 0.0067 | 0.0022 | 0.0030 | 0.0107 |
| 1900.9876 | KTCN | 0.0043 | 0.0051 | 0.0022 | 0.0019 | 0.0111 |
| 1900.9876 | non-ectatic control | 0.0053 | 0.0062 | 0.0025 | 0.0024 | 0.0124 |
| 1900.9876 | PMD | 0.0040 | 0.0039 | 0.0008 | 0.0028 | 0.0046 |
| 1900.9876 | PLVC | 0.0049 | 0.0050 | 0.0016 | 0.0023 | 0.0077 |
| 1901.9886 | KTCN | 0.0026 | 0.0030 | 0.0012 | 0.0011 | 0.0065 |
| 1901.9886 | non-ectatic control | 0.0030 | 0.0036 | 0.0014 | 0.0015 | 0.0071 |
| 1901.9886 | PMD | 0.0023 | 0.0022 | 0.0005 | 0.0016 | 0.0026 |
| 1901.9886 | PLVC | 0.0028 | 0.0030 | 0.0010 | 0.0014 | 0.0046 |
| 1902.9914 | KTCN | 0.0016 | 0.0017 | 0.0005 | 0.0010 | 0.0033 |
| 1902.9914 | non-ectatic control | 0.0017 | 0.0019 | 0.0006 | 0.0011 | 0.0035 |

| | | | | | | |
|---|---|---|---|---|---|---|
| 1902.9914 | PMD | 0.0014 | 0.0015 | 0.0002 | 0.0013 | 0.0018 |
| 1902.9914 | PLVC | 0.0015 | 0.0017 | 0.0004 | 0.0011 | 0.0023 |
| 1903.9873 | KTCN | 0.0011 | 0.0011 | 0.0003 | 0.0004 | 0.0017 |
| 1903.9873 | non-ectatic control | 0.0012 | 0.0012 | 0.0002 | 0.0009 | 0.0018 |
| 1903.9873 | PMD | 0.0009 | 0.0010 | 9.2795×10-5 | 0.0009 | 0.0011 |
| 1903.9873 | PLVC | 0.0011 | 0.0010 | 0.0002 | 0.0004 | 0.0013 |
| 1904.9665 | KTCN | 0.0010 | 0.0010 | 0.0003 | 0.0004 | 0.0022 |
| 1904.9665 | non-ectatic control | 0.0011 | 0.0011 | 0.0002 | 0.0004 | 0.0015 |
| 1904.9665 | PMD | 0.0010 | 0.0010 | 4.0077×10-5 | 0.0009 | 0.0010 |
| 1904.9665 | PLVC | 0.0010 | 0.0010 | 0.0004 | 0.0004 | 0.0019 |
| 1910.9266 | KTCN | 0.0019 | 0.0021 | 0.0010 | 0.0004 | 0.0052 |
| 1910.9266 | non-ectatic control | 0.0021 | 0.0024 | 0.0013 | 0.0004 | 0.0055 |
| 1910.9266 | PMD | 0.0015 | 0.0017 | 0.0006 | 0.0012 | 0.0025 |
| 1910.9266 | PLVC | 0.0020 | 0.0020 | 0.0009 | 0.0004 | 0.0037 |
| 1911.9291 | KTCN | 0.0019 | 0.0022 | 0.0011 | 0.0005 | 0.0057 |
| 1911.9291 | non-ectatic control | 0.0021 | 0.0025 | 0.0012 | 0.0009 | 0.0056 |
| 1911.9291 | PMD | 0.0014 | 0.0017 | 0.0009 | 0.0010 | 0.0030 |
| 1911.9291 | PLVC | 0.0023 | 0.0022 | 0.0010 | 0.0005 | 0.0040 |
| 1912.9334 | KTCN | 0.0016 | 0.0017 | 0.0008 | 0.0005 | 0.0041 |
| 1912.9334 | non-ectatic control | 0.0017 | 0.0019 | 0.0009 | 0.0009 | 0.0042 |
| 1912.9334 | PMD | 0.0013 | 0.0014 | 0.0006 | 0.0009 | 0.0022 |
| 1912.9334 | PLVC | 0.0016 | 0.0017 | 0.0007 | 0.0005 | 0.0030 |
| 1913.9330 | KTCN | 0.0012 | 0.0013 | 0.0005 | 0.0004 | 0.0031 |
| 1913.9330 | non-ectatic control | 0.0013 | 0.0014 | 0.0005 | 0.0004 | 0.0025 |
| 1913.9330 | PMD | 0.0012 | 0.0012 | 0.0003 | 0.0009 | 0.0016 |
| 1913.9330 | PLVC | 0.0013 | 0.0013 | 0.0004 | 0.0004 | 0.0019 |
| 1914.9441 | KTCN | 0.0011 | 0.0011 | 0.0004 | 0.0004 | 0.0024 |
| 1914.9441 | non-ectatic control | 0.0011 | 0.0010 | 0.0003 | 0.0004 | 0.0017 |
| 1914.9441 | PMD | 0.0010 | 0.0009 | 0.0003 | 0.0004 | 0.0011 |
| 1914.9441 | PLVC | 0.0010 | 0.0010 | 0.0002 | 0.0004 | 0.0012 |

| | | | | | | |
|---|---|---|---|---|---|---|
| 1920.9605 | KTCN | 0.0012 | 0.0013 | 0.0003 | 0.0004 | 0.0029 |
| 1920.9605 | non-ectatic control | 0.0014 | 0.0014 | 0.0003 | 0.0009 | 0.0021 |
| 1920.9605 | PMD | 0.0012 | 0.0012 | 0.0001 | 0.0010 | 0.0014 |
| 1920.9605 | PLVC | 0.0013 | 0.0014 | 0.0003 | 0.0011 | 0.0019 |
| 1921.9655 | KTCN | 0.0013 | 0.0013 | 0.0004 | 0.0004 | 0.0030 |
| 1921.9655 | non-ectatic control | 0.0014 | 0.0015 | 0.0003 | 0.0010 | 0.0022 |
| 1921.9655 | PMD | 0.0011 | 0.0011 | 9.9928×10-5 | 0.0009 | 0.0012 |
| 1921.9655 | PLVC | 0.0016 | 0.0014 | 0.0005 | 0.0004 | 0.0022 |
| 1922.9664 | KTCN | 0.0011 | 0.0011 | 0.0003 | 0.0005 | 0.0024 |
| 1922.9664 | non-ectatic control | 0.0012 | 0.0012 | 0.0002 | 0.0009 | 0.0017 |
| 1922.9664 | PMD | 0.0010 | 0.0009 | 0.0003 | 0.0005 | 0.0010 |
| 1922.9664 | PLVC | 0.0012 | 0.0013 | 0.0003 | 0.0009 | 0.0018 |
| 1936.9343 | KTCN | 0.0011 | 0.0011 | 0.0004 | 0.0004 | 0.0022 |
| 1936.9343 | non-ectatic control | 0.0011 | 0.0012 | 0.0003 | 0.0004 | 0.0019 |
| 1936.9343 | PMD | 0.0009 | 0.0009 | 0.0004 | 0.0004 | 0.0014 |
| 1936.9343 | PLVC | 0.0012 | 0.0012 | 0.0004 | 0.0004 | 0.0021 |
| 1937.9369 | KTCN | 0.0011 | 0.0011 | 0.0004 | 0.0004 | 0.0023 |
| 1937.9369 | non-ectatic control | 0.0011 | 0.0012 | 0.0004 | 0.0004 | 0.0020 |
| 1937.9369 | PMD | 0.0011 | 0.0011 | 0.0002 | 0.0009 | 0.0014 |
| 1937.9369 | PLVC | 0.0012 | 0.0013 | 0.0004 | 0.0010 | 0.0022 |
| 1938.9411 | KTCN | 0.0010 | 0.0010 | 0.0004 | 0.0004 | 0.0020 |
| 1938.9411 | non-ectatic control | 0.0010 | 0.0011 | 0.0003 | 0.0004 | 0.0018 |
| 1938.9411 | PMD | 0.0009 | 0.0009 | 0.0004 | 0.0004 | 0.0014 |
| 1938.9411 | PLVC | 0.0011 | 0.0011 | 0.0003 | 0.0004 | 0.0016 |
| 1956.1162 | KTCN | 0.0013 | 0.0013 | 0.0005 | 0.0004 | 0.0041 |
| 1956.1162 | non-ectatic control | 0.0014 | 0.0015 | 0.0005 | 0.0009 | 0.0033 |
| 1956.1162 | PMD | 0.0010 | 0.0010 | 9.6726×10-5 | 0.0009 | 0.0011 |
| 1956.1162 | PLVC | 0.0015 | 0.0015 | 0.0005 | 0.0009 | 0.0023 |
| 1957.3335 | KTCN | 0.0013 | 0.0014 | 0.0006 | 0.0004 | 0.0045 |
| 1957.3335 | non-ectatic control | 0.0014 | 0.0016 | 0.0006 | 0.0009 | 0.0037 |

| | | | | | | |
|---|---|---|---|---|---|---|
| 1957.3335 | PMD | 0.0011 | 0.0011 | 7.2296×10-5 | 0.0010 | 0.0011 |
| 1957.3335 | PLVC | 0.0015 | 0.0015 | 0.0005 | 0.0004 | 0.0023 |
| 1958.8175 | KTCN | 0.0012 | 0.0012 | 0.0005 | 0.0004 | 0.0035 |
| 1958.8175 | non-ectatic control | 0.0013 | 0.0014 | 0.0004 | 0.0009 | 0.0027 |
| 1958.8175 | PMD | 0.0007 | 0.0008 | 0.0004 | 0.0004 | 0.0012 |
| 1958.8175 | PLVC | 0.0013 | 0.0013 | 0.0003 | 0.0009 | 0.0019 |
| 1958.9888 | KTCN | 0.0010 | 0.0010 | 0.0003 | 0.0004 | 0.0020 |
| 1958.9888 | non-ectatic control | 0.0011 | 0.0011 | 0.0003 | 0.0004 | 0.0017 |
| 1958.9888 | PMD | 0.0004 | 0.0006 | 0.0003 | 0.0004 | 0.0010 |
| 1958.9888 | PLVC | 0.0012 | 0.0010 | 0.0003 | 0.0004 | 0.0014 |
| 2045.4943 | KTCN | 0.0012 | 0.0012 | 0.0003 | 0.0004 | 0.0021 |
| 2045.4943 | non-ectatic control | 0.0013 | 0.0012 | 0.0003 | 0.0004 | 0.0018 |
| 2045.4943 | PMD | 0.0011 | 0.0010 | 0.0004 | 0.0004 | 0.0014 |
| 2045.4943 | PLVC | 0.0011 | 0.0012 | 0.0005 | 0.0004 | 0.0025 |
| 2046.5348 | KTCN | 0.0012 | 0.0012 | 0.0003 | 0.0004 | 0.0023 |
| 2046.5348 | non-ectatic control | 0.0013 | 0.0013 | 0.0003 | 0.0004 | 0.0019 |
| 2046.5348 | PMD | 0.0012 | 0.0012 | 0.0001 | 0.0011 | 0.0014 |
| 2046.5348 | PLVC | 0.0012 | 0.0013 | 0.0005 | 0.0004 | 0.0025 |
| 2047.5768 | KTCN | 0.0011 | 0.0011 | 0.0003 | 0.0004 | 0.0017 |
| 2047.5768 | non-ectatic control | 0.0011 | 0.0011 | 0.0003 | 0.0004 | 0.0016 |
| 2047.5768 | PMD | 0.0012 | 0.0010 | 0.0004 | 0.0004 | 0.0012 |
| 2047.5768 | PLVC | 0.0010 | 0.0011 | 0.0004 | 0.0004 | 0.0021 |
| 2171.7616 | KTCN | 0.0019 | 0.0019 | 0.0005 | 0.0009 | 0.0029 |
| 2171.7616 | non-ectatic control | 0.0019 | 0.0020 | 0.0004 | 0.0012 | 0.0033 |
| 2171.7616 | PMD | 0.0018 | 0.0018 | 0.0004 | 0.0014 | 0.0022 |
| 2171.7616 | PLVC | 0.0019 | 0.0018 | 0.0004 | 0.0011 | 0.0022 |
| 2172.8074 | KTCN | 0.0021 | 0.0020 | 0.0006 | 0.0005 | 0.0034 |
| 2172.8074 | non-ectatic control | 0.0022 | 0.0022 | 0.0005 | 0.0010 | 0.0035 |
| 2172.8074 | PMD | 0.0019 | 0.0020 | 0.0004 | 0.0017 | 0.0025 |
| 2172.8074 | PLVC | 0.0021 | 0.0020 | 0.0004 | 0.0010 | 0.0025 |

| | | | | | | |
|---|---|---|---|---|---|---|
| 2173.8434 | KTCN | 0.0017 | 0.0017 | 0.0004 | 0.0009 | 0.0027 |
| 2173.8434 | non-ectatic control | 0.0018 | 0.0018 | 0.0004 | 0.0011 | 0.0029 |
| 2173.8434 | PMD | 0.0017 | 0.0016 | 0.0002 | 0.0012 | 0.0017 |
| 2173.8434 | PLVC | 0.0018 | 0.0018 | 0.0003 | 0.0011 | 0.0021 |
| 2174.8570 | KTCN | 0.0013 | 0.0013 | 0.0003 | 0.0004 | 0.0019 |
| 2174.8570 | non-ectatic control | 0.0014 | 0.0014 | 0.0002 | 0.0004 | 0.0019 |
| 2174.8570 | PMD | 0.0013 | 0.0013 | 4.8826×10-5 | 0.0012 | 0.0013 |
| 2174.8570 | PLVC | 0.0014 | 0.0013 | 0.0003 | 0.0004 | 0.0016 |
| 2175.8031 | KTCN | 0.0011 | 0.0011 | 0.0002 | 0.0004 | 0.0015 |
| 2175.8031 | non-ectatic control | 0.0011 | 0.0011 | 0.0002 | 0.0004 | 0.0013 |
| 2175.8031 | PMD | 0.0010 | 0.0009 | 0.0003 | 0.0004 | 0.0010 |
| 2175.8031 | PLVC | 0.0012 | 0.0011 | 0.0003 | 0.0004 | 0.0013 |
| 2491.2670 | KTCN | 0.0011 | 0.0010 | 0.0003 | 0.0004 | 0.0016 |
| 2491.2670 | non-ectatic control | 0.0011 | 0.0010 | 0.0003 | 0.0004 | 0.0014 |
| 2491.2670 | PMD | 0.0012 | 0.0012 | 0.0001 | 0.0010 | 0.0013 |
| 2491.2670 | PLVC | 0.0010 | 0.0010 | 0.0002 | 0.0004 | 0.0012 |
| 2513.2352 | KTCN | 0.0011 | 0.0011 | 0.0003 | 0.0004 | 0.0015 |
| 2513.2352 | non-ectatic control | 0.0011 | 0.0011 | 0.0003 | 0.0004 | 0.0016 |
| 2513.2352 | PMD | 0.0010 | 0.0011 | 0.0001 | 0.0009 | 0.0012 |
| 2513.2352 | PLVC | 0.0011 | 0.0010 | 0.0002 | 0.0004 | 0.0013 |
| 2518.1940 | KTCN | 0.0011 | 0.0011 | 0.0004 | 0.0004 | 0.0019 |
| 2518.1940 | non-ectatic control | 0.0011 | 0.0010 | 0.0004 | 0.0004 | 0.0017 |
| 2518.1940 | PMD | 0.0012 | 0.0011 | 0.0005 | 0.0004 | 0.0016 |
| 2518.1940 | PLVC | 0.0011 | 0.0011 | 0.0003 | 0.0004 | 0.0016 |
| 2519.1934 | KTCN | 0.0012 | 0.0012 | 0.0004 | 0.0004 | 0.0019 |
| 2519.1934 | non-ectatic control | 0.0011 | 0.0011 | 0.0004 | 0.0004 | 0.0021 |
| 2519.1934 | PMD | 0.0013 | 0.0012 | 0.0005 | 0.0004 | 0.0017 |
| 2519.1934 | PLVC | 0.0012 | 0.0012 | 0.0003 | 0.0009 | 0.0020 |
| 2520.1957 | KTCN | 0.0012 | 0.0011 | 0.0003 | 0.0004 | 0.0018 |
| 2520.1957 | non-ectatic control | 0.0011 | 0.0011 | 0.0004 | 0.0004 | 0.0019 |

| | | | | | | |
|---|---|---|---|---|---|---|
| 2520.1957 | PMD | 0.0013 | 0.0013 | 0.0003 | 0.0009 | 0.0016 |
| 2520.1957 | PLVC | 0.0011 | 0.0012 | 0.0003 | 0.0009 | 0.0018 |
| 2540.1730 | KTCN | 0.0010 | 0.0010 | 0.0003 | 0.0004 | 0.0019 |
| 2540.1730 | non-ectatic control | 0.0010 | 0.0010 | 0.0004 | 0.0004 | 0.0018 |
| 2540.1730 | PMD | 0.0011 | 0.0010 | 0.0004 | 0.0004 | 0.0013 |
| 2540.1730 | PLVC | 0.0010 | 0.0010 | 0.0003 | 0.0004 | 0.0015 |
| 2541.1731 | KTCN | 0.0011 | 0.0011 | 0.0003 | 0.0004 | 0.0021 |
| 2541.1731 | non-ectatic control | 0.0012 | 0.0012 | 0.0004 | 0.0004 | 0.0020 |
| 2541.1731 | PMD | 0.0011 | 0.0010 | 0.0005 | 0.0004 | 0.0014 |
| 2541.1731 | PLVC | 0.0012 | 0.0012 | 0.0003 | 0.0004 | 0.0017 |
| 2542.1741 | KTCN | 0.0011 | 0.0011 | 0.0003 | 0.0004 | 0.0020 |
| 2542.1741 | non-ectatic control | 0.0011 | 0.0011 | 0.0003 | 0.0004 | 0.0018 |
| 2542.1741 | PMD | 0.0011 | 0.0010 | 0.0004 | 0.0004 | 0.0014 |
| 2542.1741 | PLVC | 0.0011 | 0.0010 | 0.0003 | 0.0004 | 0.0015 |
| 2545.1604 | KTCN | 0.0011 | 0.0011 | 0.0004 | 0.0004 | 0.0024 |
| 2545.1604 | non-ectatic control | 0.0011 | 0.0013 | 0.0005 | 0.0004 | 0.0029 |
| 2545.1604 | PMD | 0.0009 | 0.0009 | 0.0003 | 0.0004 | 0.0012 |
| 2545.1604 | PLVC | 0.0012 | 0.0011 | 0.0004 | 0.0004 | 0.0014 |
| 2546.1616 | KTCN | 0.0011 | 0.0012 | 0.0006 | 0.0004 | 0.0030 |
| 2546.1616 | non-ectatic control | 0.0012 | 0.0014 | 0.0007 | 0.0004 | 0.0035 |
| 2546.1616 | PMD | 0.0009 | 0.0009 | 0.0003 | 0.0004 | 0.0011 |
| 2546.1616 | PLVC | 0.0012 | 0.0011 | 0.0003 | 0.0004 | 0.0015 |
| 2547.1667 | KTCN | 0.0011 | 0.0011 | 0.0005 | 0.0004 | 0.0024 |
| 2547.1667 | non-ectatic control | 0.0011 | 0.0013 | 0.0006 | 0.0004 | 0.0027 |
| 2547.1667 | PMD | 0.0009 | 0.0008 | 0.0003 | 0.0004 | 0.0011 |
| 2547.1667 | PLVC | 0.0011 | 0.0010 | 0.0004 | 0.0004 | 0.0016 |
| 2557.1730 | KTCN | 0.0010 | 0.0010 | 0.0003 | 0.0004 | 0.0015 |
| 2557.1730 | non-ectatic control | 0.0010 | 0.0011 | 0.0004 | 0.0004 | 0.0028 |
| 2557.1730 | PMD | 0.0011 | 0.0009 | 0.0003 | 0.0004 | 0.0011 |
| 2557.1730 | PLVC | 0.0011 | 0.0011 | 0.0003 | 0.0004 | 0.0018 |

| | | | | | | |
|---|---|---|---|---|---|---|
| 2583.2339 | KTCN | 0.0010 | 0.0010 | 0.0004 | 0.0004 | 0.0022 |
| 2583.2339 | non-ectatic control | 0.0011 | 0.0011 | 0.0005 | 0.0004 | 0.0025 |
| 2583.2339 | PMD | 0.0009 | 0.0009 | 0.0004 | 0.0004 | 0.0013 |
| 2583.2339 | PLVC | 0.0010 | 0.0010 | 0.0001 | 0.0009 | 0.0014 |
| 2585.1617 | KTCN | 0.0011 | 0.0012 | 0.0007 | 0.0004 | 0.0065 |
| 2585.1617 | non-ectatic control | 0.0013 | 0.0013 | 0.0004 | 0.0004 | 0.0024 |
| 2585.1617 | PMD | 0.0007 | 0.0007 | 0.0004 | 0.0004 | 0.0011 |
| 2585.1617 | PLVC | 0.0011 | 0.0014 | 0.0011 | 0.0004 | 0.0043 |
| 2586.1507 | KTCN | 0.0011 | 0.0012 | 0.0007 | 0.0004 | 0.0057 |
| 2586.1507 | non-ectatic control | 0.0012 | 0.0014 | 0.0006 | 0.0004 | 0.0029 |
| 2586.1507 | PMD | 0.0004 | 0.0007 | 0.0006 | 0.0004 | 0.0015 |
| 2586.1507 | PLVC | 0.0011 | 0.0014 | 0.0015 | 0.0004 | 0.0055 |
| 2587.1421 | KTCN | 0.0011 | 0.0012 | 0.0006 | 0.0004 | 0.0050 |
| 2587.1421 | non-ectatic control | 0.0012 | 0.0013 | 0.0005 | 0.0004 | 0.0025 |
| 2587.1421 | PMD | 0.0011 | 0.0010 | 0.0004 | 0.0004 | 0.0012 |
| 2587.1421 | PLVC | 0.0011 | 0.0014 | 0.0012 | 0.0004 | 0.0046 |
| 2599.2657 | KTCN | 0.0010 | 0.0011 | 0.0007 | 0.0004 | 0.0035 |
| 2599.2657 | non-ectatic control | 0.0012 | 0.0014 | 0.0008 | 0.0004 | 0.0040 |
| 2599.2657 | PMD | 0.0007 | 0.0008 | 0.0004 | 0.0004 | 0.0012 |
| 2599.2657 | PLVC | 0.0010 | 0.0010 | 0.0005 | 0.0004 | 0.0021 |
| 2600.2757 | KTCN | 0.0010 | 0.0012 | 0.0009 | 0.0004 | 0.0044 |
| 2600.2757 | non-ectatic control | 0.0013 | 0.0017 | 0.0011 | 0.0004 | 0.0049 |
| 2600.2757 | PMD | 0.0009 | 0.0010 | 0.0002 | 0.0009 | 0.0013 |
| 2600.2757 | PLVC | 0.0010 | 0.0012 | 0.0005 | 0.0004 | 0.0023 |
| 2627.3039 | KTCN | 0.0010 | 0.0009 | 0.0004 | 0.0004 | 0.0024 |
| 2627.3039 | non-ectatic control | 0.0011 | 0.0011 | 0.0004 | 0.0004 | 0.0019 |
| 2627.3039 | PMD | 0.0004 | 0.0005 | 0.0002 | 0.0004 | 0.0009 |
| 2627.3039 | PLVC | 0.0011 | 0.0011 | 0.0003 | 0.0004 | 0.0014 |
| 2650.2586 | KTCN | 0.0011 | 0.0012 | 0.0005 | 0.0004 | 0.0028 |
| 2650.2586 | non-ectatic control | 0.0012 | 0.0012 | 0.0005 | 0.0004 | 0.0024 |

| | | | | | | |
|---|---|---|---|---|---|---|
| 2650.2586 | PMD | 0.0013 | 0.0012 | 0.0005 | 0.0004 | 0.0016 |
| 2650.2586 | PLVC | 0.0011 | 0.0012 | 0.0004 | 0.0004 | 0.0021 |
| 2651.2607 | KTCN | 0.0012 | 0.0013 | 0.0006 | 0.0004 | 0.0034 |
| 2651.2607 | non-ectatic control | 0.0012 | 0.0014 | 0.0006 | 0.0004 | 0.0029 |
| 2651.2607 | PMD | 0.0011 | 0.0013 | 0.0006 | 0.0009 | 0.0022 |
| 2651.2607 | PLVC | 0.0012 | 0.0012 | 0.0006 | 0.0004 | 0.0026 |
| 2652.2540 | KTCN | 0.0012 | 0.0012 | 0.0006 | 0.0004 | 0.0038 |
| 2652.2540 | non-ectatic control | 0.0012 | 0.0013 | 0.0006 | 0.0004 | 0.0028 |
| 2652.2540 | PMD | 0.0008 | 0.0010 | 0.0007 | 0.0004 | 0.0020 |
| 2652.2540 | PLVC | 0.0012 | 0.0012 | 0.0006 | 0.0004 | 0.0024 |
| 2653.2608 | KTCN | 0.0010 | 0.0011 | 0.0005 | 0.0004 | 0.0033 |
| 2653.2608 | non-ectatic control | 0.0010 | 0.0011 | 0.0004 | 0.0004 | 0.0020 |
| 2653.2608 | PMD | 0.0008 | 0.0008 | 0.0005 | 0.0004 | 0.0014 |
| 2653.2608 | PLVC | 0.0010 | 0.0010 | 0.0004 | 0.0004 | 0.0019 |
| 2674.2991 | KTCN | 0.0021 | 0.0025 | 0.0012 | 0.0009 | 0.0060 |
| 2674.2991 | non-ectatic control | 0.0027 | 0.0031 | 0.0015 | 0.0010 | 0.0064 |
| 2674.2991 | PMD | 0.0016 | 0.0019 | 0.0009 | 0.0011 | 0.0031 |
| 2674.2991 | PLVC | 0.0022 | 0.0024 | 0.0008 | 0.0014 | 0.0035 |
| 2675.3030 | KTCN | 0.0025 | 0.0030 | 0.0016 | 0.0010 | 0.0076 |
| 2675.3030 | non-ectatic control | 0.0036 | 0.0039 | 0.0020 | 0.0011 | 0.0082 |
| 2675.3030 | PMD | 0.0021 | 0.0023 | 0.0011 | 0.0013 | 0.0038 |
| 2675.3030 | PLVC | 0.0025 | 0.0028 | 0.0011 | 0.0013 | 0.0044 |
| 2676.3062 | KTCN | 0.0024 | 0.0028 | 0.0014 | 0.0010 | 0.0069 |
| 2676.3062 | non-ectatic control | 0.0033 | 0.0036 | 0.0018 | 0.0011 | 0.0075 |
| 2676.3062 | PMD | 0.0018 | 0.0020 | 0.0009 | 0.0013 | 0.0032 |
| 2676.3062 | PLVC | 0.0024 | 0.0026 | 0.0009 | 0.0013 | 0.0037 |
| 2677.3025 | KTCN | 0.0019 | 0.0022 | 0.0010 | 0.0009 | 0.0052 |
| 2677.3025 | non-ectatic control | 0.0025 | 0.0028 | 0.0013 | 0.0011 | 0.0057 |
| 2677.3025 | PMD | 0.0015 | 0.0016 | 0.0007 | 0.0010 | 0.0026 |
| 2677.3025 | PLVC | 0.0019 | 0.0021 | 0.0007 | 0.0013 | 0.0033 |

| | | | | | | |
|---|---|---|---|---|---|---|
| 2678.3062 | KTCN | 0.0015 | 0.0016 | 0.0006 | 0.0009 | 0.0033 |
| 2678.3062 | non-ectatic control | 0.0017 | 0.0019 | 0.0008 | 0.0004 | 0.0039 |
| 2678.3062 | PMD | 0.0013 | 0.0013 | 0.0003 | 0.0010 | 0.0016 |
| 2678.3062 | PLVC | 0.0014 | 0.0015 | 0.0005 | 0.0004 | 0.0020 |
| 2679.3016 | KTCN | 0.0012 | 0.0012 | 0.0004 | 0.0004 | 0.0022 |
| 2679.3016 | non-ectatic control | 0.0013 | 0.0014 | 0.0004 | 0.0004 | 0.0021 |
| 2679.3016 | PMD | 0.0011 | 0.0011 | 0.0002 | 0.0009 | 0.0013 |
| 2679.3016 | PLVC | 0.0012 | 0.0012 | 0.0004 | 0.0004 | 0.0017 |
| 2680.2991 | KTCN | 0.0010 | 0.0010 | 0.0003 | 0.0004 | 0.0017 |
| 2680.2991 | non-ectatic control | 0.0011 | 0.0011 | 0.0003 | 0.0004 | 0.0015 |
| 2680.2991 | PMD | 0.0007 | 0.0007 | 0.0003 | 0.0004 | 0.0011 |
| 2680.2991 | PLVC | 0.0011 | 0.0010 | 0.0002 | 0.0004 | 0.0012 |
| 2731.3373 | KTCN | 0.0011 | 0.0011 | 0.0004 | 0.0004 | 0.0023 |
| 2731.3373 | non-ectatic control | 0.0011 | 0.0012 | 0.0004 | 0.0004 | 0.0021 |
| 2731.3373 | PMD | 0.0010 | 0.0009 | 0.0003 | 0.0004 | 0.0011 |
| 2731.3373 | PLVC | 0.0011 | 0.0010 | 0.0004 | 0.0004 | 0.0015 |
| 2732.3330 | KTCN | 0.0010 | 0.0011 | 0.0004 | 0.0004 | 0.0026 |
| 2732.3330 | non-ectatic control | 0.0012 | 0.0013 | 0.0005 | 0.0004 | 0.0024 |
| 2732.3330 | PMD | 0.0007 | 0.0007 | 0.0003 | 0.0004 | 0.0010 |
| 2732.3330 | PLVC | 0.0012 | 0.0012 | 0.0003 | 0.0004 | 0.0016 |
| 2733.3256 | KTCN | 0.0011 | 0.0011 | 0.0003 | 0.0004 | 0.0025 |
| 2733.3256 | non-ectatic control | 0.0011 | 0.0012 | 0.0004 | 0.0004 | 0.0021 |
| 2733.3256 | PMD | 0.0010 | 0.0009 | 8.1611×10-5 | 0.0009 | 0.0010 |
| 2733.3256 | PLVC | 0.0011 | 0.0010 | 0.0004 | 0.0004 | 0.0014 |
| 2734.3338 | KTCN | 0.0010 | 0.0010 | 0.0003 | 0.0004 | 0.0019 |
| 2734.3338 | non-ectatic control | 0.0011 | 0.0011 | 0.0003 | 0.0004 | 0.0017 |
| 2734.3338 | PMD | 0.0004 | 0.0006 | 0.0003 | 0.0004 | 0.0010 |
| 2734.3338 | PLVC | 0.0010 | 0.0010 | 0.0002 | 0.0004 | 0.0013 |
| 2778.3533 | KTCN | 0.0016 | 0.0018 | 0.0008 | 0.0004 | 0.0048 |
| 2778.3533 | non-ectatic control | 0.0017 | 0.0021 | 0.0011 | 0.0009 | 0.0047 |

| | | | | | | |
|---|---|---|---|---|---|---|
| 2778.3533 | PMD | 0.0012 | 0.0012 | 0.0007 | 0.0004 | 0.0022 |
| 2778.3533 | PLVC | 0.0014 | 0.0019 | 0.0013 | 0.0004 | 0.0051 |
| 2779.3570 | KTCN | 0.0018 | 0.0022 | 0.0011 | 0.0004 | 0.0055 |
| 2779.3570 | non-ectatic control | 0.0024 | 0.0028 | 0.0014 | 0.0011 | 0.0061 |
| 2779.3570 | PMD | 0.0015 | 0.0016 | 0.0011 | 0.0004 | 0.0031 |
| 2779.3570 | PLVC | 0.0019 | 0.0023 | 0.0018 | 0.0009 | 0.0067 |
| 2780.3582 | KTCN | 0.0017 | 0.0021 | 0.0010 | 0.0004 | 0.0055 |
| 2780.3582 | non-ectatic control | 0.0022 | 0.0025 | 0.0013 | 0.0010 | 0.0056 |
| 2780.3582 | PMD | 0.0013 | 0.0014 | 0.0009 | 0.0004 | 0.0027 |
| 2780.3582 | PLVC | 0.0018 | 0.0023 | 0.0015 | 0.0010 | 0.0060 |
| 2781.3635 | KTCN | 0.0015 | 0.0017 | 0.0008 | 0.0004 | 0.0052 |
| 2781.3635 | non-ectatic control | 0.0018 | 0.0020 | 0.0009 | 0.0009 | 0.0042 |
| 2781.3635 | PMD | 0.0012 | 0.0014 | 0.0006 | 0.0010 | 0.0023 |
| 2781.3635 | PLVC | 0.0013 | 0.0017 | 0.0011 | 0.0004 | 0.0043 |
| 2782.3566 | KTCN | 0.0012 | 0.0013 | 0.0006 | 0.0004 | 0.0039 |
| 2782.3566 | non-ectatic control | 0.0013 | 0.0014 | 0.0006 | 0.0004 | 0.0028 |
| 2782.3566 | PMD | 0.0011 | 0.0010 | 0.0005 | 0.0004 | 0.0015 |
| 2782.3566 | PLVC | 0.0011 | 0.0014 | 0.0007 | 0.0004 | 0.0029 |
| 2802.2766 | KTCN | 0.0010 | 0.0010 | 0.0003 | 0.0004 | 0.0016 |
| 2802.2766 | non-ectatic control | 0.0011 | 0.0010 | 0.0002 | 0.0004 | 0.0014 |
| 2802.2766 | PMD | 0.0007 | 0.0008 | 0.0005 | 0.0004 | 0.0014 |
| 2802.2766 | PLVC | 0.0011 | 0.0011 | 0.0004 | 0.0004 | 0.0019 |
| 2803.2810 | KTCN | 0.0010 | 0.0010 | 0.0003 | 0.0004 | 0.0016 |
| 2803.2810 | non-ectatic control | 0.0011 | 0.0010 | 0.0002 | 0.0004 | 0.0014 |
| 2803.2810 | PMD | 0.0012 | 0.0010 | 0.0004 | 0.0004 | 0.0012 |
| 2803.2810 | PLVC | 0.0012 | 0.0012 | 0.0003 | 0.0009 | 0.0018 |

**Supplementary Table S6. Results of linear modelling of PC1 (a) and PC2 (b) against covariates of age, sex, diagnosis, allergy, intense eye rubbing, posterior elevation, TCT, atopy and/or asthma, smoking, and dust in the working environment (n=114)**

**a)**

| term | estimate | std.error | statistic | p.value | conf.low | conf.high |
|---|---|---|---|---|---|---|
| (Intercept) | 2,2643 | 9,8403 | 0,2301 | 0,8185 | -17,2586 | 21,7872 |
| Age | -0,1379 | 0,0679 | -2,0315 | 0,0449 | -0,2725 | -0,0032 |
| Male sex | -1,5956 | 1,7689 | -0,9020 | 0,3692 | -5,1051 | 1,9138 |
| Diagnosis of KTCN | 1,6418 | 2,5985 | 0,6318 | 0,5290 | -3,5136 | 6,7971 |
| Diagnosis of PLVC | -0,6845 | 3,4621 | -0,1977 | 0,8437 | -7,5533 | 6,1842 |
| Diagnosis of PMD | 7,4887 | 4,7040 | 1,5920 | 0,1145 | -1,8438 | 16,8213 |
| Allergy (yes) | 1,1323 | 1,7274 | 0,6555 | 0,5137 | -2,2949 | 4,5594 |
| Intense eye rubbing (yes) | 2,7417 | 1,6948 | 1,6178 | 0,1089 | -0,6206 | 6,1041 |
| Eye rubbing (yes) | -1,1764 | 1,9350 | -0,6080 | 0,5446 | -5,0155 | 2,6626 |
| Posterior Elevation | 0,0177 | 0,0265 | 0,6691 | 0,5050 | -0,0348 | 0,0703 |
| TCT | 0,0025 | 0,0170 | 0,1444 | 0,8855 | -0,0312 | 0,0361 |
| Atopy and/or asthma (yes) | -5,6954 | 2,9475 | -1,9323 | 0,0562 | -11,5431 | 0,1523 |
| Smoking (yes) | 1,8306 | 1,7544 | 1,0434 | 0,2993 | -1,6501 | 5,3114 |
| Dust in the working environment (yes) | 0,0505 | 1,8495 | 0,0273 | 0,9783 | -3,6189 | 3,7199 |

**b)**

| term | estimate | std.error | statistic | p.value | conf.low | conf.high |
|---|---|---|---|---|---|---|
| (Intercept) | -9,5658 | 5,6442 | -1,6948 | 0,0932 | -20,7637 | 1,6321 |
| Age | 0,0719 | 0,0389 | 1,8475 | 0,0676 | -0,0053 | 0,1492 |
| Male sex | 2,7992 | 1,0146 | 2,7590 | 0,0069 | 0,7863 | 4,8122 |
| Diagnosis of KTCN | 3,9864 | 1,4904 | 2,6747 | 0,0087 | 1,0295 | 6,9434 |
| Diagnosis of PLVC | 3,0310 | 1,9858 | 1,5264 | 0,1301 | -0,9087 | 6,9708 |
| Diagnosis of PMD | 2,6057 | 2,6981 | 0,9657 | 0,3365 | -2,7472 | 7,9586 |
| Allergy (yes) | 0,7307 | 0,9908 | 0,7375 | 0,4626 | -1,2351 | 2,6964 |

| | | | | | | |
|---|---|---|---|---|---|---|
| Intense eye rubbing (yes) | -0,4362 | 0,9721 | -0,4488 | 0,6546 | -2,3648 | 1,4923 |
| Eye rubbing (yes) | 0,9538 | 1,1099 | 0,8594 | 0,3922 | -1,2482 | 3,1558 |
| Posterior Elevation | -0,0169 | 0,0152 | -1,1124 | 0,2686 | -0,0470 | 0,0132 |
| TCT | 0,0084 | 0,0097 | 0,8669 | 0,3881 | -0,0109 | 0,0277 |
| Atopy and/or asthma (yes) | -2,5762 | 1,6906 | -1,5239 | 0,1307 | -5,9303 | 0,7779 |
| Smoking (yes) | -1,4605 | 1,0063 | -1,4514 | 0,1498 | -3,4570 | 0,5360 |
| Dust in the working environment (yes) | -0,1863 | 1,0608 | -0,1756 | 0,8610 | -2,2910 | 1,9184 |

**Supplementary Table S7. The details on discriminative m/z peaks from per-feature analyses.**

For each comparison, only statistically significant outcomes of the Mann–Whitney U (Wilcoxon rank-sum) test are reported, including the m/z peak, test statistics, rank-biserial effect sizes, power calculated using bootstrap, and p-values.

| Comparison | m/z peak | n1 | n2 | p.value | statistic | median1 | median2 | effsize | power_bootstrap | log2FC | p.adj |
|---|---|---|---|---|---|---|---|---|---|---|---|
| PMD vs non-ectatic controls | 2586.1507 | 4 | 44 | 0.0419 | 33.5 | 0.0004 | 0.0012 | 0.2937 | 0.591 | -1.1609 | 0.1988 |
| PMD vs non-ectatic controls | 2627.3039 | 4 | 44 | 0.0092 | 18.5 | 0.0004 | 0.0011 | 0.3759 | 0.968 | -1.0583 | 0.1532 |
| PMD vs non-ectatic controls | 1958.98889 | 4 | 44 | 0.0129 | 21.5 | 0.0004 | 0.0011 | 0.3589 | 0.898 | -1.0327 | 0.1532 |
| PMD vs non-ectatic controls | 2734.33389 | 4 | 44 | 0.0224 | 27 | 0.0004 | 0.0011 | 0.3295 | 0.764 | -1.0083 | 0.1597 |
| PMD vs non-ectatic controls | 1958.81759 | 4 | 44 | 0.0252 | 28 | 0.0007 | 0.0013 | 0.3231 | 0.697 | -0.6814 | 0.1597 |
| PMD vs non-ectatic controls | 2585.16179 | 4 | 44 | 0.0187 | 25 | 0.0007 | 0.0013 | 0.3393 | 0.799 | -0.6455 | 0.1560 |
| PMD vs non-ectatic controls | 2732.3339 | 4 | 44 | 0.0111 | 20 | 0.0007 | 0.0012 | 0.3665 | 0.929 | -0.6170 | 0.1532 |
| PMD vs non-ectatic controls | 2680.2991 | 4 | 44 | 0.0303 | 30 | 0.0007 | 0.0011 | 0.3126 | 0.657 | -0.6081 | 0.1834 |
| PMD vs non-ectatic controls | 1575.7855 | 4 | 44 | 0.0138 | 22 | 0.0011 | 0.0018 | 0.3554 | 0.846 | -0.5977 | 0.1532 |
| PMD vs non-ectatic controls | 927.4911 | 4 | 44 | 0.0402 | 33 | 0.0021 | 0.0032 | 0.2961 | 0.569 | -0.5892 | 0.1981 |
| PMD vs non-ectatic controls | 1467.8387 | 4 | 44 | 0.0252 | 28 | 0.0020 | 0.0031 | 0.3230 | 0.698 | -0.5867 | 0.1597 |
| PMD vs non-ectatic controls | 1576.7886 | 4 | 44 | 0.0124 | 21 | 0.0011 | 0.0016 | 0.3607 | 0.903 | -0.5342 | 0.1532 |
| PMD vs non-ectatic controls | 1468.8427 | 4 | 44 | 0.0402 | 33 | 0.0018 | 0.0026 | 0.2961 | 0.581 | -0.4758 | 0.1981 |
| PMD vs non-ectatic controls | 1898.9815 | 4 | 44 | 0.0170 | 24 | 0.0049 | 0.0067 | 0.3446 | 0.856 | -0.4366 | 0.1560 |
| PMD vs non-ectatic controls | 1899.9858 | 4 | 44 | 0.0188 | 25 | 0.0054 | 0.0073 | 0.3392 | 0.818 | -0.4357 | 0.1560 |
| PMD vs non-ectatic controls | 1956.1162 | 4 | 44 | 0.0124 | 21 | 0.0010 | 0.0014 | 0.3607 | 0.918 | -0.4092 | 0.1532 |
| PMD vs non-ectatic controls | 1577.7891 | 4 | 44 | 0.0131 | 21.5 | 0.0010 | 0.0013 | 0.3581 | 0.889 | -0.3896 | 0.1532 |
| PMD vs non-ectatic controls | 1901.9886 | 4 | 44 | 0.0207 | 26 | 0.0023 | 0.0030 | 0.3338 | 0.775 | -0.3859 | 0.1597 |
| PLVC vs non-ectatic controls | 1623.7807 | 10 | 44 | 0.0344 | 125 | 0.0032 | 0.0044 | 0.2879 | 0.601 | -0.4155 | 0.8804 |
| PLVC vs non-ectatic controls | 1624.783 | 10 | 44 | 0.0218 | 117 | 0.0029 | 0.0040 | 0.3121 | 0.663 | -0.4106 | 0.8804 |
| KTCN vs PMD | 1576.7886 | 93 | 4 | 0.0480 | 295 | 0.0015 | 0.0011 | 0.2008 | 0.508 | 0.4056 | 0.6404 |
| KTCN vs PMD | 1575.7855 | 93 | 4 | 0.0440 | 297 | 0.0016 | 0.0011 | 0.2045 | 0.550 | 0.4539 | 0.6404 |
| KTCN vs PMD | 2732.333 | 93 | 4 | 0.0498 | 294 | 0.0010 | 0.0007 | 0.1991 | 0.508 | 0.4608 | 0.6404 |
| KTCN vs PMD | 2627.3039 | 93 | 4 | 0.0265 | 307 | 0.0010 | 0.0004 | 0.2253 | 0.792 | 0.9014 | 0.6404 |
| KTCN vs PMD | 2734.3338 | 93 | 4 | 0.0406 | 298.5 | 0.0010 | 0.0004 | 0.2079 | 0.638 | 0.9227 | 0.6404 |

| | | | | | | | | | | | |
|---|---|---|---|---|---|---|---|---|---|---|---|
| KTCN vs PMD | 1958.9888 | 93 | 4 | 0.0246 | 309.5 | 0.0010 | 0.0004 | 0.2282 | 0.734 | 0.9574 | 0.6404 |
| KTCN vs non-ectatic controls | 2675.303 | 93 | 44 | 0.0064 | 1455 | 0.0025 | 0.0036 | 0.2328 | 0.769 | -0.4606 | 0.1276 |
| KTCN vs non-ectatic controls | 2676.3062 | 93 | 44 | 0.0090 | 1479 | 0.0024 | 0.0033 | 0.2233 | 0.772 | -0.4564 | 0.1276 |
| KTCN vs non-ectatic controls | 2779.357 | 93 | 44 | 0.0217 | 1548 | 0.0018 | 0.0024 | 0.1961 | 0.652 | -0.4062 | 0.1374 |
| INTENSE EYE RUBBING yes vs no | 1899.9858 | 43 | 86 | 0.0073 | 1312 | 0.0053 | 0.0072 | 0.2362 | 0.773 | -0.4147 | 0.1187 |
| INTENSE EYE RUBBING yes vs no | 1898.9815 | 43 | 86 | 0.0076 | 1315 | 0.0049 | 0.0066 | 0.2349 | 0.787 | -0.4003 | 0.1187 |
| INTENSE EYE RUBBING yes vs no | 1900.9876 | 43 | 86 | 0.0068 | 1307 | 0.0038 | 0.0051 | 0.2384 | 0.796 | -0.3943 | 0.1187 |
| EYE RUBBING yes vs no | 2676.3062 | 124 | 26 | 0.0493 | 1216 | 0.0024 | 0.0034 | 0.1605 | 0.561 | -0.4503 | 0.4097 |
| EYE RUBBING yes vs no | 2675.303 | 124 | 26 | 0.0366 | 1191 | 0.0026 | 0.0036 | 0.1707 | 0.525 | -0.4411 | 0.4097 |
| ATOPY and/or ASTHMA yes vs no | 2678.3062 | 12 | 137 | 0.0270 | 1139 | 0.0020 | 0.0015 | 0.1812 | 0.642 | 0.3865 | 0.3593 |
| ATOPY and/or ASTHMA yes vs no | 2677.3025 | 12 | 137 | 0.0202 | 1155 | 0.0028 | 0.0020 | 0.1903 | 0.668 | 0.4528 | 0.3593 |
| ATOPY and/or ASTHMA yes vs no | 2674.2991 | 12 | 137 | 0.0306 | 1132 | 0.0032 | 0.0022 | 0.1772 | 0.592 | 0.4872 | 0.3635 |
| ATOPY and/or ASTHMA yes vs no | 1901.9886 | 12 | 137 | 0.0247 | 1144 | 0.0040 | 0.0028 | 0.1840 | 0.620 | 0.4945 | 0.3593 |
| ATOPY and/or ASTHMA yes vs no | 1898.9815 | 12 | 137 | 0.0370 | 1121 | 0.0089 | 0.0063 | 0.1709 | 0.553 | 0.4980 | 0.3661 |
| ATOPY and/or ASTHMA yes vs no | 1900.9876 | 12 | 137 | 0.0468 | 1107 | 0.0069 | 0.0048 | 0.1629 | 0.533 | 0.5042 | 0.3661 |
| ATOPY and/or ASTHMA yes vs no | 1899.9858 | 12 | 137 | 0.0424 | 1113 | 0.0098 | 0.0066 | 0.1663 | 0.519 | 0.5545 | 0.3661 |
| ATOPY and/or ASTHMA yes vs no | 2676.3062 | 12 | 137 | 0.0230 | 1148 | 0.0038 | 0.0025 | 0.1863 | 0.639 | 0.5605 | 0.3593 |
| ATOPY and/or ASTHMA yes vs no | 2675.303 | 12 | 137 | 0.0209 | 1153 | 0.0042 | 0.0027 | 0.1892 | 0.654 | 0.6200 | 0.3593 |

**Supplementary Table S8. The top ten Spearman's rank correlations between continuous clinical variables and individual m/z peak intensities.**

| Clinical feature | m/z peak | r | n | CI_low | CI_high | p.value | p.adj |
|---|---|---|---|---|---|---|---|
| Anterior Elevation | 1467.8387 | -0.2484 | 141 | -0.4022 | -0.0863 | 0.0030 | 0.0328 |
| Anterior Elevation | 1468.8427 | -0.2435 | 141 | -0.3971 | -0.0821 | 0.0036 | 0.0328 |
| Anterior Elevation | 1469.8445 | -0.2575 | 141 | -0.4130 | -0.0945 | 0.0021 | 0.0328 |
| Anterior Elevation | 1898.9815 | -0.2766 | 141 | -0.4331 | -0.1090 | 0.0009 | 0.0328 |
| Anterior Elevation | 1899.9858 | -0.2721 | 141 | -0.4312 | -0.0879 | 0.0011 | 0.0328 |
| Anterior Elevation | 1900.9876 | -0.2726 | 141 | -0.4345 | -0.0905 | 0.0011 | 0.0328 |
| Anterior Elevation | 1901.9886 | -0.2595 | 141 | -0.4180 | -0.0847 | 0.0019 | 0.0328 |
| Anterior Elevation | 1920.9605 | -0.2442 | 141 | -0.3905 | -0.0822 | 0.0035 | 0.0328 |
| Anterior Elevation | 1921.9655 | -0.3014 | 141 | -0.4452 | -0.1530 | 0.0003 | 0.0328 |
| Anterior Elevation | 1922.9664 | -0.2491 | 141 | -0.3959 | -0.0841 | 0.0029 | 0.0328 |
| K1 | 928.4933 | -0.1424 | 145 | -0.3020 | 0.0328 | 0.0876 | 0.7261 |
| K1 | 933.5152 | -0.1719 | 145 | -0.3267 | -0.0006 | 0.0387 | 0.7261 |
| K1 | 934.5163 | -0.1469 | 145 | -0.3139 | 0.0223 | 0.0778 | 0.7261 |
| K1 | 940.4501 | -0.1384 | 145 | -0.3026 | 0.0314 | 0.0968 | 0.7261 |
| K1 | 960.5602 | -0.1220 | 145 | -0.2905 | 0.0476 | 0.1439 | 0.7261 |
| K1 | 1074.5395 | -0.1497 | 145 | -0.3108 | 0.0178 | 0.0724 | 0.7261 |
| K1 | 1138.499 | -0.1156 | 145 | -0.2694 | 0.0551 | 0.1661 | 0.7261 |
| K1 | 1467.8387 | -0.1221 | 145 | -0.2929 | 0.0455 | 0.1434 | 0.7261 |
| K1 | 1468.8427 | -0.1369 | 145 | -0.3030 | 0.0371 | 0.1007 | 0.7261 |
| K1 | 1469.8445 | -0.1615 | 145 | -0.3282 | 0.0147 | 0.0523 | 0.7261 |
| K2 | 1921.9655 | -0.2566 | 145 | -0.4044 | -0.1053 | 0.0018 | 0.2445 |
| K2 | 928.4933 | -0.1748 | 145 | -0.3349 | 0.0023 | 0.0355 | 0.2757 |
| K2 | 1467.8387 | -0.1762 | 145 | -0.3448 | -0.0103 | 0.0340 | 0.2757 |
| K2 | 1468.8427 | -0.1776 | 145 | -0.3449 | -0.0022 | 0.0326 | 0.2757 |
| K2 | 1469.8445 | -0.1899 | 145 | -0.3651 | -0.0098 | 0.0222 | 0.2757 |
| K2 | 1470.848 | -0.1938 | 145 | -0.3496 | -0.0335 | 0.0195 | 0.2757 |
| K2 | 1898.9815 | -0.2037 | 145 | -0.3629 | -0.0286 | 0.0140 | 0.2757 |
| K2 | 1899.9858 | -0.2046 | 145 | -0.3728 | -0.0337 | 0.0136 | 0.2757 |
| K2 | 1900.9876 | -0.2047 | 145 | -0.3746 | -0.0331 | 0.0135 | 0.2757 |
| K2 | 1901.9886 | -0.1834 | 145 | -0.3494 | -0.0137 | 0.0272 | 0.2757 |
| Kmax | 1921.9655 | -0.2830 | 144 | -0.4221 | -0.1384 | 0.0006 | 0.0783 |
| Kmax | 928.4933 | -0.2071 | 144 | -0.3558 | -0.0374 | 0.0127 | 0.1423 |
| Kmax | 1467.8387 | -0.2134 | 144 | -0.3737 | -0.0431 | 0.0102 | 0.1423 |
| Kmax | 1468.8427 | -0.2059 | 144 | -0.3668 | -0.0245 | 0.0133 | 0.1423 |
| Kmax | 1469.8445 | -0.2266 | 144 | -0.3895 | -0.0430 | 0.0063 | 0.1423 |
| Kmax | 1470.848 | -0.2136 | 144 | -0.3721 | -0.0541 | 0.0102 | 0.1423 |
| Kmax | 1898.9815 | -0.2262 | 144 | -0.3834 | -0.0473 | 0.0064 | 0.1423 |
| Kmax | 1899.9858 | -0.2252 | 144 | -0.3855 | -0.0479 | 0.0066 | 0.1423 |
| Kmax | 1900.9876 | -0.2262 | 144 | -0.3846 | -0.0602 | 0.0064 | 0.1423 |
| Kmax | 1901.9886 | -0.2007 | 144 | -0.3562 | -0.0277 | 0.0159 | 0.1423 |
| Posterior Elevation | 1898.9815 | -0.2882 | 140 | -0.4394 | -0.1230 | 0.0006 | 0.0155 |
| Posterior Elevation | 1899.9858 | -0.2880 | 140 | -0.4384 | -0.1149 | 0.0006 | 0.0155 |
| Posterior Elevation | 1900.9876 | -0.2872 | 140 | -0.4472 | -0.1150 | 0.0006 | 0.0155 |

| | | | | | | | |
|---|---|---|---|---|---|---|---|
| Posterior Elevation | 1921.9655 | -0.3142 | 140 | -0.4477 | -0.1602 | 0.0002 | 0.0155 |
| Posterior Elevation | 2600.2757 | -0.2871 | 140 | -0.4449 | -0.1138 | 0.0006 | 0.0155 |
| Posterior Elevation | 927.4911 | -0.2652 | 140 | -0.4105 | -0.1048 | 0.0015 | 0.0186 |
| Posterior Elevation | 928.4933 | -0.2666 | 140 | -0.4135 | -0.1075 | 0.0015 | 0.0186 |
| Posterior Elevation | 1467.8387 | -0.2632 | 140 | -0.4174 | -0.0995 | 0.0017 | 0.0186 |
| Posterior Elevation | 1469.8445 | -0.2647 | 140 | -0.4183 | -0.0992 | 0.0016 | 0.0186 |
| Posterior Elevation | 1901.9886 | -0.2714 | 140 | -0.4388 | -0.1094 | 0.0012 | 0.0186 |
| TCT | 927.4911 | 0.0823 | 147 | -0.0847 | 0.2501 | 0.3215 | 0.9983 |
| TCT | 928.49333 | 0.0876 | 147 | -0.0737 | 0.2527 | 0.2911 | 0.9983 |
| TCT | 929.4949 | 0.0367 | 147 | -0.1252 | 0.1972 | 0.6591 | 0.9983 |
| TCT | 933.5152 | 0.0325 | 147 | -0.1235 | 0.1918 | 0.6963 | 0.9983 |
| TCT | 934.5163 | 0.0094 | 147 | -0.1425 | 0.1594 | 0.9098 | 0.9983 |
| TCT | 940.4501 | -0.0373 | 147 | -0.1928 | 0.1260 | 0.6535 | 0.9983 |
| TCT | 960.5602 | 0.0522 | 147 | -0.1141 | 0.2106 | 0.5298 | 0.9983 |
| TCT | 961.5606 | 0.0187 | 147 | -0.1433 | 0.1828 | 0.8218 | 0.9983 |
| TCT | 962.5605 | 0.0502 | 147 | -0.1137 | 0.2081 | 0.5460 | 0.9983 |
| TCT | 1074.5395 | 0.0856 | 147 | -0.0816 | 0.2523 | 0.3026 | 0.9983 |

**Supplementary Table S9. Results of Spearman's rank correlation analyses between m/z peaks assigned to specific proteins and matched corneal epithelial gene-expression levels.**

| m/z peak | gene | spear_r | CI_low | CI_high | spear_p | spear_p.adj | n |
|---|---|---|---|---|---|---|---|
| 2732.333_APOH | APOH | 0.425591 | 0.058005 | 0.709968 | 0.016988 | 0.6413 | 31 |
| 2679.3016_ALB | ALB | 0.417339 | 0.038620 | 0.705167 | 0.020278 | 0.6413 | 31 |
| 2734.3338_APOH | APOH | 0.413066 | 0.013902 | 0.731945 | 0.020912 | 0.6413 | 31 |
| 2600.2757_ALB | ALB | 0.375116 | 0.017809 | 0.654429 | 0.037585 | 0.8645 | 31 |
| 2599.2657_ALB | ALB | 0.356811 | -0.000243 | 0.638513 | 0.048792 | 0.8978 | 31 |

**Supplementary Table S10. Results of WGCNA analysis for study subgroups.**

A) Module assignments of m/z peaks

| Module | Peak |
|---|---|
| turquoise | 927.4911_GOLGA2 |
| turquoise | 928.4933_GOLGA2 |
| turquoise | 929.4949_GOLGA2 |
| turquoise | 1074.5395 |
| turquoise | 1138.499_ALB |
| turquoise | 1453.8203 |
| turquoise | 1454.8085 |
| turquoise | 1467.8387_ALB |
| turquoise | 1468.8427_ALB |
| turquoise | 1469.8445_ALB |
| turquoise | 1470.848_ALB |
| turquoise | 1478.729 |
| turquoise | 1479.7321_SERPINA6 |
| turquoise | 1495.6742_HPX |
| turquoise | 1496.6791_HPX |
| turquoise | 1641.8376_ALB |
| turquoise | 1797.8849_IGKC |
| turquoise | 1798.8883_IGKC |
| turquoise | 1799.8909_IGKC |
| turquoise | 1800.8943_IGKC |
| turquoise | 1904.9665 |
| turquoise | 2585.1617 |
| turquoise | 2586.1507 |
| turquoise | 2587.1421 |
| turquoise | 2778.3533_C4B |
| turquoise | 2779.357_C4B |
| turquoise | 2780.3582_C4B |
| turquoise | 2781.3635_C4B |
| turquoise | 2782.3566_C4B |
| turquoise | 2802.2766_IGHG4 |
| turquoise | 2803.281_IGHG4 |
| brown | 933.5152_ALB |
| brown | 934.5163_ALB |
| brown | 940.4501_IGHA1 |
| brown | 960.5602_NUCB1 |
| brown | 961.5606_NUCB1 |
| brown | 962.5605 |
| brown | 1160.5839_C14orf37 |
| brown | 1161.5873 |
| brown | 1283.5692_TF |
| brown | 1311.737_ALB |
| brown | 1312.7379_ALB |
| brown | 1313.7424 |
| brown | 1910.9266_ALB |
| brown | 1911.9291_ALB |
| brown | 1912.9334_ALB |
| brown | 1913.933_ALB |
| brown | 1914.9441_ALB |
| brown | 2557.173 |
| brown | 2650.2586_ALB |
| brown | 2651.2607_ALB |
| brown | 2652.254_ALB |
| brown | 2653.2608_ALB |
| red | 1575.7855_ATP5A1 |
| red | 1576.7886_ATP5A1 |
| red | 1577.7891_ATP5A1 |
| red | 1578.7894 |
| red | 1680.7961_PDIA3 |
| red | 1681.804_PDIA3 |
| red | 1956.1162_ACTC1 |
| red | 1957.3335_ACTC1 |
| red | 1958.8175_ACTC1 |
| red | 1958.9888_TUBB |
| green | 1623.7807_ALB |
| green | 1624.783_ALB |
| green | 1625.7858_ALB |
| green | 1626.7889_ALB |
| green | 1627.7886_ALB |
| green | 1639.7759_ALB |
| green | 2045.4943_ALB |
| green | 2046.5348_ALB |
| green | 2047.5768_ALB |
| green | 2171.7616_CP |
| green | 2172.8074_CP |
| green | 2173.8434_CP |
| green | 2174.857_CP |
| green | 2175.8031_CP |
| green | 2491.267 |
| green | 2513.2352 |
| yellow | 1640.7716_ALB |
| yellow | 1699.8509 |
| yellow | 1700.8546 |
| yellow | 1701.8575 |
| yellow | 1702.8553 |
| yellow | 1742.8747_CA1 |

| | |
|---|---|
| yellow | 1743.8789_CA1 |
| yellow | 1744.8789 |
| yellow | 1745.8788 |
| yellow | 1764.8613 |
| yellow | 1765.8635 |
| yellow | 1766.8663 |
| yellow | 1780.8377_P4HB |
| yellow | 1781.8427_P4HB |
| yellow | 1782.8403_P4HB |
| yellow | 2518.194 |
| yellow | 2519.1934 |
| yellow | 2520.1957 |
| yellow | 2540.173 |
| yellow | 2541.1731 |
| yellow | 2542.1741 |
| blue | 1884.9355_BPIFA2 |
| blue | 1885.9333 |
| blue | 1886.931_BPIFA2 |
| blue | 1898.9815 |
| blue | 1899.9858 |
| blue | 1900.9876 |
| blue | 1901.9886 |
| blue | 1902.9914 |
| blue | 1903.9873 |

| | |
|---|---|
| blue | 1920.9605 |
| blue | 1921.9655_ATP5B |
| blue | 1922.9664_ATP5B |
| blue | 2545.1604_ALB |
| blue | 2546.1616_ALB |
| blue | 2547.1667_ALB |
| blue | 2583.2339 |
| blue | 2599.2657_ALB |
| blue | 2600.2757_ALB |
| blue | 2627.3039_ITIH4 |
| blue | 2674.2991_ALB |
| blue | 2675.303_ALB |
| blue | 2676.3062_ALB |
| blue | 2677.3025_ALB |
| blue | 2678.3062_ALB |
| blue | 2679.3016_ALB |
| blue | 2680.2991_ALB |
| blue | 2731.3373_APOH |
| blue | 2732.333_APOH |
| blue | 2733.3256_APOH |
| blue | 2734.3338_APOH |
| grey | 1936.9343 |
| grey | 1937.9369 |
| grey | 1938.9411_MACF1 |

B) Module descriptive statistics

| | non-ectatic control | | KTCN | | PLVC | | PMD | |
|---|---|---|---|---|---|---|---|---|
| | median | IQR | median | IQR | median | IQR | Median | IQR |
| MEblue | 0.00744 | 0.11008 | -0.02470 | 0.08070 | -0.02095 | 0.06379 | -0.05835 | 0.02858 |
| MEbrown | -0.00836 | 0.11002 | -0.01024 | 0.08612 | -0.01001 | 0.04959 | -0.05030 | 0.08959 |
| MEgreen | 0.00954 | 0.09249 | 0.00171 | 0.09762 | -0.01087 | 0.07039 | -0.01023 | 0.09475 |
| MEgrey | -0.00480 | 0.05875 | -0.00584 | 0.07351 | 0.00255 | 0.06863 | -0.06010 | 0.04991 |
| MEred | 0.00743 | 0.12212 | -0.01482 | 0.07489 | -0.01374 | 0.11082 | -0.08696 | 0.01986 |
| MEturquoise | -0.00213 | 0.08581 | -0.01756 | 0.09565 | -0.01981 | 0.07379 | -0.07367 | 0.05726 |
| MEyellow | 0.00002 | 0.10737 | 0.01715 | 0.09597 | 0.00769 | 0.05279 | 0.00560 | 0.10785 |

C) Module stability across study subgroups

| Module | statistic | p.value | method |
|---|---|---|---|
| MEblue | 11.6491 | 0.0087 | Kruskal-Wallis rank sum test |
| MEbrown | 1.1195 | 0.7724 | Kruskal-Wallis rank sum test |
| MEgreen | 2.5219 | 0.4714 | Kruskal-Wallis rank sum test |
| MEgrey | 3.4023 | 0.3337 | Kruskal-Wallis rank sum test |
| MEred | 9.2232 | 0.0265 | Kruskal-Wallis rank sum test |
| MEturquoise | 6.3842 | 0.0943 | Kruskal-Wallis rank sum test |

| | | | |
|---|---|---|---|
| MEyellow | 0.2601 | 0.9673 | Kruskal-Wallis rank sum test |

| Module | Comparison | Z | P.unadj | P.adj | Method |
|---|---|---|---|---|---|
| MEblue | Non-ectatic controls vs KTCN | 2.7854 | 0.0053 | 0.0321 | Dunn’s post-hoc test |
| MEblue | Non-ectatic controls vs PLVC | 1.0826 | 0.2790 | 1.0000 | Dunn’s post-hoc test |
| MEblue | KTCN vs PLVC | -0.3919 | 0.6952 | 1.0000 | Dunn’s post-hoc test |
| MEblue | Non-ectatic controls vs PMD | 2.5783 | 0.0099 | 0.0596 | Dunn’s post-hoc test |
| MEblue | KTCN vs PMD | 1.6387 | 0.1013 | 0.6076 | Dunn’s post-hoc test |
| MEblue | PLVC vs PMD | 1.6349 | 0.1021 | 0.6124 | Dunn’s post-hoc test |
| MEbrown | Non-ectatic controls vs KTCN | 0.5101 | 0.6100 | 1.0000 | Dunn’s post-hoc test |
| MEbrown | Non-ectatic controls vs PLVC | 0.1839 | 0.8541 | 1.0000 | Dunn’s post-hoc test |
| MEbrown | KTCN vs PLVC | -0.0868 | 0.9308 | 1.0000 | Dunn’s post-hoc test |
| MEbrown | Non-ectatic controls vs PMD | 1.0210 | 0.3073 | 1.0000 | Dunn’s post-hoc test |
| MEbrown | KTCN vs PMD | 0.8614 | 0.3890 | 1.0000 | Dunn’s post-hoc test |
| MEbrown | PLVC vs PMD | 0.7923 | 0.4282 | 1.0000 | Dunn’s post-hoc test |
| MEgreen | Non-ectatic controls vs KTCN | 1.1867 | 0.2353 | 1.0000 | Dunn’s post-hoc test |
| MEgreen | Non-ectatic controls vs PLVC | 1.1903 | 0.2339 | 1.0000 | Dunn’s post-hoc test |
| MEgreen | KTCN vs PLVC | 0.6005 | 0.5482 | 1.0000 | Dunn’s post-hoc test |
| MEgreen | Non-ectatic controls vs PMD | 0.9583 | 0.3379 | 1.0000 | Dunn’s post-hoc test |
| MEgreen | KTCN vs PMD | 0.5548 | 0.5790 | 1.0000 | Dunn’s post-hoc test |
| MEgreen | PLVC vs PMD | 0.1411 | 0.8878 | 1.0000 | Dunn’s post-hoc test |
| MEgrey | Non-ectatic controls vs KTCN | 0.9544 | 0.3399 | 1.0000 | Dunn’s post-hoc test |
| MEgrey | Non-ectatic controls vs PLVC | -0.7598 | 0.4474 | 1.0000 | Dunn’s post-hoc test |
| MEgrey | KTCN vs PLVC | -1.3246 | 0.1853 | 1.0000 | Dunn’s post-hoc test |
| MEgrey | Non-ectatic controls vs PMD | 1.2001 | 0.2301 | 1.0000 | Dunn’s post-hoc test |
| MEgrey | KTCN vs PMD | 0.8853 | 0.3760 | 1.0000 | Dunn’s post-hoc test |
| MEgrey | PLVC vs PMD | 1.5093 | 0.1312 | 0.7874 | Dunn’s post-hoc test |
| MEred | Non-ectatic controls vs KTCN | 1.8460 | 0.0649 | 0.3893 | Dunn’s post-hoc test |
| MEred | Non-ectatic controls vs PLVC | 0.5785 | 0.5629 | 1.0000 | Dunn’s post-hoc test |
| MEred | KTCN vs PLVC | -0.4060 | 0.6848 | 1.0000 | Dunn’s post-hoc test |
| MEred | Non-ectatic controls vs PMD | 2.7743 | 0.0055 | 0.0332 | Dunn’s post-hoc test |
| MEred | KTCN vs PMD | 2.1758 | 0.0296 | 0.1774 | Dunn’s post-hoc test |
| MEred | PLVC vs PMD | 2.1064 | 0.0352 | 0.2110 | Dunn’s post-hoc test |
| MEturquoise | Non-ectatic controls vs KTCN | 1.8759 | 0.0607 | 0.3640 | Dunn’s post-hoc test |

| | | | | | |
|---|---|---|---|---|---|
| MEturquoise | Non-ectatic controls vs PLVC | 1.1170 | 0.2640 | 1.0000 | Dunn’s post-hoc test |
| MEturquoise | KTCN vs PLVC | 0.1444 | 0.8852 | 1.0000 | Dunn’s post-hoc test |
| MEturquoise | Non-ectatic controls vs PMD | 2.0409 | 0.0413 | 0.2475 | Dunn’s post-hoc test |
| MEturquoise | KTCN vs PMD | 1.4151 | 0.1570 | 0.9423 | Dunn’s post-hoc test |
| MEturquoise | PLVC vs PMD | 1.1402 | 0.2542 | 1.0000 | Dunn’s post-hoc test |
| MEyellow | Non-ectatic controls vs KTCN | -0.5077 | 0.6116 | 1.0000 | Dunn’s post-hoc test |
| MEyellow | Non-ectatic controls vs PLVC | -0.1679 | 0.8666 | 1.0000 | Dunn’s post-hoc test |
| MEyellow | KTCN vs PLVC | 0.1024 | 0.9184 | 1.0000 | Dunn’s post-hoc test |
| MEyellow | Non-ectatic controls vs PMD | -0.1652 | 0.8688 | 1.0000 | Dunn’s post-hoc test |
| MEyellow | KTCN vs PMD | 0.0130 | 0.9896 | 1.0000 | Dunn’s post-hoc test |
| MEyellow | PLVC vs PMD | -0.0464 | 0.9630 | 1.0000 | Dunn’s post-hoc test |

D) Module similarity between study subgroups (correlation coefficients)

| Subgroup1 | Subgroup2 | Pearson r | Pearson p-value | Spearman ρ | Spearman p-value |
|---|---|---|---|---|---|
| non-ectatic control | KTCN | -0.8934 | 0.0067 | -0.7857 | 0.0480 |
| non-ectatic control | PLVC | -0.2748 | 0.5509 | -0.3929 | 0.3956 |
| non-ectatic control | PMD | -0.8725 | 0.0104 | -0.8214 | 0.0341 |
| KTCN | PLVC | -0.1624 | 0.7280 | 0.0000 | 1.0000 |
| KTCN | PMD | 0.7762 | 0.0402 | 0.6429 | 0.1389 |
| PLVC | PMD | 0.0918 | 0.8447 | 0.1429 | 0.7825 |

## SUPPLEMENTARY FIGURES

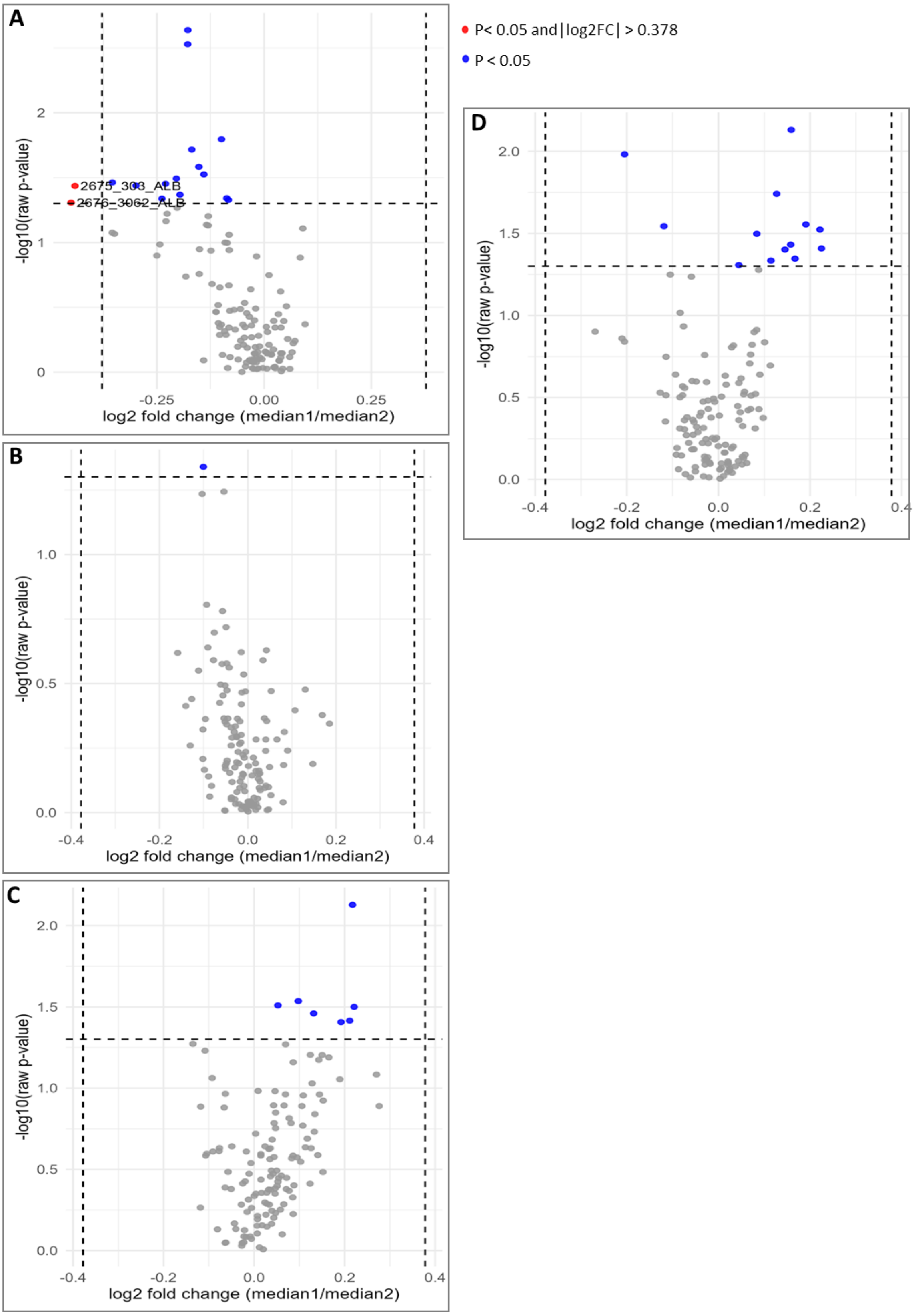


**Supplementary Figure S1.** Volcano plots of differential m/z peaks of serum proteomic data. **A**) Stratification by eye-rubbing behavior (‘yes’, *n*=124; ‘no’, *n*=26). **B**) Stratification by

allergy status (‘yes’, *n*=37; ‘no’, *n*=112). **C**) Stratification by smoking status (‘yes’, *n*=33; ‘no’, *n*=112). **D**) Stratification by sex (‘M’, *n*=105; ‘F’, *n*=46). For each comparison, Mann–Whitney U (Wilcoxon rank-sum) tests were performed, and log2 fold-change (log2FC) values were calculated using subgroup medians. The red dots represent specific m/z peaks fitting the criteria of $|\log2FC| > 0.3785$ (corresponding to ≥1.3-fold increases or ≤1/1.3-fold decreases) and p-value $< 0.05$, while the blue dots denote specific m/z peaks fitting only the criterion of p-value $< 0.05$. See Supplementary Table S7 for more details.

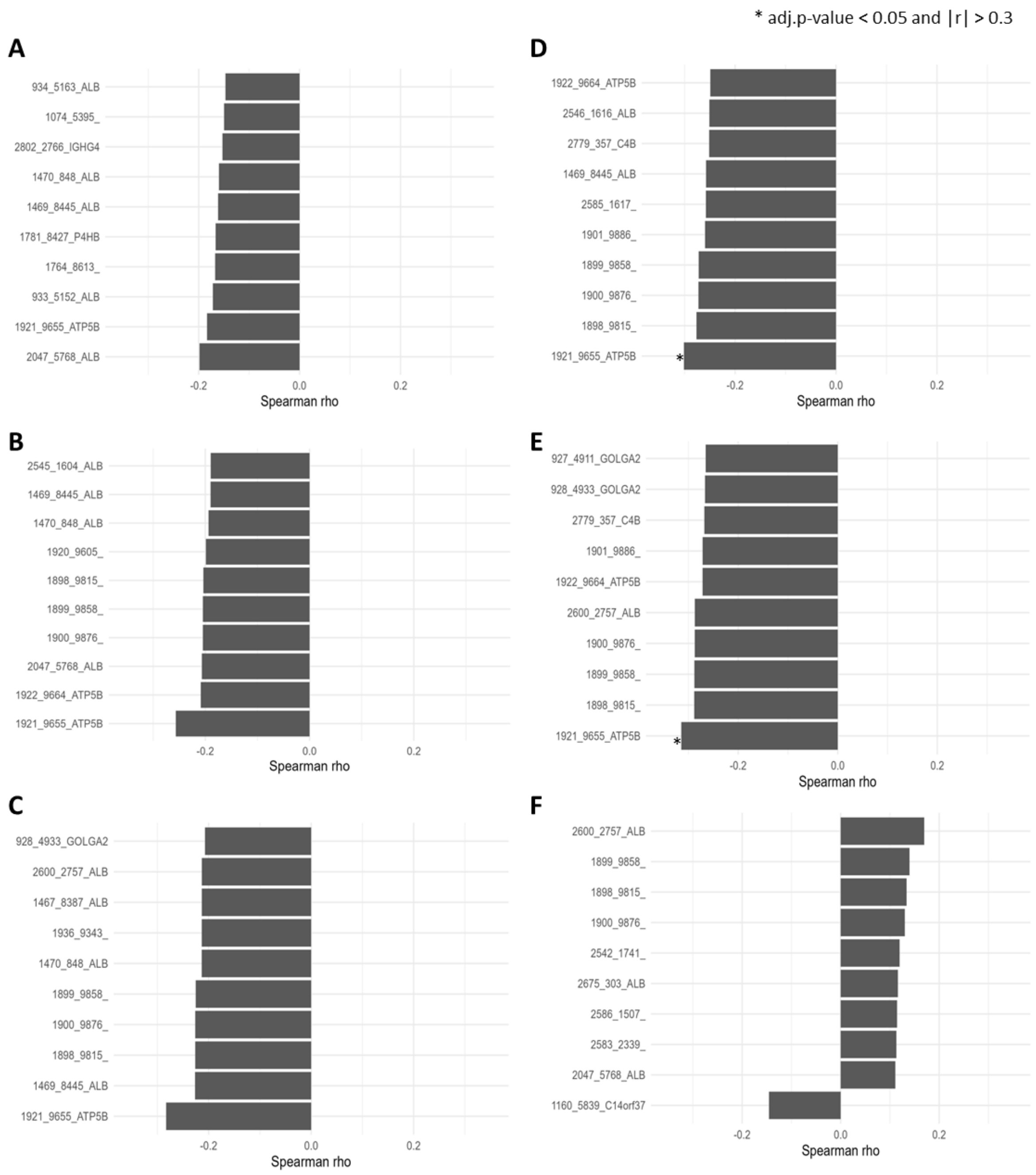


**Supplementary Figure S2.** Bar plots depicting Spearman rank correlation coefficients between quantitative clinical parameters and the intensities of individual serum m/z peaks. Clinical parameters were taken from the eye with more advanced corneal disease (for non-ectatic control individuals, the eye exhibiting the greatest deviation from normative values, please see Methods). Panels display correlations for **A)** flat keratometry (K1, *n*=145), **B)** steep keratometry (K2, *n*=145), **C)** maximal corneal curvature (Kmax, *n*=144), **D)** anterior corneal elevation (*n*=141), **E)** posterior corneal elevation (*n*=140), and **F)** thinnest corneal thickness (TCT, *n*=147). Correlations meeting the predefined significance criteria (absolute correlation

coefficient $|r| > 0.3$ and Benjamini–Hochberg FDR-adjusted $p < 0.05$) are annotated with an asterisk.

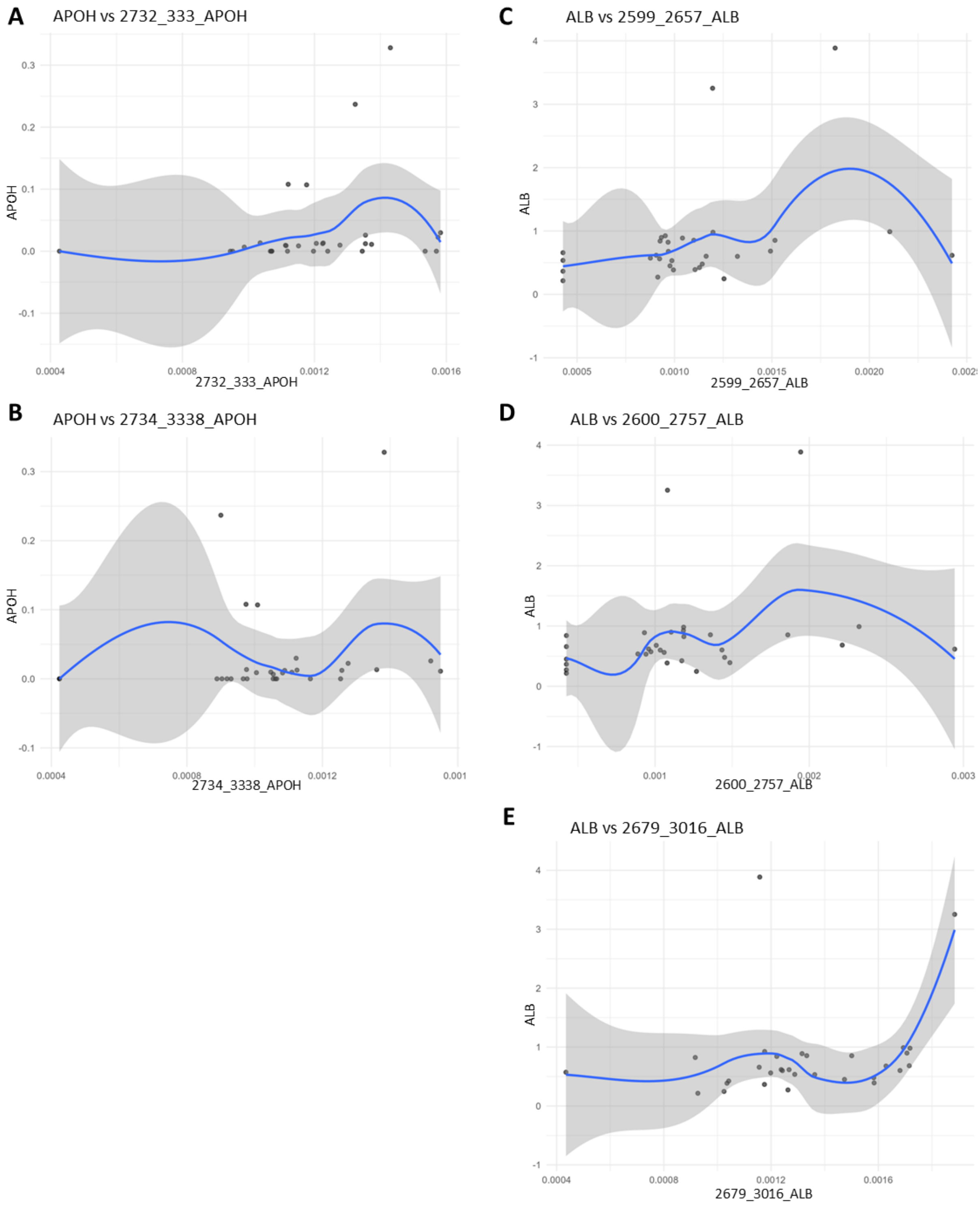


**Supplementary Figure S3.** Scatter plots depicting Spearman rank correlation coefficients between gene expression in corneal epithelium and the intensities of individual serum m/z peaks. Gene expression (in TPM, *n*=31) were taken from the *central topographic region* of the corneal epithelium of the eye with more advanced corneal disease (for non-ectatic control individuals, the eye exhibiting the greatest deviation from normative values, please see Methods). Panels display correlations for **A)** m/z peak of 2732.33303730667 and APOH, **B)**

m/z peak of 2734.33383870487 and APOH, **C)** m/z peak of 2599.26567787107 and ALB, **D)** m/z peak of 2600.27568934846 and ALB, and **E)** m/z peak of 2679.30159591715 and ALB. Correlations meeting the predefined significance criteria (absolute correlation coefficient $|r| > 0.3$ and p-value $< 0.05$) are annotated with an asterisk.
